# Simultaneous genetic transformation and genome editing of mixed lines in soybean (*Glycine max*) and maize (*Zea mays*)

**DOI:** 10.1007/s42994-024-00173-5

**Published:** 2024-06-18

**Authors:** Michelle Valentine, David Butruille, Frederic Achard, Steven Beach, Brent Brower-Toland, Edward Cargill, Megan Hassebrock, Jennifer Rinehart, Thomas Ream, Yurong Chen

**Affiliations:** Bayer Crop Science, 700 Chesterfield Parkway W, Chesterfield, MO 63017 USA

**Keywords:** Maize, Soybean, Seed embryo explants, TREDMIL, Genetic transformation, Genome editing

## Abstract

Robust genome editing technologies are becoming part of the crop breeding toolbox. Currently, genome editing is usually conducted either at a single locus, or multiple loci, in a variety at one time. Massively parallel genomics platforms, multifaceted genome editing capabilities, and flexible transformation systems enable targeted variation at nearly any locus, across the spectrum of genotypes within a species. We demonstrate here the simultaneous transformation and editing of many genotypes, by targeting mixed seed embryo explants with genome editing machinery, followed by re-identification through genotyping after plant regeneration. Transformation and Editing of Mixed Lines (TREDMIL) produced transformed individuals representing 101 of 104 (97%) mixed elite genotypes in soybean; and 22 of 40 (55%) and 9 of 36 (25%) mixed maize female and male elite inbred genotypes, respectively. Characterization of edited genotypes for the regenerated individuals identified over 800 distinct edits at the Determinate1 (*Dt1*) locus in samples from 101 soybean genotypes and 95 distinct Brown midrib3 (*Bm3*) edits in samples from 17 maize genotypes. These results illustrate how TREDMIL can help accelerate the development and deployment of customized crop varieties for future precision breeding.

**Supplementary Information:**

The online version contains supplementary material available at 10.1007/s42994-024-00173-5.

## Introduction

To feed a growing global population under ever-changing climate conditions, plant scientists are challenged with developing and adopting innovative technologies to improve existing crops and develop new crops (Hickey et al. 2019). Advanced breeding technologies, such as marker-assisted and genome-wide selection have improved speed while reducing the cost of culling inferior progeny and have decreased reliance on phenotypic selection (Bernardo and Yu 2007; Bassi et al. 2015). However, traditional plant breeding based upon crossing and selection, leveraging existing variation and random mutations, appears to be reaching its limits in some crops (Hickey et al. 2019). The CRISPR (Clustered Regularly Interspaced Short Palindromic Repeats)/Cas-based editing technology, with its capacity to direct variation to selected targets across genomes, holds great promise to revolutionize plant breeding and make a substantial contribution to global food security. CRISPR/Cas-based editing has been shown to be effective in editing simple traits (Shan et al. 2013) and complex traits (Gao et al. 2020) in a variety of plant species. Furthermore, this technology has recently been applied to genome-scale editing in several species, including rice, maize, and soybean, to generate genetic variability for trait gene identification and crop improvement (Bai et al. 2020; Li et al. 2022; Liu et al. 2020; Luo et al. 2021). Introducing novel variability, particularly in genomic regions with limited variation, can unleash genetic gain potential that is currently untapped by conventional breeding. The combination of robust genomics platforms, and multifaceted genome editing capabilities, paired with high-throughput genotype-flexible plant transformation systems, could enable creation of targeted variation at nearly any locus across diverse germplasm within a species. Together, these capabilities would help drive efficient and effective crop development at an unprecedented pace by ushering genetic improvement into a new era of precision breeding (Chen et al. 2019).

Although the crop breeding toolbox has been notably enriched by genomics and genome editing technologies, commensurate advances in plant transformation technology are currently lagging (Altpeter et al. 2016). Since the first demonstration of successful plant transformation in the mid-1980s, improvements have been made, including the development of target explants from suspension cultures to callus cultures to immature embryos. In addition, a seed embryo explant-based meristem transformation system that leverages organogenesis-based regeneration has been developed in dicot species, such as soybean and cotton (Chen et al. 2014, 2020; Ye et al. 2008), as well as monocot species like maize (Ye et al. 2022). It is rapid, high-throughput and automation-friendly. However, plant transformation and regeneration have remained largely genotype-dependent. Recently, developmental regulators such as BABY BOOM (BBM) and WUSHEL (WUS) (Lowe et al. 2016), GROWTH REGULATING FACTORS (GRFs), GRF-INTERACTING FACTORS (GIF) and GRF-GIF CHIMERAS (Debernardi et al. 2020; Kong et al. 2020), WUSHEL-RELATED HOMEOBOX5 (WOX5) (Wang et al. 2022) or PLETHORA (LPT5) (Lian et al. 2022) have been shown to promote somatic embryogenesis, thereby improving regeneration in several monocot and dicot species and enabling more successful transformation of recalcitrant genotypes. However, there can be limits to the use of these developmental regulator-enhanced regeneration systems, for instance in species where they work well only with few genotypes, or when somatic embryogenesis-based regeneration is challenging to scale. The use of organogenesis-based regeneration systems or seed embryo explant-based meristem transformation can provide an alternative in the face of such challenges (Chen et al. 2014; Ge et al. 2023; Ye et al. 2011, 2022). Yet, further improvements in such plant transformation methods are needed to expand the reach and scope of genome editing as a next generation plant breeding technology (Kausch et al. 2019).

The soybean Dt1 and the maize BM3 loci were selected to demonstrate the potential for improved plant transformation methods in conjunction with editing technologies to improve deployment strategies for genome edits in this new era of precision breeding. Maize bm3 mutants present a brown-reddish pigmentation on the midrib of the leaf, between developmental stages V4–V6, which will disappear prior to maturity. This is due to an alteration in the activity of the caffeic acid O-methyl transferase gene, which reduces lignin synthesis and improves the digestibility of corn stover for silage (Sattler et al. 2010). Several known bm3 mutants are deletions, making BM3 an excellent target for proof-of-concept gene editing. The soybean Dt1 locus is a well-conserved breeding target controlling stem growth habit. Allelic variation in Dt1 among cultivated soybean lines is limited to the partially dominant wild-type allele GmTFL1b that leads to indeterminate growth habit, and one recessive mutant allele (Gmtfl1b-ab) arising from a non-synonymous substitution in the coding sequence of the causal gene that produces determinate stem growth (Tian et al. 2010). Aside from the single SNP in the first exon of the coding sequence, no other SNP variation has been found in the proximal promoter or coding sequence between these two alleles among the cultivated soybean lines that have been sequenced. This provides a unique opportunity to introduce variation through genome editing at this locus in diverse germplasm.

In this study, we report a method of simultaneous transformation and editing of numerous genotypes by mixing the lines (genotypes) prior to the production of seed embryo explants, termed Transformation and Editing of Mixed Lines (TREDMIL). The mixed lines are transformed with genome editing machinery by the seed embryo explant-based meristem transformation system. After regeneration, line identity is deconvoluted via genotyping. Elite genotypes of soybean and maize were used to demonstrate this concept. The successful implementation of TREDMIL could: (a) increase the production efficiency of transgenic and edited events for testing, (b) replace or complement the traditional trait deployment approach of introgression through backcrossing when most germplasm of a crop can be successfully transformed, or edited, and (c) enable earlier evaluation of germplasm-by-trait interactions in a trait discovery pipeline. Although we have conducted this experiment at a relatively large scale with more than 100 genotypes, there is no conceptual reason why TREDMIL could not be scaled up or down to meet the needs and constraints of a given project.

## Results

### TREDMIL is a technology for transforming and editing many germplasm in a single experiment

**Fig. 1:**
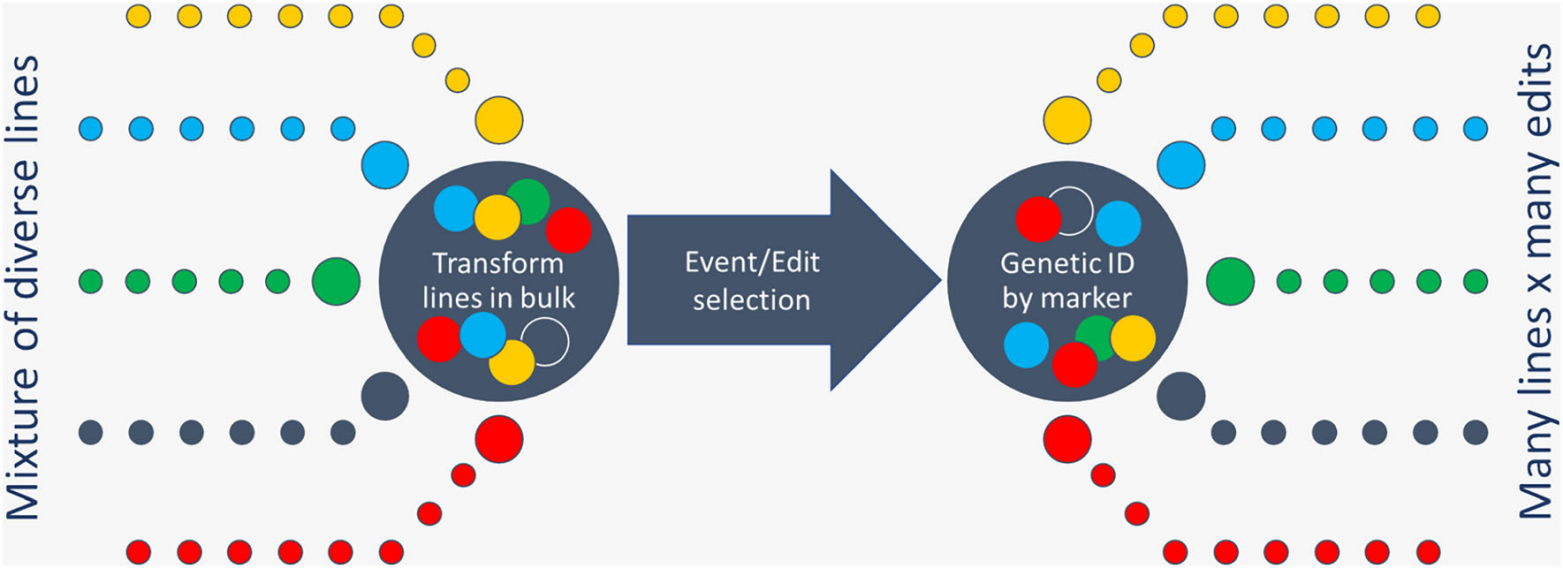
Cartoon illustrating the Transformation and Editing of Mixed Lines (TREDMIL) concept. A mixture of seed from diverse lines is composed and prepared for transformation and editing through automated explant isolation. Following transformation and regeneration, plants are re-identified through genotyping and their edits characterized through genotyping or sequencing

The concept of mixing germplasm in bulk prior to transformation arose from the need for a plant transformation and genome editing pipeline that is scaled to match the requirements of a robust breeding program (Fig. 1). The material from a mixed germplasm can be batch-processed through multiple workflow steps without stringent identity preservation. This can reduce the cost of handling and processing diverse germplasm through a transformation process or enable greater numbers of distinct germplasm within the same transformation resource capacity. Recovered plants, regenerated from the transformation process, can be genetically differentiated through low-cost, marker-based fingerprinting. Bayer’s seed embryo explant-based meristem transformation system (Ye et al. 2011, 2022; Chen et al. 2014) paired with existing genomic fingerprinting technologies enables the TREDMIL approach.

For our demonstration of the TREDMIL approach, we have focused on application of this method to a large acre dicot (soybean) and monocot (maize) crop. We have directed creation of indels to the targeted genic regions in this experiment through co-expression of gene-specific CRISPR RNAs (crRNAs) and the editing nuclease Cas12a. Cas9 and Cas12a nucleases, derived from different bacterial species, function similarly, creating sequence-specific double-stranded lesions at locations complementary to coexpressed crRNA, differing primarily in cutting frequency and deletion size after lesion repair (Banakar et al. 2020). Although the creation of phenotypic data from edited alleles generated in this work would have been satisfying, the scale and purpose of these experiments did not justify the additional resources necessary to maintain generated plants through maturity and seed harvest. For the same reason, we did not edit and genotype multiple genomic targets in this experiment, although TREDMIL could facilitate the simultaneous editing of more than one target as well.

### Simultaneous transformation of 101 elite soybean genotypes

To demonstrate the TREDMIL concept in soybean, seeds from 104 elite lines representing maturity groups (MG) 00 to VII were combined into a single seed mix prior to explant isolation. Seed embryo explants were excised through an automated process and used for transformation in a single experiment. In this experiment, approximately 72,000 explants of 104 mixed soybean genotypes were transformed by *Agrobacterium* strain AB30 harboring the pM206 editing construct that co-expresses *aadA* as the selectable marker, a Cas12a nuclease and 3 CRISPR RNAs (crRNAs) targeting non-coding promoter sequence upstream of the gene at *Determinate1* (*Dt1*) (Fig. S1A and S1B). Transformation was initiated with 36,000 explants on two separate dates and 1008 individuals were sampled for molecular assays from regenerated R0 plants produced by each initiation, for a total of 2016 individuals. DNA from leaf samples collected on soybean plantlets were used to deconvolute line identities using marker-based genotyping. Transgene copy number assays were employed to analyze transformation frequencies and targeted-allele sequencing was performed to evaluate editing outcomes.

To deconvolute individual soybean line identities, DNA of leaf samples from 2016 regenerated plants were analyzed using 50 genetic markers to identify combinations of unique single nucleotide polymorphisms (SNPs) that are diagnostic of individual lines present within the originating seed mix. Using 1971 samples that returned SNP marker genotyping data from the original 2016 submitted samples, a total of 94% (*n* = 1856/1971) of the samples were initially assigned line identities with greater than 90% match to a given line, which included filtering of this data to remove samples with fewer than 30 marker datapoints (40% missing data) or samples with heterozygosity greater than 20%. Of 2016 samples submitted for TaqMan genotyping for the Mt. Ac145767 3′UTR in the Cas12a cassette (indicative of being a transformed event), 1957 samples returned a copy number of 1 or higher. The intersection of these two datasets produced a total of 1809 events that were subsequently used for determining the transformation frequencies by line and maturity group.

**Fig. 2:**
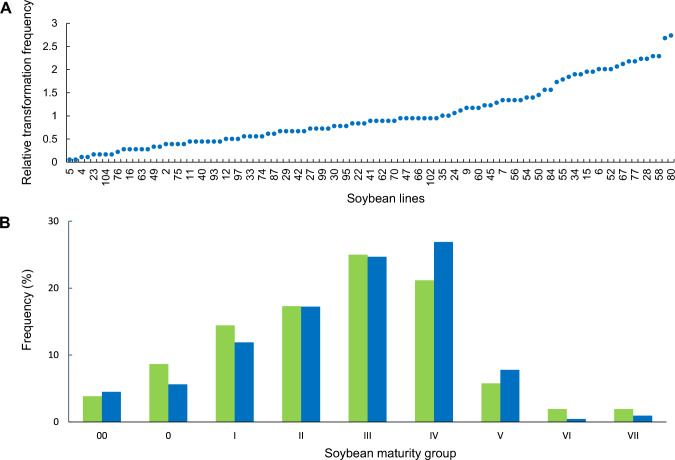
Simultaneous transformation of 101 elite soybean lines from diverse maturity groups and genetic backgrounds. **A** Relative transformation frequency score by individual soybean line (blue dots). For soybean, transformation frequency is defined as the percentage of inoculated embryo explants producing events having selectable marker gene. Relative transformation frequency score of each line in the mix is the ratio of transformation frequency of each line in the population divided by the average transformation frequency of the mixed population. The complete order of lines listed on the *x*-axis is presented in Supplemental Table S1. **B** (Green bars) The percentage of mix for a given maturity group is defined as the number of lines within a maturity group divided by the total number of lines in the mix. (Blue bars) Percent of total events for a maturity group is calculated as the number of events represented across any given maturity group divided by the total number of events

For those soybean transformants with assigned line identity, 101 out of 104 elite genotypes (97%) were identified to be simultaneously transformed (Fig. 2A), indicating genotype flexibility of the seed embryo transformation system with soybean. The first initiation produced events from 100 out of 104 lines and the second initiation produced events in 98 out of 104 lines. We observed a 75% correlation between the two initiations in the transformation frequency across the 101 lines that produced events. Altogether, this indicated high reproducibility of transformation across lines on different transformation initiation days. Because of the high correlation across these two independent initiations, we combined these data from both initiations for all subsequent analyses.

Across lines, we observed a range of transformation frequencies as indicated by relative transformation frequency score of each line in Fig. 2A. These results indicate that there is likely to be some germplasm dependence in transformation frequency. In this experiment, recovered transformants were distributed across diverse maturity groups ranging from 00 to VII. For most maturity groups, the percentage of transgenic events recovered was similar to the percentage of that maturity group in the bulk mix (Fig. 2B). This indicates that any germplasm dependent effects in genotype flexible soybean transformation is nonetheless maturity group independent.

### Simultaneous transformation of 22 elite maize female inbreds

To demonstrate TREDMIL in maize, seeds from 78 elite inbred lines (*n* = 40 female, *n* = 38 male; 78 total) and 500 F1 populations (*n* = 250 each for female and male populations; 500 total) representing relative maturities (RM) 90–120 were mixed prior to explant isolation. The female, or Stiff-Stalk, and male, or Non-Stiff-Stalk, denominations refer to the two dominant heterotic groups in elite temperate maize germplasm. Seed embryo explants were excised through an automated process and used for transformation. In five independent experiments, approximately 65,000 explants mixed from 78 inbreds and 500 F1s were transformed with *Agrobacterium* strain AB32 containing genome editing construct pM795 with selectable marker gene epsps cp4, a Cas12a nuclease and three CRISPR RNAs (crRNAs) targeting *Brown midrib3* (*Bm3*) (Fig. S2A and S2B). A total of 139 plantlets were regenerated and leaf samples were collected from all transferred maize plantlets for the deconvolution of line identities via genotyping, transgene copy number, and genome editing assays as described for soybean.

**Fig. 3:**
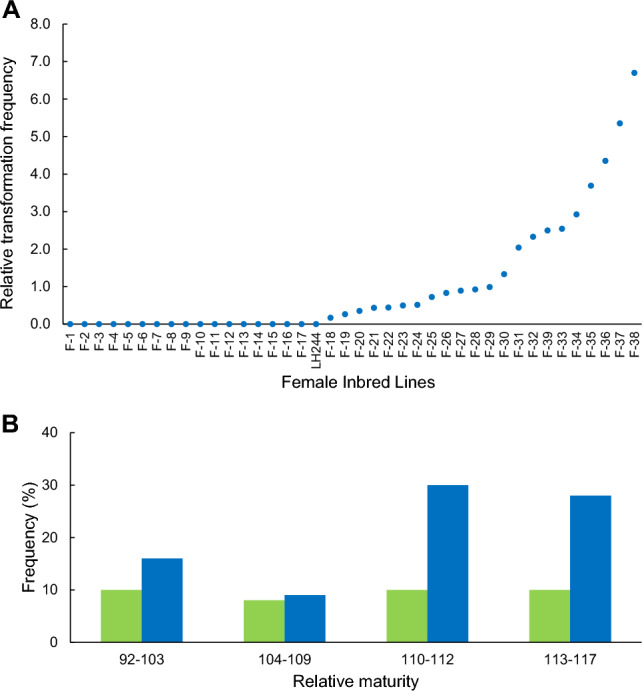
Simultaneous transformation of elite maize female lines in diverse relative maturities and genetic backgrounds. **A** Relative transformation frequency score by individual maize line. For maize, transformation frequency is defined as the percentage of inoculated embryo explants producing non-chimeric events having selectable marker gene. Relative transformation frequency score of each line in the mix is the ratio of transformation frequency of each line in the population over the overall transformation frequency of the mixed population. **B** Transformation frequency by relative maturity as a function of proportion in the mix (green bars) and total events (blue bars)

Identification of maize lines and populations from DNA of leaf samples of the regenerated plantlets (*n* = 139) was similarly performed using 26 genetic markers. In this experiment, out of the 139 plantlets, the following was ascertained: 124 were from female inbreds, 1 was from a male inbred, 13 were from female F1 populations, and 1 was a male F1 population. Overall, the regenerated maize plantlet identities were assigned to 22 female inbreds, one male inbred, 11 female F1 populations, and one male F1 population for 35 total unique pedigrees transformed. Results for the five independent experimental replications were combined, as each replication produced relatively few events, and overall transformation and editing efficiencies between experiments were consistent. As shown in Fig. 3A, 22 out of 40 (55%) elite female inbreds were simultaneously transformed with various relative transformation frequency scores, showing some genotype flexibility of seed embryo transformation with maize female inbreds. In addition, transformants were distributed across diverse relative maturities (RM) ranging from 92 to 117. The percentage of bulk mix in a particular RM was similar to the percentage of total transgenic events recovered for the first two RM bins. However, later RM groups were over-represented as a function of total events because more of the female lines included in this experiment were in those RMs.

### Simultaneous transformation of nine elite maize male inbreds

**Fig. 4:**
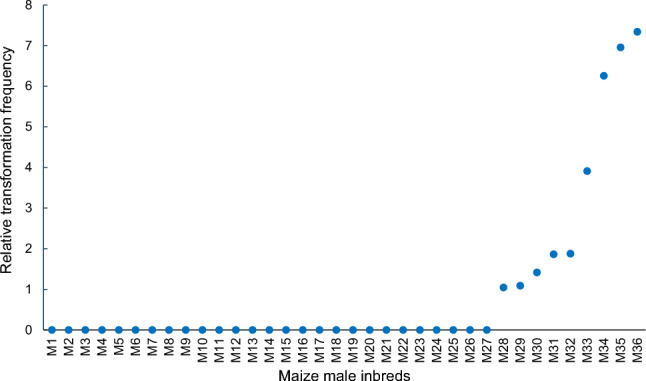
Simultaneous transformation of elite maize male lines in a diverse genetic background. Transformation frequency is defined as the percentage of inoculated embryo explants producing non-chimeric events having selectable marker gene. Relative transformation frequency score of each line in the mix is the ratio of transformation frequency of each line in the population over the overall transformation frequency of the mixed population

In the first transformation experiment with mixed maize germplasm, maize male inbreds were shown to be more recalcitrant to transformation than female inbreds as only 1 out 38 male inbreds produced transformed events. In the second experiment, seeds from 36 maize elite male inbreds from RM 110–112 were mixed before embryo explant excision as described above. A total of 62,000 explants mixed from 36 male maize inbreds were transformed with *Agrobacterium* AB32 carrying construct pM284 that has the same selectable marker gene epsps-cp4 but has a uidA reporter gene instead of genome editing machinery (Fig. S3). A total of 22 plantlets were regenerated and plantlet identities were assigned to 9 unique male inbred lines using the same set of 26 genetic markers. As shown in Fig. 4, 9 out of 36 (25%) elite male inbreds were simultaneously transformed with various relative transformation frequency scores. In this study, a higher percentage of male inbreds was transformed in the second experiment than the first experiment. This could be due to the fact that seeds were grown under the same conditions and explants were processed from recently harvested seeds for the second experiment, and that the number of explants in each inbred used for transformation was larger in the second experiment than the first experiment. In contrast, in the first experiment, explants were excised from seeds grown at different year and stored under the different conditions for different durations.

### Simultaneous editing of 101 elite soybean genotype and 17 maize female inbreds

**Fig. 5:**
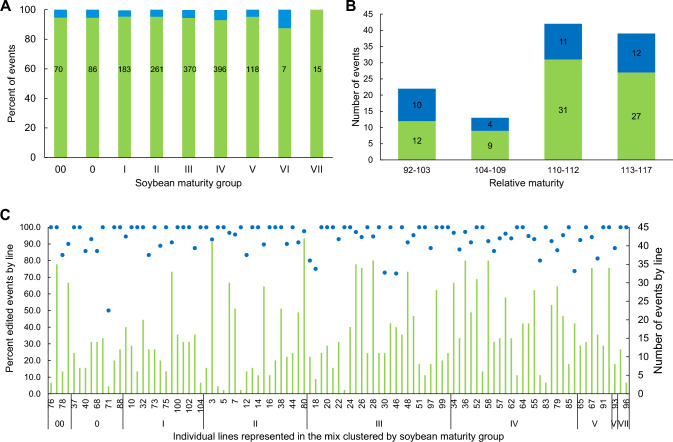
Simultaneous editing of multiple elite soybean and maize with no obvious bias. **A** Editing frequency by soybean maturity group. Green bars represent the percent of edited lines within each maturity group, and the blue bars represent the percent of non-edited events within each maturity group. The number value indicates the total number of edited events within each maturity group. **B** Editing frequency by maize relative maturity. Green bars represent the number of edited lines within each maturity group, and the blue bars represent the number of non-edited events within each maturity group. **C** Editing frequency within each soybean line expressed as the percentage of edited lines calculated from the total events for a given line (dots, left axis) shown with the number of events from each soybean line (green bars, right axis), ordered by relative maturity. The complete order of lines listed on the *x*-axis is presented in Supplemental Table S1

Amplicon sequencing was performed using DNA from early vegetative leaf tissue for the soybean *Dt1* crRNA target sites and for the maize *Bm3* crRNA target sites in the respective samples. 1787 out of 2016 soybean DNA samples produced amplicon sequencing data at the Dt1 target site. The intersection of this dataset with the 1858 samples that could be assigned a line identity and the 1957 samples producing a copy number call of 1 or higher for the transgene resulted in a final dataset of 1596 events that were used in the subsequent editing analyses. Edits at *Dt1* target sites were detected in 94% (*n* = 1506/1596) of soybean events across 101 of the 104 lines comprising the original bulk mix and representing all 101 transformed lines (Fig. 5A). As shown in Fig. 5C, 81 of the 101 transformed soybean lines produced edits in greater than 90% of the events produced by that line, indicating highly efficient, genotype flexible genome editing with the seed embryo transformation system.

Edits at *Bm3* target sites were characterized in 69% (*n* = 96/139) of maize events analyzed and represented a total of 26 unique pedigrees, including 17 female inbreds, 1 male inbred, and eight female F1 populations. For female inbreds, only 17 out of 22 transformed lines were found to be edited, consistent with the observation that some of transformed lines were represented by a very limited number of events. Like the frequency of transformants, editing events were over-represented in higher RMs (Fig. 5B).

### Editing at soybean Dt1 and maize Bm3 target sites

We examined the editing outcome of TREDMIL experiments at crRNA target sites Dt1 and Bm3 to understand editing outcome across diverse germplasm. The soybean Dt1 locus is a well-conserved breeding target controlling stem growth habit (Tian et al. 2010) with limited allelic variation among cultivated soybean lines with available sequence data, making it an excellent case-in-point for targeting novel variation across numerous genotypes in a crop. The maize Bm3 locus is also a breeding target for reducing lignin synthesis to improve digestibility of stover for silage with reports available for several mutant alleles (Sattler et al. 2010). Across three *Dt1* target sites, 1506 events produced over 800 distinct edits and over 4000 total edits, consistent with the observation that some events were chimeric and contained multiple edits within the sampled leaf (Supplementary Table S2A). Across three *Bm3* target sites, 96 events produced 95 distinct edits and over 200 total edits (Supplementary Table S2B).

**Fig. 6:**
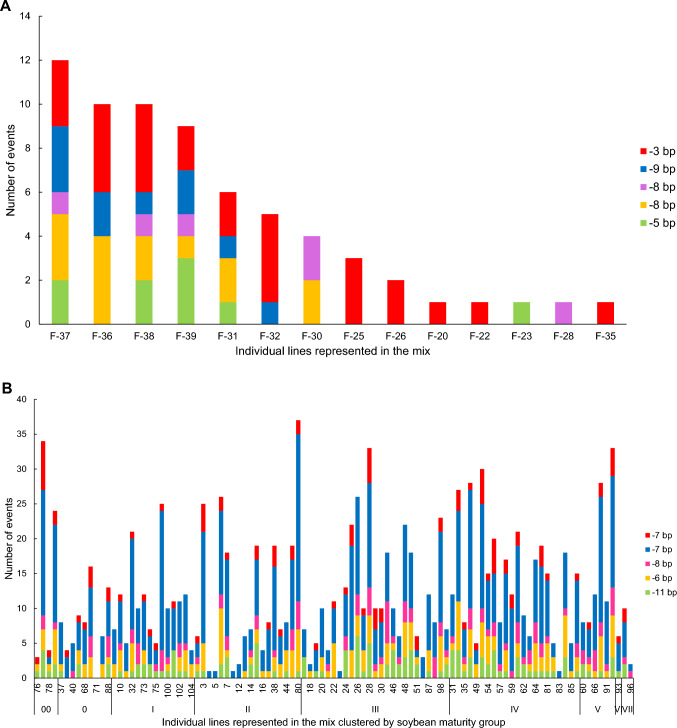
Distribution of distinct edits across maize and soybean lines. **A** On the *x*-axis are each of the transformed female corn lines, ordered by decreasing frequency of number of edited events produced. The *y*-axis displays the breakdown in number of edited events detected for each line for the top 5 most abundant edits detected at Bm3-2070, colored by specific edit type. Note there were two distinct − 8 bp edits occurring at high frequency at this target site. **B** On the *x*-axis are each of the soybean lines, clustered by RM. The *y*-axis displays the breakdown in number of edited events detected for each line for the top 5 most abundant edits detected at Dt1-1389, colored by specific edit type. Note there were two distinct − 7 bp edits occurring at high frequency at this target site. The complete order of lines listed on the *x*-axis is presented in Supplemental Table S1

The frequency of edits produced by crRNAs in maize and soybean varied from 7 to 92% by target site (Table 1). Target site dependency in editing efficiency is consistent with previous reports of Cas12a-mediated editing in plants (Bernabé‐Orts et al. 2019). In soybean, the number of distinct edits ranged from 80 to 493 and in maize from 11 to 60. In most instances, the editing outcomes were detected in just a single line, with proportionately fewer editing outcomes detected across an increasing number of lines (Table 1). However, there were several examples where the same edit was created across multiple different soybean or maize lines (Table 1). For example, a distinct 3 bp deletion was detected in 11 out of 17 edited female maize lines at the Bm3-2070 site (Fig. 6A). In soybean, a distinct 7-bp deletion at the soybean Dt-1389 site was detected in 678 out of 1506 edited events (45%) across 98 different lines (Fig. 6B). In a separate soybean example, a distinct 5-bp deletion at the Dt-1570 site was detected in 397 out of 1506 edited events (26%) across 93 different lines. However, most soybean Dt-1389 and Dt-1570 target site edits were detected in just one to five events (median 1, average 5.34) spread across one to three lines (median 1, average 3.34). Editing was less frequent at the soybean Dt-1237 site, and most edits were detected in just one event (median 1, average 1.65) and one line (median 1, average 1.63). Given that a few editing outcomes were repeatedly detected, we hypothesize that Cas12a-induced DNA repair could be biased toward a subset of sequence structures, for example, microhomologies. Cas9 has been demonstrated to produce non-random repair outcomes that are predictable using modeling approaches (Allen et al. 2019; Leenay et al. 2019; Liu et al. 2022). We suppose that additional data would be needed to develop models that could accurately predict Cas12a editing outcomes in maize and soybean plant cells. Pre-existing crRNA target site polymorphisms can be ruled out as the cause of the observed variable editing frequency: crRNA target sites were 100% conserved among all maize or soybean germplasm included in the mixes.

**Table 1:** Generation of unique edits by crRNAs in transformation and editing of mixed soybean and maize lines (TREDMIL)

# of lines in which the same distinct edit is observed	# of distinct edits (Gm Dt1-1237 site)	# of distinct edits (Gm Dt-1389 site)	# of distinct edits (Gm Dt1-1570 site)	# of distinct edits (Zm Bm3-3279 site)	# of distinct edits (Zm Bm3-2691 site)	# of distinct edits (Zm Bm3-2070 site)
1	61	321	175	7	19	40
2–5	15	120	66	2	5	15
6–10	4	26	5	0	2	2
11–20	0	12	5	0	0	2
21–50	0	9	4	0	0	1
51–104	0	5	3	0	0	0
crRNA cutting frequency (%)^b^	7	92	59	7	25	59
Total distinct edits by site	80	493	258	11^a^	29^a^	60
Unique lines represented	63	101	98	7	13	15

^a^Includes a few edits that were present in samples that did not map to a known genotype in the seed bulk

^b^crRNA cutting frequency was calculated on all events that returned amplicon sequencing data

## Discussion

The TREDMIL concept shows the possibility to perform genotype-flexible regeneration and genome editing of 10–100 s of elite germplasm via seed embryo explant-based meristem transformation in soybean and maize. With this scale of editing, we created over 800 distinct editing outcomes at the Dt1 locus in soybean, and 95 distinct edits in the maize gene Bm3. Creation of identical edits in different germplasm can provide a mechanism with which to assess germplasm-by-edit interactions earlier in a breeding program, which would enable more rapid decision making on the broad-spectrum efficacy of a given edit. At the same time, the large spectrum of edits produced across germplasm enables genomic discoveries at a much faster pace than analysis of those same edits in just a single germplasm. In addition, the creation of many types of edits across germplasm will be useful in developing training data and models to predict how genome editing machinery will operate across different plant genomes. This information will be valuable not only to optimize existing genome editing systems, transformation resources, and edit testing strategies, but also to inform parameters needed to design new editing systems for plant cells.

Resolution of double strand DNA breaks created in plant genomes by guided nuclease cleavage events most often occurs through non-homologous end joining (NHEJ) mechanisms (Shen et al. 2017). Due to the processive, exonucleolytic variability inherent in DNA end processing prior to ligation, the size of deletion in resolved editing outcomes is somewhat random (Puchta 2004). However, our data indicate that certain editing outcomes are significantly more likely. Editing outcomes that occur more frequently at a given crRNA target site may be the result of sequence features like microhomology (Grajcarek et al. 2019). Even with simple editing methodologies that create a set of site-specific lesions resolved through NHEJ, the potential to achieve identical outcomes across multiple germplasms offers the potential to deploy and test comparable edited mutations in many isogenic crop backgrounds. Although we currently cannot accurately predict which lesions will be more frequent for a given crRNA target and Cas12a editing nuclease in maize and soybean cells, the ability to do so would add to the advantages provided by TREDMIL for deployment of edited alleles into breeding programs. TREDMIL provides a method to produce many edits in many types of germplasm whereby such information could be used in modeling approaches to better understand editing outcomes of Cas12a in plant cell systems.

These results show the TREDMIL method can produce an array of edited genetic variation across many different soybean genotypes without substantial bias in transformation or editing frequencies. A comparison of transformation and editing frequencies across the soybean maturity groups represented in the original seed mix also shows no significant bias in transformation or editing frequencies by maturity group. We recognize that these results must be taken into context within the scale of this large, segmented, experiment and that independent transformation experiments will enable further validation of these observations in soybean.

Practically, TREDMIL creates efficiencies across the spectrum from small- to large-scale transformation programs by reducing the amount of tracking, handling, and transferring steps in a transformation protocol. Line-by-line transformation would require maintaining the identity of each genotype throughout the process, likely necessitating genotyping validation at the end. Our estimate shows that TREDMIL can gain at least 25% in efficiency compared to line-by-line transformation by enabling large-scale transformation rather than many small, independent explant extraction and transformation experiments. Further, using a pooled transformation approach reduces the effects of experiment-to-experiment variation, allowing researchers to more accurately assess each germplasm’s response under the same conditions. The cost of genotyping is only 3–10% of the cost of event production for corn and soybean. With continuing reduction in genotyping costs and with greater computational efficiency, large-scale genotyping and deconvolution of germplasm identities is now practical and can be done in parallel with other molecular analyses such as copy number and sequencing assays. However, TREDMIL might be less amenable in systems or projects where there is greater value to separating germplasm upfront or if the user has limited genotyping resources to deconvolute samples from a transformed pool. Another consideration with the TREDMIL approach will be an assessment of the conservation of the target sites in the germplasm pool, if the goal is to create edits in as many lines as possible. This limitation is not inherent to the TREDMIL approach and is a consideration for any sequence-guided editing strategy used with any editing delivery system.

Simultaneous deployment of an individual allele to many representative crop varieties is challenging. Moreover, our ability to do this becomes critically important in cases where allele transfer through traditional breeding solutions is not tractable due to the timescales involved, for example, with woody crops (Brower-Toland et al. 2023). The use of gene editing to directly introduce the same or similar mutations into multiple varieties at the same time may facilitate timely introduction of useful traits into more challenging crop types with long generation times that are typically propagated through cuttings and grafts, such as citrus. This approach can also increase the throughput and efficiency of trait introduction for any crop of interest.

The simultaneous deployment of many independently segregating genome edits and transgenes is even more challenging. To overcome this hurdle, biotechnology and breeding pipelines have been integrated later in the research and development cycle through deployment strategies that rely on backcrossing or forward-breeding of specific “events.” This strategy dramatically limits the number of traits that can be deployed at one time. The strategy herein described can be used in combination with other modern breeding tools, such as the use of doubled haploids and genomic selection, to enable accelerated trait deployment. Transformation and editing efficiency will increase with protocol improvements and selection with breeding populations during this process. TREDMIL opens the door to simultaneous deployment and selection of variation, native or otherwise, already present but not yet fixed in the population. Mixes can be composed of segregating haploid or diploid progeny, instead of inbred lines, and can be subjected to marker assisted selection and identification. Mixing germplasm can also enable the rapid deployment of edits or transgenes that confer resistance to emerging biotic threats, where the reduced exposure of resistance factors would align with integrated pest management strategies. The potential for rapid late-stage trait deployment also facilitates timely retirement of edits or transgene in comparison with current timelines for forward breeding or trait integration by backcrossing.

## Methods

### Plant materials

Five thousand seeds from each of 104 elite soybean lines ranging in maturity from group 00 to group VII were mixed before excision of embryo explants. For maize mix 1, seeds from 78 elite proprietary inbred lines of temperate maize ranging from 89 to 119 days in relative maturity rating and 500 F1s were mixed before excision of embryo explants. Forty out of 78 elite lines were from Bayer Female (Stiff-Stalk) heterotic group and remaining 38 of Bayer Male (Non-Stiff-Stalk) heterotic group. Out of 500 F1s, 250 F1s were from male-by-male crosses and remaining 250 were from female-by-female crosses. All inbred parents of these F1s were among the 78 inbreds included in the mixture. Seed quantities varied from 1000 to 9000 per inbred, and 22,000 female and male F1 were included in the mix for excision of seed embryo explants. For maize male mix 2, a total of 36 maize male lines from RM 110–112 were grown under the same condition. Seeds ranging from 2000 to 26,000 from each line were mixed before seed embryo explant excision.

### Agrobacterium strains, plasmids, and Agrobacterium preparation

Agrobacterium strain AB30 and AB32 (Ye et al. 2016) were used for transformation of soybean and maize, respectively. In this study, the binary vector for soybean transformation, pM206, comprised of three cassettes between the T-DNA borders, including an *aadA* selectable marker cassette for selecting transformants, a Cas12a cassette and a crRNA cassette for performing genome editing at the target site of the gene of interest *Dt-1*. The binary vector construct pM795 for maize mix 1 transformation containing *cp4* selectable marker for glyphosate selection, a Cas12a cassette and a crRNA cassette for genome editing target site of the gene of interest *BM3*. The binary vector for maize male mix 2 transformation was pM138 with *uidA* as a gene of interest and *cp4* as the selectable marker. Schematic representation of plasmid pM206, pM795 and their respective crRNA sequences are shown in Supplementary Figs. S1 and S2. The schematic representation of pM138 is shown in Supplementary Fig. S3.

For *Agrobacterium* preparation, an aliquot of 250 µL of an *Agrobacterium* glycerol stock solution was inoculated into 250 mL of LB medium with the appropriate antibiotics. The mixture was cultured with shaking at 200 rpm at 28 °C for 16–18 h or until OD_660_ reached 0.6–1.2. After the *Agrobacterium* cells were pelleted at 3500 RCF for 25 min at 4 °C and the supernatant was discarded, For soybean, the *Agrobacterium* cells were resuspended in inoculation medium (2/5 of B5 macro salts, 1/10 of B5 micro salts and vitamins, 1 g/L potassium nitrate, 30 g/L glucose, 3.9 g/L 2-(N-morpholino) ethanesulfonic acid (MES) supplemented with 52 mg/L lipoic acid, and 1 mg/L thidiazuron-TDZ pH 5.5) to a final OD_660_ reading of 0.35. For maize, the *Agrobacterium* cells were resuspended in inoculation medium (2/5 of B5 macro salts, 1/10 of B5 micro salts and vitamins, 1 g/L potassium nitrate, 30 g/L glucose, 3.9 g/L MES, pH 5.5) to a final OD_660_ of 0.25.

### Soybean transformation

Soybean transformation was carried out as described (Ye et al. 2008, 2011) with modifications based on the improvement described previously (Chen et al. 2014). Briefly, soybean seed embryo explants comprising regenerable tissue including embryonic and/or meristematic tissue were milled and excised from the mixed seeds. The explants were purified through removal of debris and unnecessary seed parts and were rehydrated in rehydration medium (200 g/L polyethylene glycol (PEG), 2 mL antilife fungicide with 85% daconil), on a shaker at 70 rpm for about 1 h and rinsed thoroughly with sterile water. Rehydrated and rinsed explants were inoculated in 50 mL of the *Agrobacterium* preparation/Plantcon and sonicated at 45 kHz for 20 s. After sonication, soybean explants in *Agrobacterium* preparation were placed on a shaker at 80 rpm for 30 min. After the inoculation, the *Agrobacterium* solution was removed through aspiration. Explants were then transferred to co-culture plates with a piece of sterile filter paper wetted with 2 mL of co-culture medium (2/5 of B5 macro salts, 1/10 of B5 micro salts and vitamins, 1 g/L potassium nitrate, 30 g/L glucose, 3.9 g/L MES supplemented with 52 mg/L lipoic acid, and 1 mg/L TDZ, 10 mg/L Thiabendazole (TBZ) and 50 mg/L Nystatin, pH 5.5. The co-culture plates were incubated at 23 °C for 3 to 5 days, with a photoperiod of 16-h light/8-h dark with a light intensity of 90 µmol/m^2^ s and a relative humidity of approximately 45%.

The explants were then transferred to solid selection and regeneration medium (3.21 g/L Gamborgs B5 Medium, 20 g/L sucrose, 1.29 g/L Calcium Gluconate, 0.03 g/L Clearys 3336 WP, 4 g/L Agar gel, 200 mg/L Carbenicillin, 100 mg/L Timentin, 200 mg/mL Cefotaxime, and 150 mg/L Spectinomycin) and cultured at 28 °C, with a photoperiod of 16-h light/8-h dark, and a light intensity of 120 µmol/m^2^ s. µE. Approximately 7 weeks after inoculation, regenerated soybean plants were transferred to soil plugs (Jiffy Carefree Plugs). Plantlets were maintained at 28 °C during the day and 23 °C during the night. Leaf tissues were sampled for molecular confirmation of transformation, genotyping and editing analysis at ~ 3 weeks post plugging.

### Maize transformation

Maize transformation was conducted as described (Ye et al. 2022) with the following modifications. Briefly, maize embryo explants excised from the mixed seeds were surface sterilized with 70% ethanol containing 100 g/L PEG MW 8000 (polyethylene glycol) for 4 min with agitation and then rinsed thoroughly with repeated rinses. Explants were then collected, enriched, and purified by repeated floatation in sterile water. Collected explants were transferred to rehydration medium containing (2/5 of B5 macro salts, 1/10 of B5 micro salts and vitamins, 1 g/L potassium nitrate, 30 g/L glucose, 3.9 g/L MES, 0.03 g/L Clearys 3336 WP, pH 5.5 for 1–2 h. Rehydrated explants were inoculated with *Agrobacterium* preparation and subjected to a pressure of 300 psi for 3 min and followed by centrifugation at 2619 *g* for 30 min at 4 °C. Following inoculation, the *Agrobacterium* solution was removed, and the inoculated explants were blotted on a sterile paper towel to remove excess inoculation media. Explants were then transferred to co-culture Petri plates (100 mm) with a piece of sterile Whatman #1 filter paper (82 mm) wetted with 1.25 mL of the rehydration media. The co-culture plates were incubated at 20 °C for 6 days at a photoperiod of 16-h light/8-h dark with a light intensity of 90 µmol/m^2^ s and a relative humidity of approximately 65%.

After co-culture, explants were transferred to Petri plates containing a bud induction medium containing MS salts, B5 vitamins, 30 g/L sucrose, 0.69 g/L proline, 1 g/L NZ amine-A, 2 mg/L glycine, 1 g/L MES, 1 mg/L 2, 4-D, 10 mg/L BAP, 400 mg/L carbenicillin, 200 mg/L cefotaxime, 100 mg/L timentin, 3.5 g/L low EEO agarose, pH 5.8 and cultured for 7 d at 33 °C at a photoperiod of 16-h light/8-h dark with and a light intensity of 90 µmol/m^2^ s. The explants were then transferred to Petri plates containing 2nd bud induction medium containing MS basal salts, B5 vitamins, 60 g/L sucrose, 0.5 g/L glutamine, 1 g/L NZ Amine-A, 0.69 g/L proline, 2 mg/L glycine, 1.95 g/L MES, 1.25 mg/L cupric sulfate, 2 mg/L thidiazuron, 2 mg/L picloram, 400 mg/L carbenicillin, 200 mg/L cefotaxime, 100 mg/L timentin, 25 µM glyphosate, and 3.5 g/L low EEO agarose, pH 5.8 and cultured at 28 °C for 14 d at a photoperiod of 16-h light/8-h dark with a light intensity of 150 µmol/m^2^ s. Finally, explants were transferred to regeneration medium containing LM Woody Plant Medium salts and vitamins, 0.03 g/L Clearys 3336 WP, 30 g/L sucrose, 0.69 g/L proline, 2 mg/L glycine, 1 g/L MES, 3.5 g/L low EEO agarose, 400 mg/L carbenicillin, 200 mg/L cefotaxime, 100 mg/L timentin and 20 µM glyphosate, pH 5.8 and cultured at 28 °C for 6 weeks at a photoperiod of 16-h light/8-h dark with a light intensity of 160 µmol/m^2^ s. Regenerated plants were collected and assayed for genotyping, transformation, and confirmation of gene editing from the mixed explants.

### TaqMan analysis of transgene copy number

Leaf samples of both soybean and maize plants were collected for DNA extraction as described (Dellaporta et al. 1983). Transgene copy number was determined by TaqMan method according to manufacturer’s instruction (Applied Biosystems, Froster City, CA) (Bubner and Baldwin 2004). For soybean, forward primer 5′-AGCTAAGCGCGAACTGCAAT-3′, reverse primer 5′-GGCTCGAAGATACCTGCAAGA-3′ and TaqMan probe 6FAM-TGGAGAATGGCAGCGCAATGACA against aadA selection marker were used for copy number analysis. Similarly, for corn, forward primer 5′-ACGATTTCGACAGCACCTTCA-3′, reverse primer 5′-GTCACCGTCTTCCGATTTCAC-3′ and TaqMan probe 6FAM-ACGCCTCGCTCACAAAGCGCC against epsps-cp4 selection marker were used for copy number analysis.

For soybean, transformation frequency is defined as the percentage of inoculated embryo explants producing events having selectable marker gene. For maize, transformation frequency is defined as the percentage of inoculated embryo explants producing non-chimeric R0 events having selectable marker gene. Relative transformation frequency score of each line in the mixes of both soybean and maize is the ratio of transformation frequency of each line in the population over the overall transformation frequency of the mix population.

### Genotyping analysis

For soybean, 50 TaqMan markers were selected to maximize the fingerprint diversity of the 104 soybean lines. The combination of genetic markers utilized to fingerprint individual germplasms was chosen according to three main criteria: (1) markers have high informativeness, specifically that have a high minor (rare) allele frequency within the mix, such that the presence of a minor allele in the population is present at a level high enough to allow for elimination of several germplasms from consideration for identification; (2) markers with consistent high call rate (low missing data) and high data quality; and (3) markers, which in combination, produce some redundancy in identification, such that if a sample lacks data for some marker loci, robust identification is possible based on unambiguous data from the total combination of genetic markers that did return quality data. In addition, markers were chosen to cover a wide range of SNPs on different chromosomes to increase coverage of the genome. Identification required at least 90% similarity with the best match. Material for which identity could not be ascertained because of excessive missing data was 20%.

All maize inbred parents and controls had been fingerprinted. From this prior knowledge, we selected 26 TaqMan markers with a high information content among them that would allow the identification of any material from the mixes even in the presence of some missing data. In most instances, as little as 15 returned datapoints would suffice to identify a mix component. Identification required at least 90% similarity with the best match, and at least 5% better similarity with best match than with second-best match. Material for which identity could not be ascertained because of excessive missing data was also below 5%.

### Deep sequencing analysis for identification of edited lines and plants

For amplicon sequencing analysis at the *bmr3* target site, a 2759 bp genomic region flanking the three 23 nt target sites was amplified using a pair of gene-specific forward and reverse primers (5′-GTCATGGATGGGAGCGAGTGAAC-3′ and 5′-GTGTGCGCACGTTGTGAGTGTC-3′). For amplicon sequencing analysis at the dt1 target site, a 1870 bp genomic region flanking the three 23 nt target sites was amplified using a pair of gene-specific forward and reverse primers (5′-TCCATGCTTAATCGGCATCACT-3′ and 5′-AACCTTGGGCTTGGTGTTGA-3′). Amplicons were then used for sequencing library creation using the Illumina DNA Prep Kit (Illumina, Inc., San Diego, CA) in addition to unique dual index barcodes (UDIs) from IDT (Integrated DNA Technologies, Coralville, IA) for demultiplexing in analysis. PCR amplifications were performed using Phusion Flash High-Fidelity PCR Master Mix (Themo Fisher Scientific, Inc., Waltham, MA). Amplicon pools were purified with SeqPure PCR Purification Kit (BioChain Institute Inc., Newark, CA), and sequenced on NovaSeq (Illumina, Inc., San Diego, CA) according to Illumina sequencing protocols. Trimmomatic (V0.36) was used for trimming adapter sequence and quality control of sequencing reads and aligned using a global–local alignment algorithm (glsearch). Reads with nucleotide deletions or insertions that are within or originated from the target site were defined as indels. A percent reads for each indel was calculated by taking the reads containing that indel/all reads spanning the target site, indel% = 100% × (reads with indels)/(total reads spanning the target site). Any plant having an indel with a percent read > 10% is considered an edited plant, with that edit being likely heritable. This empirical cut-off was refined based on many previous transformation and editing experiments performed under similar conditions. Operationally: above this cutoff, any sample with one edited allele and no wild type is called homozygous; any sample with one edited and one wild-type allele is called heterozygous; any sample with 2 distinct edited alleles is called biallelic; any sample with > 2 distinct alleles is called chimeric. Editing frequency is a measure of the number of regenerated plants that are edited divided by the total number of regenerated plants assayed for edits.

### Supplementary Information

Below is the link to the electronic supplementary material.

Supplementary file1 (DOCX 293 kb)

## Data Availability

The authors declare that all data supporting the findings of this study are available within the article and its supplementary information files are available from the corresponding author upon request.
