# Supplementary material for: Simultaneous genetic transformation and genome editing of mixed lines in soybean (Glycine max) and maize (Zea mays)
Source: aBIOTECH. 2024 Jun 18;5(2):169–83. doi: 10.1007/s42994-024-00173-5 (PMC11224177; doi:10.1007/s42994-024-00173-5)
Supplement: Supplementary file 1 — Supplementary file1 (DOCX 293 kb) [file 42994_2024_173_MOESM1_ESM.docx]

Simultaneous Genetic Transformation and Genome Editing of Mixed Lines in Soybean (*Glycine max*) and Maize (*Zea mays*)

Michelle Valentine, David Butruille, Frederic Achard, Steven Beach, Brent Brower-Toland, Edward Cargill, Megan Hassebrock, Jennifer Rinehart, Thomas Ream, Yurong Chen*

Bayer Crop Science

700 Chesterfield Parkway W

Chesterfield, Missouri, USA 63017

Corresponding authors

Edward Cargill

[edward.cargill@bayer.com](mailto:edward.cargill@bayer.com)

Yurong Chen <https://orcid.org/0000-0002-6791-8356>

[yurong.chen@bayer.com](mailto:yurong.chen@bayer.com)

Supplementary Figure 1. Schematic representation of T-DNA structure of pM206 and crRNA sequences for targeting Dt1 locus. (A) Selectable marker *aadA* is under control of the actin promoter while Cas12a is driven by the ubiquitin promoter. crRNAs are expressed in tandem using a U6 (Pol3) cassette; (B) crRNA target site sequences with PAM sites underlined.

**LB**: Left T-DNA border; **P-At.Act7**: *Arabidopsis* actin 7 promoter; **aadA**: aadA*-SPC/STR* gene from *Tn7* encoding streptomycin adenyltransferase; ***T*-nos**: *Agrobacterium nos* transcription terminator (Depicker et al. 1982); **P-Mt.Ubq2**: *Medicago* ubiquitin promoter; **Lb.Cas12a**: *Lachnospiraceae* bacterium Cas12a coding region; **Mt.AC**: *Medicago* transcription termination sequence from gene of unknown function; **U6i-crRNA:** crRNA cassette driven by *Glycine max* U6 promoter; **RB**: Right T-DNA border.

Supplementary Figure 2. Schematic representation of T-DNA structure of pM795 and crRNA sequences for targeting BM3 (Brown midrib 3) locus (A) Selectable marker *cp4* was under control of actin promoter while Cas12a is driven by the ubiquitin promoter. crRNAs are expressed in tandem using a U6 (Pol3) cassette; (B) crRNA target site sequences with PAM sites underlined.

**LB**: Left T-DNA border; **P-OsAct1**: rice actin 1 promoter; **CP4**: cp4 *epsps* gene from *Agrobacterium* CP4 strain encoding for 5-enolpyruvulshikimate-3-phosphate synthase; ***T*-nos**: *Agrobacterium nos* transcription terminator (Depicker et al. 1982); **P-Zm.UbqM1**: Zea mays ubiquitin promoter; **Lb.Cas12a**: *Lachnospiraceae* bacterium Cas12a coding region; **Os.LTP**: Oryza sativa lipid transfer protein transcription termination sequence; **ZmU6-crRNA:** crRNA casette driven by Zea mays U6 promoter; **RB**: Right T-DNA border.

Supplementary Figure 3. T-DNA structure of pM138. **LB**: Left T-DNA border; **P-OsAct1**: rice actin 1 promoter; ***CP4****: cp4 epsps gene from Agrobacterium* CP4 strain encoding for 5-enolpyruvulshikimate-3-phosphate synthase; T-nos: Agrobacterium nos transcription terminator (Depicker et al. 1982); **P-ERIra.Ubq**: *Tripidium ravennae* ubiquitin promoter; *uidA* gene with intron (Vancanneyt et al. 1990) **T-Sb.Ccd**: *Sorghum bicolor* cortical cell-delineating protein terminator; **RB**: Right T-DNA border.

Supplementary Table 1. The complete order of lines listed on the x-axis as presented in Figures 2A, 5B and 6B.

| **Order of Figure 2A line ID x-axis labels (from left to right)** | **Order of Figure 5B line ID x-axis labels (from left to right)** | **Order of Figure 6B line ID x-axis labels (from left to right)** |
| --- | --- | --- |
| 5 | 76 | 76 |
| 8 | 77 | 77 |
| 4 | 78 | 78 |
| 71 | 90 | 90 |
| 23 | 37 | 37 |
| 96 | 39 | 39 |
| 104 | 40 | 40 |
| 83 | 41 | 41 |
| 76 | 68 | 68 |
| 14 | 70 | 70 |
| 16 | 71 | 71 |
| 18 | 72 | 72 |
| 63 | 88 | 88 |
| 82 | 9 | 9 |
| 49 | 10 | 10 |
| 53 | 11 | 11 |
| 2 | 32 | 32 |
| 21 | 42 | 42 |
| 75 | 73 | 73 |
| 78 | 74 | 74 |
| 11 | 75 | 75 |
| 13 | 89 | 89 |
| 40 | 100 | 100 |
| 51 | 101 | 101 |
| 93 | 102 | 102 |
| 85 | 103 | 103 |
| 12 | 104 | 104 |
| 39 | 2 | 2 |
| 97 | 3 | 3 |
| 17 | 4 | 4 |
| 33 | 5 | 5 |
| 72 | 6 | 6 |
| 74 | 7 | 7 |
| 43 | 8 | 8 |
| 87 | 12 | 12 |
| 19 | 13 | 13 |
| 29 | 14 | 14 |
| 37 | 15 | 15 |
| 42 | 16 | 16 |
| 44 | 33 | 33 |
| 27 | 38 | 38 |
| 91 | 43 | 43 |
| 99 | 44 | 44 |
| 88 | 56 | 56 |
| 30 | 80 | 80 |
| 73 | 17 | 17 |
| 95 | 18 | 18 |
| 10 | 19 | 19 |
| 22 | 20 | 20 |
| 68 | 21 | 21 |
| 41 | 22 | 22 |
| 59 | 23 | 23 |
| 62 | 24 | 24 |
| 65 | 25 | 25 |
| 70 | 26 | 26 |
| 20 | 27 | 27 |
| 47 | 28 | 28 |
| 57 | 29 | 29 |
| 66 | 30 | 30 |
| 101 | 45 | 45 |
| 102 | 46 | 46 |
| 103 | 47 | 47 |
| 35 | 48 | 48 |
| 100 | 50 | 50 |
| 24 | 51 | 51 |
| 46 | 53 | 53 |
| 9 | 97 | 87 |
| 64 | 98 | 97 |
| 60 | 99 | 98 |
| 32 | 87 | 99 |
| 45 | 34 | 31 |
| 31 | 35 | 34 |
| 7 | 36 | 35 |
| 38 | 31 | 36 |
| 56 | 52 | 49 |
| 86 | 49 | 52 |
| 54 | 58 | 54 |
| 81 | 59 | 55 |
| 50 | 57 | 57 |
| 61 | 61 | 58 |
| 84 | 62 | 59 |
| 98 | 63 | 61 |
| 55 | 64 | 62 |
| 90 | 54 | 63 |
| 34 | 55 | 64 |
| 79 | 82 | 79 |
| 15 | 83 | 81 |
| 89 | 84 | 82 |
| 6 | 79 | 83 |
| 48 | 81 | 84 |
| 52 | 85 | 85 |
| 92 | 86 | 86 |
| 67 | 65 | 60 |
| 25 | 66 | 65 |
| 77 | 67 | 66 |
| 26 | 60 | 67 |
| 28 | 91 | 91 |
| 36 | 92 | 92 |
| 58 | 93 | 93 |
| 3 | 95 | 95 |
| 80 | 96 | 96 |

Supplementary Table 2A. List of editing profiles by crRNA target site for each soybean event. Any sample with one edited allele and no wild type allele is called homozygous; any sample with one edited allele and one wild type allele is called heterozygous; any sample with 2 distinct edited alleles is called biallelic; any sample with >2 distinct alleles is called chimeric. Only sequences represented by >10% frequency spanning the target site are included. yr contact in.

| **Transformation rep #** | **Event ID** | **Maturity group** | **Line ID** | **Dt1pro-1237G** | **Dt1pro-1389A** | **Dt1pro-1570C** |
| --- | --- | --- | --- | --- | --- | --- |
| 1 | 1 | II | 56 | wt | biallelic, -8/-13 | het, wt/-5 |
| 1 | 2 | VII | 95 | wt | het, wt/-7 | het, wt/-23 |
| 1 | 3 | II | 38 | wt | het, -7/wt | wt |
| 1 | 4 | IV | 54 | wt | chimeric, -11/-7/wt | het, wt/-10 |
| 1 | 5 | V | 92 | wt | chimeric, wt/-10/-6 | wt |
| 1 | 6 | 00 | 90 | wt | chimeric, -9/wt/-29 | het, wt/-16 |
| 1 | 7 | IV | 52 | wt | chimeric, -7/-11/-42 | het, wt/-5 |
| 1 | 8 | III | 22 | het, wt/-1 | chimeric, wt/-12/-7 | chimeric, wt/-12/-3 |
| 1 | 9 | III | 22 | wt | chimeric, wt/-7/-6 | chimeric, wt/-43/-5 |
| 1 | 10 | III | 28 | wt | chimeric, -40/wt/-6 | het, wt/-18 |
| 1 | 11 | III | 26 | wt | het, -11/wt | wt |
| 1 | 12 | VI | 93 | wt | het, -13/wt | het, wt/-13 |
| 1 | 13 | III | 48 | wt | chimeric, -7/wt/-29 | het, wt/-9 |
| 1 | 14 | II | 3 | wt | het, wt/-7 | wt |
| 1 | 15 | I | 101 | het, wt/s1 | biallelic, -18/-11 | het, -5/wt |
| 1 | 16 | V | 92 | chimeric, -7/wt/-13 | het, -290/wt | het, wt/-290 |
| 1 | 17 | IV | 55 | wt | het, wt/-11 | wt |
| 1 | 18 | II | 80 | wt | chimeric, -9/-7/wt | het, wt/-26 |
| 1 | 19 | III | 26 | wt | het, -12/wt | wt |
| 1 | 20 | I | 101 | wt | chimeric, -11/wt/-7 | wt |
| 1 | 21 | IV | 52 | wt | chimeric, -40/wt/-13 | wt |
| 1 | 22 | III | 99 | wt | chimeric, -295/-10/wt | chimeric, -295/-5/wt |
| 1 | 23 | III | 98 | het, wt/-6 | chimeric, -424/-6/wt | chimeric, -424/-6/wt |
| 1 | 24 | IV | 35 | wt | chimeric, -83/wt/-7 | het, -5/wt |
| 1 | 25 | V | 60 | wt | chimeric, wt/-12/-5 | chimeric, wt/-5/-4 |
| 1 | 26 | V | 67 | wt | chimeric, -6/-23/wt | chimeric, -5/wt/-102 |
| 1 | 27 | IV | 79 | wt | het, wt/-7 | wt |
| 1 | 28 | IV | 57 | wt | het, -7/wt | het, wt/-5 |
| 1 | 29 | VII | 96 | het, wt/-52 | chimeric, -31/-24/wt | chimeric, -7/-14/wt |
| 1 | 30 | II | 4 | chimeric, wt/-8/s1,-4,s1,-10 | chimeric, -85/wt/-19 | het, -5/wt |
| 1 | 31 | III | 30 | wt | wt | wt |
| 1 | 32 | II | 13 | wt | chimeric, -9/-7/wt | wt |
| 1 | 33 | III | 50 | wt | chimeric, -11/wt/-7/-6 | chimeric, wt/-5/-5/-9 |
| 1 | 34 | 0 | 68 | wt | het, wt/-7 | het, wt/-5 |
| 1 | 35 | IV | 64 | wt | chimeric, -7/wt/-17 | chimeric, wt/-9/-5 |
| 1 | 36 | 0 | 41 | wt | chimeric, -7/-16/-12 | chimeric, -6/-100/-26/-9 |
| 1 | 37 | III | 27 | het, wt/-9 | het, -17/wt | chimeric, -5/wt/-51 |
| 1 | 38 | II | 13 | wt | chimeric, wt/-7/-9 | wt |
| 1 | 39 | II | 3 | wt | chimeric, wt/-20/s1,-10 | wt |
| 1 | 40 | II | 38 | wt | chimeric, wt/s1,-7/s1,-6 | het, wt/-5 |
| 1 | 41 | III | 45 | wt | het, wt/s1,+9,s1,-22 | het, wt/-5 |
| 1 | 42 | II | 38 | wt | chimeric, -7/-22/-11 | het, -5/wt |
| 1 | 43 | IV | 36 | het, wt/-1138 | chimeric, -1138/-12/wt | chimeric, -1138/-18,s2/wt |
| 1 | 44 | III | 25 | wt | het, -7/wt | wt |
| 1 | 45 | IV | 55 | wt | chimeric, -8/wt/-6 | chimeric, wt/-5/-61 |
| 1 | 46 | III | 26 | wt | chimeric, -33/-13/wt | wt |
| 1 | 47 | II | 56 | wt | chimeric, wt/-87/-21 | wt |
| 1 | 48 | I | 89 | wt | het, wt/-9 | het, wt/-9 |
| 1 | 49 | III | 28 | wt | chimeric, -31/wt/-36 | het, wt/-5 |
| 1 | 50 | III | 97 | het, wt/-8 | chimeric, wt/-12/-8 | chimeric, wt/-16/-75 |
| 1 | 51 | IV | 58 | wt | het, wt/-184 | het, wt/-184 |
| 1 | 52 | IV | 36 | wt | chimeric, -195/-5/wt | chimeric, wt/-195/-11 |
| 1 | 53 | V | 92 | wt | chimeric, wt/-133/-7 | wt |
| 1 | 54 | IV | 52 | wt | chimeric, -7/wt/-9 | het, wt/-5 |
| 1 | 55 | IV | 64 | wt | het, -8/wt | het, wt/-5 |
| 1 | 56 | V | 91 | wt | chimeric, -9/-7/wt | chimeric, wt/-10/-6 |
| 1 | 57 | III | 26 | wt | het, wt/-7 | wt |
| 1 | 58 | 0 | 39 | het, wt/-363 | chimeric, -82/wt/-363/-13 | chimeric, wt/-363/-26 |
| 1 | 59 | III | 30 | wt | chimeric, -188/-7/wt | chimeric, -188/-119/wt |
| 1 | 60 | III | 30 | wt | chimeric, -7/-13/wt | chimeric, wt/-3/-5 |
| 1 | 61 | I | 89 | wt | het, wt/-7 | wt |
| 1 | 62 | I | 103 | wt | chimeric, -13/-7/wt | wt |
| 1 | 63 | IV | 64 | wt | chimeric, wt/-5/-7 | wt |
| 1 | 64 | II | 2 | wt | chimeric, wt/-44/-7,s2,s6,s3 | wt |
| 1 | 65 | III | 47 | wt | het, -12/wt | chimeric, -18/wt/-25 |
| 1 | 66 | 00 | 90 | wt | chimeric, wt/-67/-6 | wt |
| 1 | 67 | I | 74 | wt | chimeric, -11/-7/wt | chimeric, wt/-5/-5 |
| 1 | 68 | I | 89 | wt | chimeric, -7/wt/-9 | het, wt/-5 |
| 1 | 69 | III | 25 | wt | chimeric, -7/wt/-17 | chimeric, -51/-6/wt |
| 1 | 70 | I | 102 | het, wt/-390 | chimeric, -390/-5,s1,s1,-15/wt | chimeric, -5/-27/wt |
| 1 | 71 | III | 46 | wt | wt | wt |
| 1 | 72 | IV | 52 | wt | chimeric, -82/-12/wt | wt |
| 1 | 73 | IV | 79 | wt | chimeric, wt/-9/-7 | chimeric, wt/-10/-20 |
| 1 | 74 | I | 103 | wt | chimeric, -23/-6/wt | het, wt/-5 |
| 1 | 75 | II | 80 | wt | chimeric, wt/-9/-6 | chimeric, wt/-34/-29 |
| 1 | 76 | 0 | 39 | wt | chimeric, -13/wt/-7 | wt |
| 1 | 77 | II | 3 | wt | chimeric, -185/-27/wt | het, wt/-185 |
| 1 | 78 | I | 100 | wt | chimeric, -7/-10/wt | het, wt/-5 |
| 1 | 79 | V | 67 | wt | het, wt/-7 | wt |
| 1 | 80 | III | 98 | wt | chimeric, wt/-81/-6 | het, wt/-5 |
| 1 | 81 | I | 73 | het, wt/-6 | chimeric, wt/-12/-22 | chimeric, wt/-5/-10 |
| 1 | 82 | III | 24 | wt | chimeric, -30/wt/s1,-8 | het, wt/-9 |
| 1 | 83 | 00 | 90 | het, wt/-13 | het, -7/wt | chimeric, -101/-5/wt |
| 1 | 84 | III | 25 | wt | het, wt/-7 | wt |
| 1 | 85 | II | 44 | wt | chimeric, -20/-7/wt | het, wt/-5 |
| 1 | 86 | III | 98 | wt | het, wt/-7 | wt |
| 1 | 87 | I | 42 | wt | chimeric, -7/-8/wt | het, wt/-5 |
| 1 | 88 | IV | 84 | wt | chimeric, -105/wt/-7 | chimeric, -5/wt/-5 |
| 1 | 89 | IV | 62 | wt | wt | wt |
| 1 | 90 | III | 48 | wt | het, -7/wt | het, -14/wt |
| 1 | 91 | III | 45 | wt | chimeric, -11/-18/wt | het, wt/-51 |
| 1 | 92 | IV | 79 | wt | chimeric, -8/-5,+14/wt | het, -6/wt |
| 1 | 93 | III | 47 | chimeric, wt/-378/-163 | chimeric, -378/-163/wt | chimeric, wt/-378/-7 |
| 1 | 94 | V | 60 | wt | chimeric, -39/-22/-14/s1,-12 | chimeric, wt/-10/-5 |
| 1 | 95 | II | 38 | wt | chimeric, -57/-7/wt | het, wt/-39 |
| 1 | 96 | IV | 84 | wt | chimeric, -7/-8/-11 | chimeric, -6/-5/-48 |
| 1 | 97 | IV | 79 | het, wt/-5 | chimeric, -18/-7/wt | het, wt/-3 |
| 1 | 98 | III | 28 | wt | chimeric, wt/-8/-7 | wt |
| 1 | 99 | 0 | 68 | wt | chimeric, -27/-9/wt | wt |
| 1 | 100 | V | 91 | wt | chimeric, -6/-6/wt | chimeric, -2/wt/-51 |
| 1 | 101 | III | 24 | wt | het, wt/-7 | wt |
| 1 | 102 | 0 | 88 | wt | het, wt/-7 | wt |
| 1 | 103 | III | 26 | wt | chimeric, wt/-102/-16 | wt |
| 1 | 104 | III | 48 | wt | het, wt/-6 | wt |
| 1 | 105 | V | 92 | wt | chimeric, -8/-7/wt | het, wt/-10 |
| 1 | 106 | 0 | 68 | wt | chimeric, -138/wt/-7 | wt |
| 1 | 107 | IV | 85 | wt | het, wt/-7 | het, wt/-63 |
| 1 | 108 | II | 33 | wt | chimeric, -14/-7/wt | chimeric, -5/-19/wt |
| 1 | 109 | II | 6 | wt | het, -6/wt | chimeric, wt/+3/+3 |
| 1 | 110 | IV | 61 | wt | chimeric, -16/-7/wt | chimeric, -5/wt/-5 |
| 1 | 111 | IV | 31 | wt | wt | wt |
| 1 | 112 | IV | 34 | wt | het, wt/-7 | het, wt/-5 |
| 1 | 113 | IV | 31 | wt | chimeric, wt/-7/-10 | chimeric, wt/-6/-5 |
| 1 | 114 | III | 26 | wt | chimeric, wt/s1,-11/-7 | het, wt/-3 |
| 1 | 115 | II | 3 | wt | chimeric, -36/-20/wt | chimeric, -5/wt/-9 |
| 1 | 116 | IV | 64 | wt | biallelic, -8/-7 | wt |
| 1 | 117 | III | 50 | wt | het, -13/wt | het, -5/wt |
| 1 | 118 | III | 25 | wt | het, wt/-16 | wt |
| 1 | 119 | III | 97 | wt | chimeric, -39/-7/-65 | het, wt/-5 |
| 1 | 120 | V | 66 | het, wt/-1362 | chimeric, wt/-1362/-7 | chimeric, wt/-1362/-11 |
| 1 | 121 | II | 80 | wt | chimeric, -32/-271/wt | het, wt/-271 |
| 1 | 122 | V | 60 | wt | het, wt/-7 | wt |
| 1 | 123 | V | 92 | wt | chimeric, wt/-9/-16 | wt |
| 1 | 124 | III | 45 | wt | chimeric, -13/-7/wt | wt |
| 1 | 125 | IV | 79 | wt | wt | wt |
| 1 | 126 | IV | 52 | het, wt/-14 | chimeric, -11/-13/-7/-7 | chimeric, -5/-20/-21 |
| 1 | 127 | V | 92 | het, wt/-8 | chimeric, -211/-6/wt | chimeric, -26/-211/wt |
| 1 | 128 | II | 6 | wt | chimeric, -126/s1,-10/wt | wt |
| 1 | 129 | 00 | 90 | wt | chimeric, -25/wt/-7 | wt |
| 1 | 130 | 0 | 70 | wt | het, wt/-6 | het, wt/-5 |
| 1 | 131 | IV | 31 | wt | chimeric, -591/-6/wt | chimeric, -591/wt/-31 |
| 1 | 132 | IV | 58 | wt | chimeric, wt/-31/s1,-9 | het, wt/-5 |
| 1 | 133 | 0 | 41 | wt | chimeric, s4,s3/-11/-186/-7 | chimeric, -186/-5/wt/-10 |
| 1 | 134 | III | 87 | wt | chimeric, wt/-7/-31 | wt |
| 1 | 135 | I | 73 | wt | chimeric, -140/-24/wt | wt |
| 1 | 136 | III | 51 | wt | het, -22/wt | wt |
| 1 | 137 | IV | 52 | wt | chimeric, wt/-9/s3,-6 | chimeric, wt/-5/-3 |
| 1 | 138 | II | 7 | wt | chimeric, wt/-17/-155 | het, wt/-5 |
| 1 | 139 | II | 6 | wt | chimeric, -7/-19/wt | chimeric, -6/-9/wt |
| 1 | 140 | 00 | 90 | wt | wt | wt |
| 1 | 141 | III | 22 | wt | chimeric, -7/wt/-10 | het, -5/wt |
| 1 | 142 | II | 80 | wt | chimeric, -9/wt/-7/-28 | het, wt/-53 |
| 1 | 143 | V | 67 | wt | chimeric, wt/-11/-7 | chimeric, wt/-5/-18 |
| 1 | 144 | III | 25 | wt | chimeric, wt/-52/-17 | het, wt/-10 |
| 1 | 145 | II | 80 | wt | chimeric, -10/wt/-6 | het, wt/-4 |
| 1 | 146 | III | 99 | wt | chimeric, wt/-11/-7 | het, wt/-5 |
| 1 | 147 | III | 27 | wt | chimeric, wt/-14/-7 | wt |
| 1 | 148 | III | 21 | wt | chimeric, wt/-13/-7 | het, wt/-5 |
| 1 | 149 | I | 9 | wt | chimeric, wt/-7/-23 | het, wt/-5 |
| 1 | 150 | 0 | 71 | wt | het, -37/wt | wt |
| 1 | 151 | 0 | 37 | wt | chimeric, -9/-19/wt | chimeric, -67/-3/wt |
| 1 | 152 | II | 33 | wt | chimeric, -7/-13/wt | chimeric, -1/-3/wt |
| 1 | 153 | II | 2 | wt | chimeric, -8/-19/wt | wt |
| 1 | 154 | IV | 82 | wt | chimeric, wt/-14/-6 | wt |
| 1 | 155 | II | 6 | wt | chimeric, -10/-6/wt | het, wt/-6 |
| 1 | 156 | III | 48 | wt | chimeric, -18/wt/-15 | wt |
| 1 | 157 | 0 | 37 | wt | chimeric, -56/wt/-7 | chimeric, wt/-3/-42 |
| 1 | 158 | II | 38 | wt | chimeric, -13/-7/wt | chimeric, -6/-10/wt |
| 1 | 159 | I | 103 | wt | het, wt/-19 | wt |
| 1 | 160 | III | 26 | het, wt/-5 | chimeric, wt/-11/-6 | chimeric, wt/-5/-52 |
| 1 | 161 | II | 15 | wt | wt | wt |
| 1 | 162 | III | 87 | wt | chimeric, -11/-7/wt | chimeric, -6/-5/wt |
| 1 | 163 | 00 | 77 | wt | chimeric, wt/-7/-14/-9/-2 | wt |
| 1 | 164 | II | 80 | wt | chimeric, -7/wt/-6 | het, -9/wt |
| 1 | 165 | I | 32 | wt | chimeric, wt/-140/-7 | wt |
| 1 | 166 | II | 3 | wt | het, wt/-7 | wt |
| 1 | 167 | II | 80 | wt | wt | wt |
| 1 | 168 | IV | 59 | wt | wt | wt |
| 1 | 169 | V | 92 | wt | chimeric, wt/-5/-8/-7 | het, wt/-3 |
| 1 | 170 | VI | 93 | wt | chimeric, -165/wt/-7 | het, wt/-5 |
| 1 | 171 | 0 | 41 | wt | chimeric, wt/-9/-186 | chimeric, wt/-186/-6 |
| 1 | 172 | III | 46 | wt | chimeric, -67/-11/wt | wt |
| 1 | 173 | III | 25 | wt | het, -7/wt | het, wt/-5 |
| 1 | 174 | IV | 61 | wt | chimeric, wt/-10/-13 | het, wt/-23 |
| 1 | 175 | IV | 54 | wt | het, wt/-7 | wt |
| 1 | 176 | 0 | 41 | wt | het, wt/-11 | wt |
| 1 | 177 | IV | 52 | wt | het, -8/wt | wt |
| 1 | 178 | IV | 34 | wt | chimeric, wt/-9/-6 | het, wt/-5 |
| 1 | 179 | V | 60 | wt | het, wt/-10 | het, wt/-5 |
| 1 | 180 | II | 6 | wt | chimeric, wt/-7/-6 | chimeric, wt/-3/-26 |
| 1 | 181 | 00 | 77 | wt | chimeric, wt/s1,-7/-7 | het, wt/-20 |
| 1 | 182 | III | 26 | wt | chimeric, -52/wt/-7 | het, wt/-5 |
| 1 | 183 | III | 46 | wt | chimeric, -187/-13/-12 | chimeric, -187/-20/wt |
| 1 | 184 | V | 67 | wt | chimeric, wt/-16/-7 | wt |
| 1 | 185 | IV | 49 | wt | chimeric, wt/-13/-17 | wt |
| 1 | 186 | 0 | 88 | wt | chimeric, wt/-7/-7 | het, wt/-6 |
| 1 | 187 | IV | 31 | wt | chimeric, wt/-16/-11 | chimeric, wt/-6/-9 |
| 1 | 188 | III | 20 | het, wt/-6 | homo, -9 | biallelic, -4/-15 |
| 1 | 189 | IV | 52 | wt | biallelic, -8/-7 | wt |
| 1 | 190 | 00 | 77 | wt | chimeric, wt/-16/-7/-7 | wt |
| 1 | 191 | III | 19 | wt | chimeric, s1,-39/wt/-10 | wt |
| 1 | 192 | III | 97 | wt | chimeric, -7/wt/-10 | wt |
| 1 | 193 | 00 | 77 | wt | chimeric, wt/-134/-6 | chimeric, wt/-5/-18 |
| 1 | 194 | III | 87 | wt | chimeric, -6/wt/-15 | het, wt/-27 |
| 1 | 195 | VII | 95 | wt | chimeric, -26/-16/wt | het, wt/-5 |
| 1 | 196 | IV | 55 | wt | chimeric, -3/wt/-6 | het, wt/-6 |
| 1 | 197 | III | 51 | wt | chimeric, -56/-7/wt | chimeric, wt/-9/-111/-50 |
| 1 | 198 | IV | 54 | wt | chimeric, -185/-8/wt | het, wt/-185 |
| 1 | 199 | V | 67 | wt | wt | wt |
| 1 | 200 | III | 48 | wt | chimeric, -8/-7/wt | chimeric, -5/wt/-21 |
| 1 | 201 | I | 101 | wt | het, wt/-9 | het, wt/-5 |
| 1 | 202 | II | 80 | wt | chimeric, wt/-10/-7 | chimeric, wt/-15/-4 |
| 1 | 203 | IV | 64 | wt | chimeric, wt/-7/-10 | chimeric, wt/-5/-21 |
| 1 | 204 | I | 32 | wt | het, -37/wt | het, wt/-5 |
| 1 | 205 | III | 46 | wt | chimeric, -9/-10/wt | chimeric, wt/-5/-3/-9 |
| 1 | 206 | IV | 64 | wt | chimeric, -86/-7/wt | chimeric, wt/-5/-42 |
| 1 | 207 | IV | 57 | wt | het, wt/+1,-3 | het, wt/-5 |
| 1 | 208 | V | 67 | wt | chimeric, -23/wt/-7 | het, wt/-5 |
| 1 | 209 | III | 30 | wt | chimeric, wt/-7/-9 | wt |
| 1 | 210 | IV | 79 | wt | chimeric, -110/wt/-13 | het, wt/-5 |
| 1 | 211 | II | 13 | wt | het, -7/wt | chimeric, wt/-5/-5 |
| 1 | 212 | I | 102 | wt | chimeric, -18/wt/-8 | wt |
| 1 | 213 | IV | 61 | wt | chimeric, -12/-20/wt | het, wt/-5 |
| 1 | 214 | II | 56 | wt | chimeric, -6/-7/wt | het, -11/wt |
| 1 | 215 | IV | 55 | wt | chimeric, wt/-7/-41 | het, wt/-34 |
| 1 | 216 | IV | 54 | wt | chimeric, wt/-114/-13 | het, wt/-6 |
| 1 | 217 | I | 9 | wt | chimeric, s1,-247/-6/wt | het, wt/s1,-247 |
| 1 | 218 | II | 15 | wt | het, wt/-7 | wt |
| 1 | 219 | I | 101 | wt | chimeric, wt/-10/-7 | het, wt/-3 |
| 1 | 220 | I | 73 | wt | chimeric, -11/wt/-6 | wt |
| 1 | 221 | II | 6 | wt | het, wt/-7 | wt |
| 1 | 222 | II | 44 | wt | chimeric, -40/wt/-6 | het, wt/-5 |
| 1 | 223 | II | 7 | wt | chimeric, wt/-13/-12 | chimeric, wt/-9/-9 |
| 1 | 224 | II | 16 | wt | het, wt/-5 | wt |
| 1 | 225 | II | 56 | het, wt/-786 | chimeric, -786/wt/-6 | het, wt/-10 |
| 1 | 226 | IV | 84 | wt | chimeric, wt/-11/-6 | chimeric, wt/-29/-6 |
| 1 | 227 | I | 32 | wt | het, wt/-7 | wt |
| 1 | 228 | II | 6 | wt | chimeric, wt/-7/-12 | chimeric, wt/-5/-3 |
| 1 | 229 | III | 24 | het, wt/-11 | chimeric, -18/-10/wt | het, wt/-44 |
| 1 | 230 | V | 66 | wt | chimeric, -26/wt/-7 | wt |
| 1 | 231 | IV | 85 | wt | chimeric, -7/wt/-6 | het, wt/-5 |
| 1 | 232 | I | 100 | het, wt/-3 | chimeric, -7/-9/wt | chimeric, -3/wt/-5 |
| 1 | 233 | IV | 79 | wt | chimeric, wt/-64/-10 | het, wt/-5 |
| 1 | 234 | I | 73 | wt | het, -7/wt | chimeric, -60/wt/-40 |
| 1 | 235 | I | 10 | wt | het, -30/wt | wt |
| 1 | 236 | 00 | 76 | wt | chimeric, wt/-11/-6 | wt |
| 1 | 237 | II | 15 | wt | het, -6/wt | het, wt/-5 |
| 1 | 238 | II | 3 | wt | het, wt/-7 | wt |
| 1 | 239 | I | 42 | wt | het, wt/-7 | wt |
| 1 | 240 | III | 53 | wt | chimeric, -38/-7/wt | het, wt/-5 |
| 1 | 241 | IV | 81 | wt | chimeric, s1,-18/-7/wt | wt |
| 1 | 242 | V | 67 | wt | chimeric, wt/-16/-4/-13 | het, wt/-5 |
| 1 | 243 | I | 42 | wt | het, wt/-18 | het, wt/-5 |
| 1 | 244 | III | 28 | wt | chimeric, -16/-7/wt | chimeric, -14/-9/wt |
| 1 | 245 | I | 73 | wt | het, wt/-7 | wt |
| 1 | 246 | II | 6 | wt | chimeric, -9/wt/-129 | chimeric, -5/wt/-21 |
| 1 | 247 | V | 91 | wt | het, wt/-7 | wt |
| 1 | 248 | IV | 36 | wt | chimeric, -27/-8/wt | het, -5/wt |
| 1 | 249 | III | 45 | wt | het, wt/-8 | wt |
| 1 | 250 | IV | 52 | wt | het, -11/wt | chimeric, wt/-6/-114 |
| 1 | 251 | II | 6 | wt | het, -10/wt | wt |
| 1 | 252 | IV | 81 | wt | chimeric, wt/-29/-18 | chimeric, wt/-5/-13/-5 |
| 1 | 253 | IV | 35 | wt | chimeric, -38/wt/-16 | wt |
| 1 | 254 | III | 51 | wt | chimeric, -64/-11/-27 | chimeric, -7/-5/wt |
| 1 | 255 | IV | 36 | wt | wt | wt |
| 1 | 256 | 00 | 90 | het, wt/-12 | chimeric, -6/wt/-29 | het, -5/wt |
| 1 | 257 | III | 99 | wt | chimeric, -14/-6/wt | het, wt/-5 |
| 1 | 258 | III | 45 | wt | het, wt/-6 | het, wt/-5 |
| 1 | 259 | I | 9 | wt | chimeric, wt/-16/-7 | het, wt/-39 |
| 1 | 260 | V | 65 | wt | chimeric, wt/-19/-20 | het, wt/-84 |
| 1 | 261 | III | 17 | wt | wt | wt |
| 1 | 262 | IV | 61 | wt | chimeric, -12/-13/wt | het, wt/-83 |
| 1 | 263 | III | 98 | wt | chimeric, -6/-9/-25/-7 | biallelic, -5/-20 |
| 1 | 264 | III | 25 | wt | het, wt/-7 | het, wt/-4 |
| 1 | 265 | I | 104 | wt | chimeric, wt/-9/-7 | wt |
| 1 | 266 | II | 2 | wt | chimeric, -46/wt/-19 | het, wt/-12 |
| 1 | 267 | I | 42 | wt | chimeric, -13/-7/wt | het, wt/-16 |
| 1 | 268 | III | 27 | het, wt/-7 | chimeric, -11/-6/wt | chimeric, -87/-9/wt |
| 1 | 269 | II | 56 | wt | chimeric, -27/wt/-7 | chimeric, wt/-5/-5 |
| 1 | 270 | I | 103 | het, wt/-2 | chimeric, -8/-6/wt | chimeric, -10/-5/wt |
| 1 | 271 | 0 | 41 | wt | chimeric, wt/-16/-7 | wt |
| 1 | 272 | I | 11 | wt | chimeric, -10/-7/wt | wt |
| 1 | 273 | IV | 64 | wt | het, wt/-11 | wt |
| 1 | 274 | II | 80 | wt | chimeric, -16/wt/-7 | wt |
| 1 | 275 | 00 | 90 | wt | chimeric, -6/-11/wt | het, wt/-6 |
| 1 | 276 | IV | 49 | wt | chimeric, -12/-7/wt | chimeric, -5/wt/-5 |
| 1 | 277 | 00 | 77 | wt | chimeric, wt/-8/-7 | het, wt/-12 |
| 1 | 278 | III | 27 | wt | chimeric, -166/wt/-139 | wt |
| 1 | 279 | 0 | 88 | wt | chimeric, wt/-6/-4 | wt |
| 1 | 280 | VII | 95 | wt | chimeric, -64/wt/-7 | het, wt/-5 |
| 1 | 281 | III | 25 | wt | chimeric, -237/-105/wt | chimeric, -5/-237/wt |
| 1 | 282 | IV | 55 | wt | chimeric, wt/-56/-7 | chimeric, wt/-9/-6 |
| 1 | 283 | 0 | 70 | wt | chimeric, wt/-7/-53 | wt |
| 1 | 284 | III | 97 | wt | chimeric, wt/s5,s1,-24/-8 | het, -5/wt |
| 1 | 285 | IV | 34 | wt | chimeric, -165/wt/-11 | chimeric, wt/-5/-5 |
| 1 | 286 | III | 45 | wt | chimeric, -11/-9/wt | wt |
| 1 | 287 | III | 24 | wt | chimeric, wt/-12/-7 | wt |
| 1 | 288 | IV | 59 | wt | chimeric, wt/-7/-5 | wt |
| 1 | 289 | IV | 34 | wt | chimeric, wt/-7/-7 | chimeric, wt/-305/-28 |
| 1 | 290 | III | 28 | wt | chimeric, -9/-8/wt | wt |
| 1 | 291 | IV | 82 | wt | wt | wt |
| 1 | 292 | IV | 79 | wt | chimeric, wt/s2,-6,s1/-7 | wt |
| 1 | 293 | I | 89 | het, wt/-334 | chimeric, -334/-143/wt | het, wt/-334 |
| 1 | 294 | IV | 55 | het, wt/-2 | chimeric, wt/-7/-10 | chimeric, wt/-25/-26 |
| 1 | 295 | I | 102 | wt | het, wt/-20 | het, wt/-5 |
| 1 | 296 | IV | 64 | wt | het, -6/wt | wt |
| 1 | 297 | II | 3 | wt | chimeric, -20/-10/wt | het, -5/wt |
| 1 | 298 | IV | 81 | wt | chimeric, -18/-24/-11/-16 | wt |
| 1 | 299 | IV | 61 | wt | chimeric, wt/-7/-6 | wt |
| 1 | 300 | III | 45 | wt | chimeric, -7/-7/wt | wt |
| 1 | 301 | III | 45 | wt | chimeric, -8/-7/wt | het, wt/-5 |
| 1 | 302 | V | 60 | wt | chimeric, wt/-11/-50/-10 | het, wt/-23 |
| 1 | 303 | IV | 86 | wt | wt | wt |
| 1 | 304 | V | 91 | het, wt/-2 | het, -7/wt | chimeric, wt/-6/-135 |
| 1 | 305 | II | 56 | wt | chimeric, wt/-7/-6 | wt |
| 1 | 306 | IV | 58 | wt | chimeric, -12/-196/-7/-5 | chimeric, wt/-6/-196/-5 |
| 1 | 307 | V | 67 | wt | het, wt/-8 | wt |
| 1 | 308 | 00 | 77 | wt | het, -26/wt | het, wt/-6 |
| 1 | 309 | III | 26 | wt | het, wt/-7 | wt |
| 1 | 310 | IV | 61 | wt | chimeric, wt/-8/-18 | wt |
| 1 | 311 | IV | 31 | wt | chimeric, -16/-11/wt | chimeric, -27/-9/wt |
| 1 | 312 | II | 33 | wt | het, wt/-11 | het, wt/-5 |
| 1 | 313 | 00 | 77 | wt | het, -7/wt | wt |
| 1 | 314 | IV | 36 | wt | chimeric, wt/-9/-7 | het, wt/-5 |
| 1 | 315 | IV | 84 | wt | chimeric, -85/-7/wt | het, wt/-5 |
| 1 | 316 | IV | 61 | wt | chimeric, wt/-3/-7 | chimeric, wt/-6/-5 |
| 1 | 317 | IV | 36 | wt | het, wt/-7 | wt |
| 1 | 318 | III | 26 | wt | chimeric, wt/-124/-7 | het, wt/-5 |
| 1 | 319 | I | 102 | wt | chimeric, wt/-10/-9 | wt |
| 1 | 320 | IV | 84 | wt | chimeric, wt/-13/-5 | chimeric, wt/-9/-5 |
| 1 | 321 | IV | 54 | wt | het, wt/-11 | wt |
| 1 | 322 | I | 103 | wt | chimeric, wt/-17/-6 | chimeric, wt/-3/-12 |
| 1 | 323 | IV | 55 | het, wt/-7 | chimeric, wt/-7/-11 | wt |
| 1 | 324 | III | 98 | wt | chimeric, -8/-7/wt | wt |
| 1 | 325 | IV | 84 | wt | het, wt/-67 | wt |
| 1 | 326 | III | 26 | wt | wt | wt |
| 1 | 327 | III | 98 | wt | chimeric, -191/wt/-6 | chimeric, wt/-191/s2,-103 |
| 1 | 328 | IV | 34 | wt | chimeric, -7/wt/-10 | het, wt/-5 |
| 1 | 329 | II | 43 | wt | chimeric, wt/-9/-7 | wt |
| 1 | 330 | III | 46 | chimeric, -4/-34/wt | chimeric, -15/-8/wt | chimeric, -5/-12/wt |
| 1 | 331 | IV | 55 | wt | chimeric, -8/wt/-9 | het, wt/-5 |
| 1 | 332 | II | 2 | wt | chimeric, wt/-7/-7 | wt |
| 1 | 333 | III | 24 | wt | chimeric, -9/-7/wt | het, wt/-6 |
| 1 | 334 | III | 28 | wt | chimeric, -11/-7/wt | het, wt/-5 |
| 1 | 335 | 0 | 70 | wt | het, wt/-11 | wt |
| 1 | 336 | IV | 58 | wt | wt | wt |
| 1 | 337 | IV | 59 | wt | het, -7/wt | wt |
| 1 | 338 | IV | 36 | wt | het, wt/-6 | het, wt/-5 |
| 1 | 339 | I | 9 | wt | chimeric, wt/-6/-14 | chimeric, wt/-3/-5 |
| 1 | 340 | 00 | 90 | wt | chimeric, wt/-16/-5 | wt |
| 1 | 341 | V | 67 | wt | chimeric, -10/wt/-7 | chimeric, wt/-4/-19 |
| 1 | 342 | III | 29 | wt | het, wt/-6 | wt |
| 1 | 343 | 0 | 72 | wt | chimeric, wt/-13/-44 | het, wt/-6 |
| 1 | 344 | 00 | 90 | wt | het, wt/-7 | het, wt/-5 |
| 1 | 345 | V | 91 | wt | het, -7/wt | chimeric, wt/-26/-15 |
| 1 | 346 | IV | 31 | wt | chimeric, -56/wt/-7 | het, wt/-5 |
| 1 | 347 | 0 | 72 | wt | het, -7/wt | chimeric, wt/-9/-94 |
| 1 | 348 | I | 9 | wt | chimeric, -30/s1,-10/wt | wt |
| 1 | 349 | IV | 58 | wt | het, -8/wt | chimeric, wt/-5/-7 |
| 1 | 350 | I | 32 | het, wt/-15 | biallelic, -282/-7 | biallelic, -282/-5 |
| 1 | 351 | IV | 57 | wt | chimeric, -219/wt/-11 | chimeric, wt/-219/-5 |
| 1 | 352 | III | 29 | wt | chimeric, wt/-8/-7/-7 | wt |
| 1 | 353 | III | 48 | wt | chimeric, -7/-19/-9 | chimeric, -84/-5/-8 |
| 1 | 354 | VII | 95 | het, wt/-12 | chimeric, wt/-7/-7 | chimeric, wt/-50/-40 |
| 1 | 355 | III | 27 | wt | chimeric, wt/s1,-15/-6 | wt |
| 1 | 356 | III | 20 | wt | het, s4,s1,s2,-45/wt | wt |
| 1 | 357 | III | 27 | wt | het, -7/wt | chimeric, wt/-10/-6 |
| 1 | 358 | IV | 57 | wt | chimeric, -10/-8/wt | wt |
| 1 | 359 | III | 25 | wt | het, -7/wt | chimeric, wt/-17/-5 |
| 1 | 360 | IV | 64 | het, wt/-3 | het, wt/s1,-5 | wt |
| 1 | 361 | II | 80 | wt | chimeric, wt/-8/-7 | wt |
| 1 | 362 | IV | 86 | wt | chimeric, -46/wt/-7 | wt |
| 1 | 363 | III | 48 | wt | het, wt/-7 | wt |
| 1 | 364 | IV | 61 | wt | wt | wt |
| 1 | 365 | III | 19 | wt | chimeric, wt/-7/-92 | wt |
| 1 | 366 | IV | 79 | wt | wt | wt |
| 1 | 367 | II | 56 | wt | homo, -7 | homo, -5 |
| 1 | 368 | 0 | 68 | wt | het, wt/-7 | het, wt/-5 |
| 1 | 369 | III | 48 | het, wt/-7 | het, -6/wt | het, wt/-6 |
| 1 | 370 | I | 100 | het, wt/s1,-163 | het, s1,-163/wt | het, wt/-5 |
| 1 | 371 | I | 100 | wt | chimeric, -16/-7/wt | het, wt/-58 |
| 1 | 372 | III | 51 | wt | chimeric, wt/s1,-11/-7 | chimeric, wt/-5/-5 |
| 1 | 373 | III | 46 | wt | het, -7/wt | chimeric, -227/wt/-16 |
| 1 | 374 | I | 9 | wt | chimeric, wt/-37/-31 | wt |
| 1 | 375 | II | 80 | wt | het, -16,s1/wt | wt |
| 1 | 376 | IV | 58 | wt | wt | wt |
| 1 | 377 | I | 11 | wt | chimeric, -40/-134/wt | het, wt/-5 |
| 1 | 378 | IV | 81 | wt | het, wt/-7 | wt |
| 1 | 379 | V | 67 | wt | het, wt/-7 | wt |
| 1 | 380 | II | 15 | wt | chimeric, wt/-23/-13 | chimeric, wt/-6/-5 |
| 1 | 381 | II | 43 | wt | chimeric, wt/-158/-7 | wt |
| 1 | 382 | III | 22 | wt | chimeric, wt/-7/-6 | wt |
| 1 | 383 | III | 17 | wt | het, -6/wt | chimeric, -6/wt/-27 |
| 1 | 384 | II | 80 | wt | chimeric, -44/wt/-7 | wt |
| 1 | 385 | IV | 34 | wt | chimeric, -25/-8/wt | het, wt/-5 |
| 1 | 386 | III | 25 | wt | chimeric, -196/-7/wt | chimeric, -196/wt/-5 |
| 1 | 387 | III | 50 | wt | wt | wt |
| 1 | 388 | III | 48 | wt | het, -7/wt | wt |
| 1 | 389 | V | 65 | wt | chimeric, wt/-11/-28 | het, wt/-5 |
| 1 | 390 | 0 | 41 | wt | het, -7/wt | wt |
| 1 | 391 | IV | 58 | wt | chimeric, -36/-13/wt | het, wt/-5 |
| 1 | 392 | 0 | 39 | wt | chimeric, -10/-7/wt | het, wt/-5 |
| 1 | 393 | IV | 55 | wt | chimeric, wt/-13/-22 | het, wt/-5 |
| 1 | 394 | IV | 34 | wt | biallelic, -11/-6 | chimeric, -21/wt/-3 |
| 1 | 395 | III | 97 | wt | wt | wt |
| 1 | 396 | IV | 55 | wt | het, -15/wt | het, wt/-5 |
| 1 | 397 | V | 66 | wt | chimeric, -8/-5/wt | wt |
| 1 | 398 | V | 67 | wt | chimeric, wt/-8/-10 | chimeric, wt/-9/-5 |
| 1 | 399 | I | 73 | wt | chimeric, s2,s1,-4/-39/wt | het, wt/-5 |
| 1 | 400 | III | 20 | wt | homo, -7 | het, -5/wt |
| 1 | 401 | I | 89 | wt | wt | wt |
| 1 | 402 | 00 | 90 | wt | het, wt/-7 | het, wt/-6 |
| 1 | 403 | III | 27 | wt | het, wt/-7 | het, wt/-5 |
| 1 | 404 | IV | 58 | wt | het, wt/-7 | wt |
| 1 | 405 | I | 101 | het, -9/wt | het, -10/wt | het, -5/wt |
| 1 | 406 | 00 | 90 | wt | chimeric, wt/-7/-10 | het, wt/-8 |
| 1 | 407 | III | 22 | wt | chimeric, wt/-6/-5 | wt |
| 1 | 408 | IV | 64 | wt | het, wt/-7 | wt |
| 1 | 409 | IV | 84 | wt | wt | het, -26/wt |
| 1 | 410 | III | 22 | wt | chimeric, -143/wt/-6 | het, wt/-5 |
| 1 | 411 | II | 80 | wt | chimeric, -15/wt/-7 | het, s1,-668/wt |
| 1 | 412 | III | 19 | wt | chimeric, -9/-222/wt | het, wt/-222 |
| 1 | 413 | II | 56 | wt | chimeric, -7/wt/-6 | wt |
| 1 | 414 | III | 28 | wt | het, wt/-154 | chimeric, wt/-5/-5 |
| 1 | 415 | III | 22 | wt | chimeric, wt/-41/-25 | wt |
| 1 | 416 | 00 | 76 | wt | chimeric, -6/-9/wt | wt |
| 1 | 417 | II | 15 | wt | chimeric, -8/-7/wt | chimeric, -8/-26/wt |
| 1 | 418 | IV | 36 | wt | het, wt/-6 | wt |
| 1 | 419 | V | 92 | wt | chimeric, -7/-187/-8 | chimeric, wt/-12/-187 |
| 1 | 420 | IV | 55 | wt | wt | wt |
| 1 | 421 | I | 32 | wt | het, -7/wt | het, -46/wt |
| 1 | 422 | III | 28 | wt | chimeric, -17/wt/-7 | het, wt/-9 |
| 1 | 423 | I | 101 | wt | chimeric, wt/-11/-31 | het, -5/wt |
| 1 | 424 | IV | 49 | het, wt/-21 | chimeric, -31/wt/-6 | het, -5/wt |
| 1 | 425 | III | 28 | wt | het, -7/wt | wt |
| 1 | 426 | IV | 58 | wt | chimeric, wt/-13/-181 | chimeric, wt/-5/-181 |
| 1 | 427 | II | 7 | wt | chimeric, -46/wt/-7 | wt |
| 1 | 428 | I | 101 | wt | chimeric, -11/-21/wt | chimeric, -6/-55/wt |
| 1 | 429 | 0 | 37 | wt | chimeric, -10/wt/-11 | het, wt/-5 |
| 1 | 430 | II | 2 | wt | het, wt/-7 | wt |
| 1 | 431 | III | 87 | het, wt/-700 | chimeric, -700/wt/-7 | chimeric, wt/-700/-4 |
| 1 | 432 | V | 67 | wt | het, wt/-7 | wt |
| 1 | 433 | III | 50 | wt | chimeric, -48/-7/wt | wt |
| 1 | 434 | III | 87 | wt | biallelic, -7/-12 | biallelic, -3/-5 |
| 1 | 435 | VI | 93 | wt | chimeric, -10/wt/-45,s1 | wt |
| 1 | 436 | IV | 58 | wt | chimeric, -13/-101/wt | chimeric, -13/wt/-2 |
| 1 | 437 | III | 97 | chimeric, wt/-547/-8 | chimeric, s2,-7/-9/-547/-7 | chimeric, wt/-547/-104 |
| 1 | 438 | III | 20 | wt | het, -7/wt | chimeric, -135/wt/s1,-1/s1 |
| 1 | 439 | I | 74 | wt | chimeric, -11/-7/wt | wt |
| 1 | 440 | I | 100 | wt | het, -175/wt | wt |
| 1 | 441 | III | 24 | wt | chimeric, wt/-11/-9 | het, wt/-5 |
| 1 | 442 | II | 56 | wt | chimeric, -13/-7/wt | het, -3/wt |
| 1 | 443 | II | 38 | wt | chimeric, wt/-7/-8 | het, wt/-5 |
| 1 | 444 | IV | 52 | wt | chimeric, -183/-14/wt | chimeric, -183/-6/wt |
| 1 | 445 | II | 7 | het, wt/-7 | chimeric, -14/-7/-4/-8 | chimeric, -12/-14/wt/-5 |
| 1 | 446 | III | 48 | wt | het, wt/-4 | wt |
| 1 | 447 | III | 45 | wt | het, -8/wt | het, wt/-5 |
| 1 | 448 | III | 26 | wt | chimeric, -8/wt/-132 | wt |
| 1 | 449 | IV | 54 | wt | het, -20/wt | wt |
| 1 | 450 | III | 50 | wt | chimeric, -11/-7/wt | het, wt/-15 |
| 1 | 451 | III | 24 | wt | het, wt/-11 | het, wt/-5 |
| 1 | 452 | 0 | 40 | wt | het, -10/wt | chimeric, -5/-9/wt |
| 1 | 453 | III | 47 | wt | chimeric, wt/-6,s1,s1,-2,s1/s1,-10 | chimeric, wt/-6/-108 |
| 1 | 454 | I | 9 | wt | chimeric, -9/-7/wt | het, wt/-9 |
| 1 | 455 | 0 | 39 | wt | chimeric, wt/-8/-12 | het, wt/-10 |
| 1 | 456 | 0 | 41 | wt | chimeric, wt/s3,+17,s2/-800 | het, wt/-800 |
| 1 | 457 | IV | 52 | wt | chimeric, -13/wt/-13/-7 | wt |
| 1 | 458 | I | 42 | wt | chimeric, -7/-35/-11 | het, wt/-1 |
| 1 | 459 | V | 67 | het, wt/-6 | chimeric, wt/-7/-6 | wt |
| 1 | 460 | IV | 57 | wt | chimeric, wt/-25/-1 | chimeric, wt/-21/-5 |
| 1 | 461 | I | 32 | wt | het, wt/-8 | wt |
| 1 | 462 | I | 89 | wt | het, -7/wt | chimeric, wt/-9/-5 |
| 1 | 463 | II | 80 | wt | chimeric, -13/-7/wt | wt |
| 1 | 464 | II | 80 | wt | wt | het, wt/-5 |
| 1 | 465 | I | 103 | wt | wt | wt |
| 1 | 466 | VII | 96 | wt | het, wt/-7 | wt |
| 1 | 467 | IV | 58 | wt | chimeric, -10/-6/wt | het, wt/-5 |
| 1 | 468 | IV | 55 | wt | het, -7/wt | het, wt/-5 |
| 1 | 469 | IV | 59 | wt | chimeric, -13/-7/wt | chimeric, wt/-5/-5 |
| 1 | 470 | I | 102 | wt | het, wt/-7 | wt |
| 1 | 471 | III | 28 | wt | chimeric, -13/-10/wt | chimeric, wt/-18/s1,-14 |
| 1 | 472 | IV | 36 | wt | het, wt/-7 | wt |
| 1 | 473 | IV | 34 | wt | chimeric, -109/-7/wt | het, wt/-5 |
| 1 | 474 | III | 48 | wt | chimeric, -8/-9/wt | het, wt/-5 |
| 1 | 475 | III | 19 | het, wt/-6 | chimeric, -17,s1/-11/wt | chimeric, -7/wt/-5 |
| 1 | 476 | IV | 79 | wt | het, -7/wt | chimeric, wt/-5/-5 |
| 1 | 477 | 00 | 77 | wt | chimeric, wt/-7/s1,-8 | wt |
| 1 | 478 | IV | 36 | wt | chimeric, -29/-11/wt | het, wt/-23 |
| 1 | 479 | II | 3 | wt | het, -27/wt | het, wt/-5 |
| 1 | 480 | 0 | 40 | wt | het, wt/-7 | wt |
| 1 | 481 | I | 100 | wt | het, wt/-7 | het, wt/-5 |
| 1 | 482 | IV | 64 | chimeric, -9/-3/wt | chimeric, -4/-7/wt | chimeric, -5/-6/wt |
| 1 | 483 | II | 38 | wt | het, wt/-9 | wt |
| 1 | 484 | III | 19 | wt | chimeric, -7/s1,-9,s1,-4/wt | het, wt/-5 |
| 1 | 485 | IV | 61 | het, wt/-2 | chimeric, -7/-8/wt | wt |
| 1 | 486 | 0 | 40 | wt | chimeric, -8/-72/wt | het, -8/wt |
| 1 | 487 | IV | 81 | wt | het, -7/wt | wt |
| 1 | 488 | IV | 64 | wt | chimeric, -6/-29/wt | het, wt/-27 |
| 1 | 489 | IV | 52 | chimeric, -208/-4/wt | chimeric, -208/-20,-180/wt | chimeric, -54/-20,-180/wt |
| 1 | 490 | 0 | 70 | wt | het, -7/wt | het, wt/-5 |
| 1 | 491 | IV | 52 | wt | het, -9/wt | wt |
| 1 | 492 | III | 24 | wt | het, -7/wt | chimeric, -5/-10/wt |
| 1 | 493 | IV | 81 | wt | het, wt/-7 | het, wt/-3 |
| 1 | 494 | V | 91 | wt | het, wt/-7 | wt |
| 1 | 495 | IV | 52 | wt | chimeric, wt/-7/-6 | wt |
| 1 | 496 | V | 65 | wt | het, -7/wt | het, wt/-5 |
| 1 | 497 | I | 104 | wt | chimeric, wt/-6/-7 | het, wt/-5 |
| 1 | 498 | 0 | 37 | wt | het, -7/wt | het, wt/-5 |
| 1 | 499 | III | 25 | wt | chimeric, -200/s2,-10/wt | het, wt/-200 |
| 1 | 500 | IV | 31 | wt | chimeric, wt/-13/-41 | het, wt/-5 |
| 1 | 501 | IV | 52 | wt | het, wt/-7 | wt |
| 1 | 502 | 0 | 70 | het, wt/-32 | chimeric, wt/-7/-6 | chimeric, wt/-9/-16 |
| 1 | 503 | III | 99 | wt | chimeric, wt/-59/-10/-21 | het, wt/-3 |
| 1 | 504 | V | 66 | het, wt/-975 | chimeric, -975/wt/-7 | chimeric, wt/-975/-5 |
| 1 | 505 | III | 25 | wt | het, -7/wt | het, wt/-5 |
| 1 | 506 | IV | 52 | wt | chimeric, wt/-15/-7 | chimeric, wt/-5/-5 |
| 1 | 507 | 0 | 70 | wt | het, wt/-7 | het, wt/-5 |
| 1 | 508 | V | 91 | wt | het, wt/-7 | wt |
| 1 | 509 | II | 3 | wt | wt | wt |
| 1 | 510 | V | 67 | wt | het, wt/-7 | wt |
| 1 | 511 | IV | 86 | wt | chimeric, wt/-7/-9 | het, wt/-5 |
| 1 | 512 | V | 66 | wt | chimeric, wt/-40/-7 | wt |
| 1 | 513 | IV | 34 | wt | chimeric, -9/-7/wt | chimeric, -10/wt/-27/-5 |
| 1 | 514 | I | 100 | wt | chimeric, wt/-15/-7 | wt |
| 1 | 515 | IV | 31 | wt | chimeric, -8/-9/wt | het, wt/-5 |
| 1 | 516 | IV | 36 | wt | het, -8/wt | het, -51/wt |
| 1 | 517 | IV | 79 | wt | homo, -7 | not determined |
| 1 | 518 | III | 22 | wt | not determined | wt |
| 1 | 519 | III | 28 | wt | wt | wt |
| 1 | 520 | II | 6 | wt | chimeric, -8/-7/wt | wt |
| 1 | 521 | III | 28 | wt | chimeric, wt/-10/-6 | wt |
| 1 | 522 | II | 3 | wt | het, -7/wt | chimeric, -43/-5/wt |
| 1 | 523 | V | 66 | wt | chimeric, wt/-39/-7/-13 | het, wt/-5 |
| 1 | 524 | IV | 59 | het, wt/-3 | chimeric, -7/wt/s1,s1,+3 | chimeric, -8/-22/wt |
| 1 | 525 | V | 67 | wt | chimeric, -16/wt/-196 | het, wt/-196 |
| 1 | 526 | IV | 58 | wt | chimeric, wt/-22/-10 | wt |
| 1 | 527 | III | 21 | wt | chimeric, -27/wt/-9 | wt |
| 1 | 528 | III | 17 | wt | chimeric, -7/wt/-5 | het, wt/-49 |
| 1 | 529 | III | 18 | wt | wt | wt |
| 1 | 530 | I | 75 | wt | chimeric, -16/wt/-10 | chimeric, wt/-9/-5 |
| 1 | 531 | IV | 34 | wt | het, wt/-6 | het, wt/-5 |
| 1 | 532 | IV | 34 | wt | chimeric, -11/-9/wt | het, wt/-22 |
| 1 | 533 | IV | 31 | wt | chimeric, wt/-6/-185 | wt |
| 1 | 534 | VII | 95 | wt | biallelic, -11/-9 | het, wt/-5 |
| 1 | 535 | IV | 81 | wt | het, wt/-7 | het, wt/-9 |
| 1 | 536 | IV | 82 | wt | chimeric, wt/-7/-6 | het, wt/-7 |
| 1 | 537 | III | 26 | wt | het, -40/wt | het, wt/-6 |
| 1 | 538 | III | 47 | wt | chimeric, -227/-12/wt | chimeric, wt/-227/-35 |
| 1 | 539 | II | 44 | wt | chimeric, -188/wt/-18 | het, wt/-188 |
| 1 | 540 | IV | 61 | wt | het, -7/wt | wt |
| 1 | 541 | III | 28 | het, wt/-6 | chimeric, -18/-7/wt | chimeric, s1,-5/-7/wt |
| 1 | 542 | IV | 79 | wt | chimeric, s2,-24/-11/wt | het, wt/-5 |
| 1 | 543 | 00 | 90 | wt | het, wt/-7 | wt |
| 1 | 544 | I | 100 | wt | chimeric, -20/-31/-8/-13 | biallelic, -5/-5 |
| 1 | 545 | I | 32 | wt | het, -7/wt | chimeric, -30/-38/wt |
| 1 | 546 | III | 29 | wt | biallelic, -7/-53 | chimeric, -5/-3/-5 |
| 1 | 547 | IV | 36 | wt | chimeric, wt/-5/-7 | chimeric, wt/-60/-10 |
| 1 | 548 | IV | 63 | wt | chimeric, wt/-29/-7 | wt |
| 1 | 549 | IV | 55 | wt | chimeric, -3/-14,s1,s1/wt | het, wt/-5 |
| 1 | 550 | IV | 79 | wt | het, -7/wt | het, wt/-5 |
| 1 | 551 | III | 17 | wt | chimeric, -11/-39,s1/wt | chimeric, wt/-5/-9 |
| 1 | 552 | II | 14 | wt | het, wt/-7 | wt |
| 1 | 553 | 00 | 77 | wt | chimeric, -18/s1,-10/wt | chimeric, -5/-5/wt |
| 1 | 554 | III | 50 | wt | chimeric, -11/wt/-6 | chimeric, wt/-6/-5 |
| 1 | 555 | 00 | 77 | wt | chimeric, wt/-31/-6 | het, wt/-36 |
| 1 | 556 | III | 17 | wt | chimeric, -11/-7/wt | chimeric, wt/-13/-83 |
| 1 | 557 | IV | 34 | wt | chimeric, -10/wt/-7 | chimeric, -17/wt/-7 |
| 1 | 558 | II | 14 | wt | chimeric, -37/-6/-8 | chimeric, -5/wt/-5/-6 |
| 1 | 559 | II | 6 | wt | chimeric, wt/-13,s1,s2/-6 | wt |
| 1 | 560 | III | 28 | wt | chimeric, -16,s2/wt/s2,+2,s1,s1,+16,s1 | chimeric, -5/wt/s2,+18,s2,s2,s2 |
| 1 | 561 | III | 98 | het, wt/-12 | chimeric, -8/-10/wt | het, wt/-5 |
| 1 | 562 | IV | 86 | wt | chimeric, wt/s1,-11/-9 | wt |
| 1 | 563 | III | 29 | het, wt/-4 | biallelic, -7/-13 | chimeric, wt/-11/-10/-4 |
| 1 | 564 | II | 15 | wt | chimeric, wt/-10/-7 | wt |
| 1 | 565 | I | 32 | wt | chimeric, -7/-6/wt | wt |
| 1 | 566 | III | 28 | wt | chimeric, -11/-7/wt | wt |
| 1 | 567 | IV | 86 | wt | het, wt/-7 | wt |
| 1 | 568 | III | 98 | het, wt/-343 | het, -343/wt | het, wt/-343 |
| 1 | 569 | III | 47 | wt | chimeric, -8/-7/wt | wt |
| 1 | 570 | IV | 36 | wt | het, -26/wt | het, wt/-3 |
| 1 | 571 | III | 28 | wt | wt | wt |
| 1 | 572 | IV | 31 | wt | biallelic, -7/-10 | homo, -5 |
| 1 | 573 | I | 75 | wt | chimeric, -272/-8/-12/-8 | chimeric, -272/-5/-8 |
| 1 | 574 | III | 22 | wt | het, wt/-10 | het, wt/-36 |
| 1 | 575 | IV | 81 | wt | chimeric, -7/-55/wt | wt |
| 1 | 576 | III | 28 | wt | chimeric, -4/wt/-7 | wt |
| 1 | 577 | IV | 54 | wt | wt | wt |
| 1 | 578 | III | 48 | wt | het, wt/-7 | het, wt/-5 |
| 1 | 579 | I | 100 | wt | het, wt/-6 | chimeric, wt/-5/-30 |
| 1 | 580 | III | 53 | wt | chimeric, wt/-54/-7 | wt |
| 1 | 581 | III | 50 | wt | chimeric, wt/-49/-16 | het, wt/-21 |
| 1 | 582 | IV | 31 | wt | chimeric, -7/-12/-16 | chimeric, -3/-32/-5 |
| 1 | 583 | V | 66 | wt | chimeric, -8/-12/wt | chimeric, -6/wt/-9 |
| 1 | 584 | IV | 34 | wt | het, wt/-7 | het, wt/-5 |
| 1 | 585 | II | 6 | wt | chimeric, wt/-11/-8 | chimeric, wt/-25/-16 |
| 1 | 586 | II | 12 | wt | wt | wt |
| 1 | 587 | I | 102 | wt | het, wt/-5 | wt |
| 1 | 588 | II | 80 | wt | homo, -7 | het, wt/-5 |
| 1 | 589 | 00 | 90 | wt | chimeric, -7/-18/wt | chimeric, -3/-19/wt |
| 1 | 590 | IV | 85 | wt | chimeric, -71/wt/-9/-64 | wt |
| 1 | 591 | I | 42 | wt | homo, -7 | het, -9/wt |
| 1 | 592 | I | 89 | wt | wt | wt |
| 1 | 593 | I | 10 | wt | het, wt/-7 | wt |
| 1 | 594 | I | 75 | het, -9/wt | biallelic, -189/-11 | homo, -189 |
| 1 | 595 | III | 18 | wt | het, wt/-7 | het, wt/-5 |
| 1 | 596 | IV | 59 | wt | chimeric, -10/s1,-12/-18/-17 | wt |
| 1 | 597 | III | 30 | wt | chimeric, -7/wt/-10/-122 | wt |
| 1 | 598 | III | 18 | wt | het, -6/wt | wt |
| 1 | 599 | 00 | 78 | wt | chimeric, -185/-6/-5 | chimeric, -9/-185/wt |
| 1 | 600 | II | 80 | wt | chimeric, wt/-8/-6 | wt |
| 1 | 601 | 0 | 88 | het, wt/-329 | chimeric, -329/-11/wt | chimeric, -3/wt/-26 |
| 1 | 602 | IV | 81 | wt | chimeric, -64/-12/wt | wt |
| 1 | 603 | II | 33 | wt | het, -7/wt | het, -5/wt |
| 1 | 604 | I | 100 | wt | chimeric, wt/-11/-67/-13 | het, wt/-5 |
| 1 | 605 | III | 45 | wt | chimeric, wt/-14/-5 | het, wt/-5 |
| 1 | 606 | III | 50 | wt | het, wt/-7 | wt |
| 1 | 607 | IV | 58 | chimeric, -3/wt/-784 | chimeric, -189/wt/-784 | chimeric, -5/-189/wt |
| 1 | 608 | III | 17 | wt | chimeric, -114/-31/wt | chimeric, wt/-27/-6/-99 |
| 1 | 609 | III | 87 | wt | het, wt/-7 | wt |
| 1 | 610 | IV | 58 | wt | het, -13/wt | het, -5/wt |
| 1 | 611 | I | 89 | wt | chimeric, -74/s1,-10/wt | wt |
| 1 | 612 | 0 | 37 | wt | chimeric, -8/-10/wt | wt |
| 1 | 613 | III | 26 | wt | chimeric, -18/wt/-7 | het, wt/-5 |
| 1 | 614 | II | 80 | wt | chimeric, -15,s1/-18/wt | wt |
| 1 | 615 | IV | 81 | wt | chimeric, wt/-7/-256 | het, wt/-256 |
| 1 | 616 | 00 | 77 | wt | het, wt/-7 | wt |
| 1 | 617 | IV | 54 | wt | het, wt/-7 | wt |
| 1 | 618 | III | 24 | wt | het, -9/wt | het, wt/-5 |
| 1 | 619 | I | 103 | wt | het, wt/-7 | wt |
| 1 | 620 | I | 89 | wt | het, wt/-7 | wt |
| 1 | 621 | III | 99 | chimeric, wt/-3/-7 | chimeric, wt/-8/-27 | chimeric, wt/-80/-6 |
| 1 | 622 | 0 | 88 | wt | chimeric, -8/-7/wt | wt |
| 1 | 623 | V | 66 | wt | chimeric, -17/wt/-7 | het, wt/-5 |
| 1 | 624 | II | 56 | wt | wt | wt |
| 1 | 625 | IV | 84 | wt | het, -6/wt | het, wt/-5 |
| 1 | 626 | II | 80 | wt | het, -299/wt | het, -299/wt |
| 1 | 627 | II | 38 | wt | wt | het, wt/-5 |
| 1 | 628 | V | 65 | wt | het, wt/-7 | wt |
| 1 | 629 | II | 3 | wt | chimeric, -10/-7/wt | chimeric, -5/-19/wt |
| 1 | 630 | I | 73 | wt | chimeric, wt/-13/-7 | wt |
| 1 | 631 | IV | 85 | wt | chimeric, -66/-10/wt | chimeric, -60/wt/-5 |
| 1 | 632 | 0 | 70 | wt | chimeric, wt/-8/-7 | het, wt/-5 |
| 1 | 633 | V | 65 | wt | chimeric, -20/-8/wt | chimeric, -5/-3/wt |
| 1 | 634 | III | 21 | wt | chimeric, -25/wt/-6 | het, wt/-5 |
| 1 | 635 | II | 14 | wt | biallelic, s2,-27/-11 | het, wt/-5 |
| 1 | 636 | III | 28 | wt | chimeric, wt/-6/-216 | het, wt/-216 |
| 1 | 637 | IV | 58 | wt | het, -7/wt | wt |
| 1 | 638 | III | 25 | wt | chimeric, s1,-9/-10/wt | het, wt/-9 |
| 1 | 639 | IV | 35 | wt | biallelic, -12/-8 | biallelic, -17/-30 |
| 1 | 640 | IV | 79 | het, wt/-628 | het, -7/wt | wt |
| 1 | 641 | II | 6 | wt | chimeric, -8/-7/wt | wt |
| 1 | 642 | VII | 95 | wt | het, wt/-7 | wt |
| 1 | 643 | 0 | 72 | wt | chimeric, -9/-7/wt | het, wt/-7 |
| 1 | 644 | IV | 79 | wt | chimeric, -9/s1,-13/wt | het, wt/-5 |
| 1 | 645 | IV | 61 | wt | chimeric, wt/-9/-9 | het, wt/-26 |
| 1 | 646 | III | 48 | wt | chimeric, -9/wt/-14/-163 | het, wt/-47 |
| 1 | 647 | IV | 34 | wt | het, -6/wt | wt |
| 1 | 648 | IV | 52 | wt | het, wt/-7 | het, wt/-15 |
| 1 | 649 | 00 | 77 | wt | chimeric, -13/-18/wt | het, wt/-14 |
| 1 | 650 | III | 29 | wt | het, wt/-7 | wt |
| 1 | 651 | IV | 79 | wt | chimeric, wt/-3/-13/s1,-10 | chimeric, wt/-3/-9 |
| 1 | 652 | II | 3 | wt | chimeric, -11/wt/-17 | chimeric, wt/-5/-5/-43 |
| 1 | 653 | III | 19 | het, wt/-5 | het, -109/wt | het, wt/-6 |
| 1 | 654 | IV | 54 | wt | biallelic, -9/-7 | chimeric, -6/-5/wt |
| 1 | 655 | II | 13 | wt | chimeric, -7/-16/-17/-25 | biallelic, -5/-6 |
| 1 | 656 | 0 | 40 | chimeric, -340/-6/wt | chimeric, -340/-7/wt | chimeric, -340/-22/wt |
| 1 | 657 | IV | 34 | wt | het, wt/-12 | het, wt/-55 |
| 1 | 658 | VI | 93 | wt | wt | wt |
| 1 | 659 | V | 67 | wt | het, wt/-7 | wt |
| 1 | 660 | IV | 52 | het, wt/-18 | homo, -7 | biallelic, -6/-5 |
| 1 | 661 | III | 47 | wt | het, wt/-7 | het, wt/-5 |
| 1 | 662 | IV | 61 | wt | chimeric, -7/wt/-30/-16 | chimeric, wt/-6/-5 |
| 1 | 663 | III | 27 | het, wt/-3 | het, -7/wt | chimeric, -5/wt/-32 |
| 1 | 664 | 0 | 37 | wt | het, wt/-7 | wt |
| 1 | 665 | II | 7 | wt | chimeric, -36/wt/-9 | het, wt/-5 |
| 1 | 666 | IV | 52 | wt | chimeric, wt/-7/-6 | chimeric, wt/-9/-27 |
| 1 | 667 | II | 12 | wt | chimeric, wt/-8/-19 | chimeric, wt/-12/-5 |
| 1 | 668 | III | 46 | wt | chimeric, wt/-11/-6 | chimeric, wt/-19/-27 |
| 1 | 669 | II | 7 | wt | het, -114/wt | het, wt/-6 |
| 1 | 670 | 0 | 41 | wt | het, -10/wt | het, wt/-5 |
| 1 | 671 | IV | 34 | wt | chimeric, -6/-7/wt | chimeric, -56/-5/wt |
| 1 | 672 | I | 89 | wt | het, wt/-7 | het, wt/-5 |
| 1 | 673 | 00 | 90 | wt | chimeric, -7/wt/-6 | het, wt/-5 |
| 1 | 674 | IV | 34 | wt | het, wt/-7 | chimeric, wt/-9/-34 |
| 1 | 675 | V | 67 | wt | het, wt/-7 | het, wt/-5 |
| 1 | 676 | IV | 81 | wt | chimeric, wt/-9/-6 | het, wt/-5 |
| 1 | 677 | II | 3 | wt | het, wt/-7 | wt |
| 1 | 678 | III | 53 | wt | chimeric, -29,s2/-7/wt | het, wt/-5 |
| 1 | 679 | III | 98 | wt | het, wt/-7 | wt |
| 1 | 680 | V | 67 | wt | het, wt/-7 | wt |
| 1 | 681 | I | 74 | wt | chimeric, wt/-15/-5 | wt |
| 1 | 682 | III | 28 | wt | chimeric, -7/wt/-7 | het, wt/-10 |
| 1 | 683 | III | 98 | wt | chimeric, -10/-7/wt | chimeric, -350/wt/-5 |
| 1 | 684 | IV | 36 | wt | het, wt/-7 | wt |
| 1 | 685 | III | 24 | wt | het, wt/-7 | wt |
| 1 | 686 | IV | 61 | wt | chimeric, -334/-12/wt | chimeric, -334/wt/-5 |
| 1 | 687 | II | 15 | wt | het, wt/-1 | wt |
| 1 | 688 | V | 92 | wt | chimeric, wt/-36/-7 | wt |
| 1 | 689 | I | 89 | wt | chimeric, wt/-8/-7 | chimeric, wt/-6/-12 |
| 1 | 690 | III | 28 | wt | chimeric, wt/-7/-7 | wt |
| 1 | 691 | V | 65 | wt | het, wt/-7 | wt |
| 1 | 692 | II | 38 | wt | chimeric, wt/-9/-6 | het, wt/-5 |
| 1 | 693 | IV | 36 | wt | chimeric, -27/wt/-7 | wt |
| 1 | 694 | II | 3 | wt | chimeric, wt/-10/-7 | chimeric, wt/-5/-26 |
| 1 | 695 | III | 99 | wt | het, wt/-7 | wt |
| 1 | 696 | II | 3 | wt | chimeric, -18,s1,-14/-7/-6 | wt |
| 1 | 697 | III | 48 | wt | het, wt/-5 | wt |
| 1 | 698 | 00 | 90 | wt | het, wt/-7 | wt |
| 1 | 699 | III | 25 | wt | chimeric, -10,s1,s2,-13/-10/wt | het, wt/-467 |
| 1 | 700 | V | 92 | wt | chimeric, -16/wt/-7 | chimeric, wt/-16/-14 |
| 1 | 701 | III | 26 | wt | het, wt/-15 | wt |
| 1 | 702 | I | 103 | wt | het, wt/-111 | het, wt/-13 |
| 1 | 703 | I | 89 | wt | chimeric, -27/wt/-10 | het, wt/-19 |
| 1 | 704 | III | 29 | wt | chimeric, -191/-185/wt | chimeric, -191/wt/-185 |
| 1 | 705 | I | 102 | wt | chimeric, wt/-7/-6 | het, -5/wt |
| 1 | 706 | I | 103 | wt | chimeric, -121/-7/wt | chimeric, -16/wt/-25,s2,s2 |
| 1 | 707 | 0 | 68 | wt | chimeric, -26/wt/-3 | wt |
| 1 | 708 | II | 15 | wt | chimeric, wt/-4/-22,s1,s1 | wt |
| 1 | 709 | II | 15 | wt | chimeric, wt/-9/-6 | het, wt/-9 |
| 1 | 710 | 0 | 72 | wt | het, wt/-5 | het, wt/-5 |
| 1 | 711 | III | 28 | wt | het, wt/-7 | wt |
| 1 | 712 | 00 | 77 | wt | chimeric, -186/-7/wt | het, -186/wt |
| 1 | 713 | III | 25 | wt | chimeric, wt/-13/-6 | wt |
| 1 | 714 | III | 99 | wt | chimeric, s1,-660/-21/wt | het, s1,-660/wt |
| 1 | 715 | II | 6 | wt | chimeric, -8/-7/wt | wt |
| 1 | 716 | 0 | 70 | wt | het, wt/-7 | chimeric, wt/-96/-5 |
| 1 | 717 | III | 20 | wt | chimeric, wt/-58/-6 | wt |
| 1 | 718 | IV | 57 | wt | chimeric, wt/-6/-12 | het, -5/wt |
| 1 | 719 | III | 17 | wt | chimeric, -7/-8/wt | wt |
| 1 | 720 | III | 30 | wt | chimeric, -7/wt/-6 | chimeric, -5/wt/-9 |
| 1 | 721 | IV | 79 | wt | chimeric, wt/-13/-8 | chimeric, wt/-25/-29 |
| 1 | 722 | IV | 34 | wt | chimeric, -190/-12/wt | het, wt/-190 |
| 1 | 723 | IV | 79 | wt | het, -7/wt | het, wt/-5 |
| 1 | 724 | V | 91 | wt | het, wt/s3,s1,+4,s1,s2,s2,s1 | het, wt/-5 |
| 1 | 725 | IV | 36 | wt | chimeric, -46/wt/-7 | het, wt/-3 |
| 1 | 726 | IV | 36 | wt | chimeric, -43/wt/-45 | wt |
| 1 | 727 | I | 42 | het, wt/-235 | chimeric, wt/-235/-13 | wt |
| 1 | 728 | I | 9 | wt | chimeric, -10/-16/-9 | wt |
| 1 | 729 | I | 103 | wt | het, wt/-7 | het, wt/-5 |
| 1 | 730 | II | 3 | wt | wt | wt |
| 1 | 731 | V | 67 | het, wt/-6 | chimeric, -12/-11/wt | chimeric, -10/wt/-5 |
| 1 | 732 | IV | 34 | wt | het, wt/-7 | wt |
| 1 | 733 | II | 3 | wt | chimeric, -7/-25/wt | het, -5/wt |
| 1 | 734 | V | 67 | wt | het, wt/-7 | wt |
| 1 | 735 | IV | 61 | wt | chimeric, -8/-7/wt | chimeric, -8/wt/-5 |
| 1 | 736 | I | 103 | wt | het, wt/-7 | wt |
| 1 | 737 | III | 26 | wt | het, wt/-7 | wt |
| 1 | 738 | III | 25 | wt | chimeric, -7/-18/wt | het, wt/-5 |
| 1 | 739 | 0 | 71 | wt | wt | wt |
| 1 | 740 | II | 3 | wt | chimeric, s1,-271/wt/-25 | het, wt/s1,-271 |
| 1 | 741 | II | 6 | wt | chimeric, -14/wt/-7 | het, wt/-5 |
| 1 | 742 | I | 9 | wt | het, -10/wt | wt |
| 1 | 743 | IV | 86 | wt | het, -7/wt | wt |
| 1 | 744 | I | 32 | wt | chimeric, -7/wt/-6 | chimeric, -27/wt/-5 |
| 1 | 745 | I | 89 | wt | chimeric, wt/-7/-13 | het, wt/-53 |
| 1 | 746 | V | 60 | wt | chimeric, wt/-7/-30 | chimeric, wt/-10/-19 |
| 1 | 747 | IV | 57 | wt | het, wt/-7 | wt |
| 1 | 748 | IV | 64 | wt | het, wt/-7 | wt |
| 1 | 749 | III | 30 | wt | chimeric, -6/wt/-7 | het, wt/-6 |
| 1 | 750 | V | 66 | wt | chimeric, -12/-6/wt | het, wt/-143 |
| 1 | 751 | III | 98 | wt | chimeric, -92/-9/wt | het, wt/-26 |
| 1 | 752 | IV | 58 | wt | chimeric, -25/-20/wt | wt |
| 1 | 753 | III | 48 | wt | chimeric, -10/wt/-19/-8 | chimeric, wt/-5/-5 |
| 1 | 754 | II | 56 | wt | chimeric, wt/-7/-5 | wt |
| 1 | 755 | II | 6 | wt | chimeric, wt/-13/-6 | het, wt/-5 |
| 1 | 756 | III | 21 | wt | chimeric, wt/-8/-16 | het, wt/-5 |
| 1 | 757 | II | 33 | wt | chimeric, -7/wt/-59 | het, wt/-5 |
| 1 | 758 | III | 20 | wt | chimeric, -18/-13/wt | het, wt/s1,-12 |
| 1 | 759 | III | 25 | wt | chimeric, -22/-42/wt | het, -5/wt |
| 1 | 760 | V | 67 | wt | het, wt/-7 | wt |
| 1 | 761 | II | 38 | wt | chimeric, wt/-7/-7/-10 | wt |
| 1 | 762 | II | 43 | wt | chimeric, -119/wt/-6 | het, -5/wt |
| 1 | 763 | IV | 54 | wt | chimeric, wt/-25/-10 | chimeric, wt/-5/-8 |
| 1 | 764 | 00 | 77 | wt | chimeric, -11/-7/wt | chimeric, -17/wt/-5 |
| 1 | 765 | III | 50 | wt | het, wt/-7 | het, wt/-5 |
| 1 | 766 | IV | 54 | wt | chimeric, wt/-7/-16 | wt |
| 1 | 767 | III | 46 | wt | het, -39/wt | het, wt/-26 |
| 1 | 768 | III | 87 | wt | chimeric, wt/-9/-6 | chimeric, wt/-3/-5 |
| 1 | 769 | II | 2 | wt | chimeric, -11/wt/-6 | het, wt/-6 |
| 1 | 770 | IV | 34 | wt | het, wt/-6 | wt |
| 1 | 771 | II | 7 | wt | het, wt/-7 | wt |
| 1 | 772 | II | 6 | wt | chimeric, -16/-6/wt | wt |
| 1 | 773 | V | 92 | wt | het, -7/wt | het, wt/-5 |
| 1 | 774 | I | 10 | wt | het, wt/-7 | wt |
| 1 | 775 | II | 15 | wt | wt | wt |
| 1 | 776 | III | 26 | wt | chimeric, -752/-11/wt | chimeric, -752/-5/wt |
| 1 | 777 | IV | 84 | wt | chimeric, wt/-12/-6 | chimeric, wt/-6/-26 |
| 1 | 778 | III | 22 | wt | het, -38/wt | het, wt/-5 |
| 1 | 779 | V | 65 | wt | het, -7,s3,s1/wt | het, -5/wt |
| 1 | 780 | III | 17 | wt | het, -11/wt | wt |
| 1 | 781 | II | 38 | wt | chimeric, -7/-8/wt | chimeric, wt/-5/-55 |
| 1 | 782 | I | 9 | wt | chimeric, wt/-7/-6 | wt |
| 1 | 783 | IV | 34 | wt | chimeric, wt/s1,-8/-9 | wt |
| 1 | 784 | III | 29 | het, wt/-6 | chimeric, -7/wt/-7 | chimeric, wt/-9/-4 |
| 1 | 785 | III | 98 | wt | chimeric, wt/-7/-10 | het, wt/-5 |
| 1 | 786 | IV | 61 | wt | het, -126/wt | chimeric, wt/-5/-5 |
| 1 | 787 | 00 | 77 | wt | het, -8/wt | het, wt/s1,-8 |
| 1 | 788 | I | 89 | wt | het, -7/wt | wt |
| 1 | 789 | III | 28 | wt | chimeric, -11/wt/-11 | wt |
| 1 | 790 | IV | 35 | wt | chimeric, wt/-13/-8 | chimeric, wt/-11/-26 |
| 1 | 791 | IV | 84 | wt | chimeric, -12/-6/wt | chimeric, -7/-5/wt |
| 1 | 792 | V | 60 | wt | chimeric, wt/-11/-11 | wt |
| 1 | 793 | II | 80 | wt | chimeric, wt/-16/-10 | wt |
| 1 | 794 | I | 89 | wt | chimeric, -18/-7/-203/-22 | chimeric, -5/wt/-5/-203 |
| 1 | 795 | I | 102 | wt | het, -10/wt | wt |
| 1 | 796 | II | 44 | wt | chimeric, wt/-9/-7 | wt |
| 1 | 797 | IV | 55 | wt | chimeric, -189/-17/wt | chimeric, -189/-3/wt |
| 1 | 798 | III | 28 | wt | chimeric, -11/-10/wt | chimeric, -235/wt/-20 |
| 1 | 799 | III | 24 | wt | het, wt/-7 | wt |
| 1 | 800 | IV | 86 | wt | chimeric, -8/-7/wt | chimeric, -5/wt/-4 |
| 1 | 801 | III | 48 | wt | het, wt/-12 | het, wt/-10 |
| 1 | 802 | I | 100 | wt | het, wt/-7 | wt |
| 2 | 803 | III | 48 | wt | chimeric, -19/-11/wt | chimeric, -3/wt/-6/-5 |
| 2 | 804 | 00 | 77 | wt | het, wt/-8 | wt |
| 2 | 805 | III | 24 | wt | het, wt/-11 | wt |
| 2 | 806 | IV | 54 | wt | het, wt/-7 | wt |
| 2 | 807 | IV | 85 | wt | het, wt/-7 | het, wt/-5 |
| 2 | 808 | VI | 93 | wt | chimeric, -13/wt/-7 | het, wt/-117 |
| 2 | 809 | IV | 36 | wt | chimeric, -109/-6/wt | chimeric, wt/-160/-5 |
| 2 | 810 | IV | 52 | wt | chimeric, -189/wt/-6 | het, wt/-189 |
| 2 | 811 | II | 6 | wt | het, wt/-4 | wt |
| 2 | 812 | V | 66 | wt | het, wt/-6 | wt |
| 2 | 813 | II | 33 | wt | het, -16/wt | het, wt/-6 |
| 2 | 814 | IV | 49 | wt | het, -7/wt | het, wt/-9 |
| 2 | 815 | V | 67 | wt | het, wt/-138 | het, wt/-5 |
| 2 | 816 | II | 56 | wt | chimeric, -32/-7/wt | chimeric, -5/-4/wt |
| 2 | 817 | IV | 52 | wt | chimeric, -14/wt/-16 | chimeric, wt/-5/-6 |
| 2 | 818 | III | 98 | wt | chimeric, -54/-7/wt | het, wt/-5 |
| 2 | 819 | V | 92 | wt | chimeric, -40/wt/-7 | het, wt/-5 |
| 2 | 820 | IV | 62 | wt | het, wt/-66 | wt |
| 2 | 821 | IV | 54 | wt | chimeric, wt/-6/-163 | wt |
| 2 | 822 | IV | 81 | wt | chimeric, wt/-8/-7 | chimeric, -6/wt/s1,+17,-15,s1 |
| 2 | 823 | 00 | 77 | wt | chimeric, wt/-8/-7 | het, wt/-5 |
| 2 | 824 | I | 32 | wt | chimeric, wt/-6/-7 | het, wt/-6 |
| 2 | 825 | III | 98 | wt | het, -27/wt | chimeric, wt/-60/-6 |
| 2 | 826 | I | 89 | wt | chimeric, wt/-13/-8 | het, wt/-5 |
| 2 | 827 | II | 15 | wt | chimeric, wt/-11/-7 | chimeric, wt/-12/-5 |
| 2 | 828 | III | 46 | wt | het, wt/-16 | chimeric, wt/-3/-8 |
| 2 | 829 | I | 101 | wt | chimeric, wt/-11/-19 | wt |
| 2 | 830 | III | 98 | wt | het, -7/wt | chimeric, wt/-43/-3 |
| 2 | 831 | III | 22 | wt | biallelic, -12/-7 | homo, -5 |
| 2 | 832 | II | 14 | wt | het, -7/wt | chimeric, wt/-12/-8 |
| 2 | 833 | IV | 59 | wt | chimeric, wt/-7/-26 | chimeric, wt/-9/-5 |
| 2 | 834 | II | 5 | wt | het, -7/wt | het, wt/-26 |
| 2 | 835 | II | 3 | wt | chimeric, wt/-7/-12 | het, wt/-5 |
| 2 | 836 | IV | 61 | wt | het, wt/-7 | wt |
| 2 | 837 | IV | 63 | wt | chimeric, -20/-7/wt | chimeric, -3/-5/wt |
| 2 | 838 | III | 28 | wt | chimeric, -32/wt/-14 | het, -5/wt |
| 2 | 839 | 0 | 68 | wt | wt | wt |
| 2 | 840 | I | 32 | wt | chimeric, wt/-7/-6 | het, -5/wt |
| 2 | 841 | I | 89 | het, wt/-5 | chimeric, -16/wt/-7 | het, wt/-75 |
| 2 | 842 | 0 | 68 | wt | chimeric, wt/-9/-13 | chimeric, wt/-5/-9 |
| 2 | 843 | IV | 35 | wt | wt | wt |
| 2 | 844 | III | 47 | het, wt/-6 | chimeric, -186/wt/-12 | chimeric, wt/-186/-49 |
| 2 | 845 | 00 | 77 | wt | chimeric, wt/-13/-7/-8/-11 | wt |
| 2 | 846 | I | 32 | wt | het, -7/wt | het, wt/-56 |
| 2 | 847 | 0 | 88 | wt | chimeric, -7/-11/-6/-8 | chimeric, wt/-5/-6 |
| 2 | 848 | III | 48 | wt | het, -7/wt | chimeric, wt/-7/-6 |
| 2 | 849 | II | 80 | wt | het, wt/-7 | wt |
| 2 | 850 | IV | 84 | wt | wt | wt |
| 2 | 851 | IV | 35 | wt | het, wt/-13 | het, wt/-7 |
| 2 | 852 | III | 45 | wt | chimeric, wt/-8/-6 | chimeric, wt/-21/-41 |
| 2 | 853 | IV | 58 | chimeric, wt/-422/-3 | chimeric, -422/wt/-19 | chimeric, wt/-422/-16 |
| 2 | 854 | IV | 36 | wt | chimeric, -52/-13/wt | het, -5/wt |
| 2 | 855 | 0 | 68 | wt | chimeric, wt/-16/-7 | het, -5/wt |
| 2 | 856 | I | 10 | wt | chimeric, -72/wt/-8,s2 | chimeric, -6/wt/-79 |
| 2 | 857 | 0 | 41 | wt | chimeric, wt/-7/-24 | het, wt/-5 |
| 2 | 858 | III | 25 | wt | het, -7/wt | chimeric, -5/-6/wt |
| 2 | 859 | II | 3 | wt | chimeric, -35/wt/-39 | het, wt/-5 |
| 2 | 860 | II | 38 | wt | chimeric, -2/wt/-7 | wt |
| 2 | 861 | II | 56 | het, wt/-6 | chimeric, -8/wt/-30 | het, -5/wt |
| 2 | 862 | III | 46 | wt | chimeric, wt/-11/-7 | het, wt/-5 |
| 2 | 863 | II | 3 | wt | chimeric, wt/-7/s1,-10 | chimeric, wt/-5/-3 |
| 2 | 864 | II | 7 | wt | chimeric, -13/wt/-7 | het, wt/-10 |
| 2 | 865 | II | 13 | wt | chimeric, -15/-8/wt | wt |
| 2 | 866 | II | 80 | wt | chimeric, -7/wt/-7 | het, wt/-5 |
| 2 | 867 | III | 26 | wt | chimeric, wt/-5/-10 | het, wt/-5 |
| 2 | 868 | V | 91 | wt | chimeric, -34/wt/-8 | wt |
| 2 | 869 | IV | 54 | wt | chimeric, wt/-7/-38 | wt |
| 2 | 870 | III | 50 | wt | het, wt/-7 | het, wt/-6 |
| 2 | 871 | 0 | 41 | wt | het, wt/-6 | het, wt/-5 |
| 2 | 872 | I | 9 | wt | het, -46/wt | wt |
| 2 | 873 | IV | 52 | wt | het, wt/-7 | het, wt/-5 |
| 2 | 874 | 0 | 40 | wt | chimeric, -7/-8/wt | chimeric, -8/-5/wt |
| 2 | 875 | I | 89 | wt | het, wt/s1,+36,s3,s3 | wt |
| 2 | 876 | IV | 83 | wt | chimeric, -56/-21/wt | wt |
| 2 | 877 | I | 89 | wt | chimeric, wt/-7/-8 | wt |
| 2 | 878 | IV | 31 | wt | wt | wt |
| 2 | 879 | II | 12 | wt | chimeric, -5/-7/wt | chimeric, wt/-5/-5 |
| 2 | 880 | IV | 55 | wt | wt | wt |
| 2 | 881 | V | 60 | wt | het, -8/wt | wt |
| 2 | 882 | 00 | 78 | het, wt/-6 | chimeric, wt/-11/-33 | chimeric, wt/-5/-16 |
| 2 | 883 | III | 51 | wt | chimeric, -11/wt/-7 | wt |
| 2 | 884 | I | 89 | wt | biallelic, -7/-9 | biallelic, -112/-5 |
| 2 | 885 | IV | 54 | wt | chimeric, wt/-5/-6 | het, wt/-14 |
| 2 | 886 | IV | 61 | wt | chimeric, -57/wt/-11 | wt |
| 2 | 887 | IV | 35 | wt | chimeric, -5/-11/wt | wt |
| 2 | 888 | IV | 31 | wt | het, wt/-7 | wt |
| 2 | 889 | III | 19 | wt | chimeric, -13/-9/wt | chimeric, -5/wt/-59 |
| 2 | 890 | VI | 93 | wt | chimeric, -7/-19/wt | chimeric, -6/-5/wt |
| 2 | 891 | IV | 55 | wt | chimeric, -67/-6/wt | chimeric, -5/-14/wt |
| 2 | 892 | I | 10 | het, wt/-510 | chimeric, -510/-8/wt | chimeric, -510/-5/wt |
| 2 | 893 | I | 89 | wt | het, wt/-7 | wt |
| 2 | 894 | II | 15 | wt | chimeric, wt/-10/-6 | het, wt/-5 |
| 2 | 895 | V | 92 | wt | chimeric, -13/wt/-7 | het, wt/-8 |
| 2 | 896 | IV | 81 | wt | het, -6/wt | wt |
| 2 | 897 | III | 45 | wt | chimeric, -32/-9/wt | het, wt/-5 |
| 2 | 898 | IV | 84 | wt | het, wt/-7 | wt |
| 2 | 899 | III | 50 | wt | chimeric, -13/wt/-17 | chimeric, wt/-6/-26 |
| 2 | 900 | V | 65 | wt | chimeric, -192/-10/wt | het, wt/-192 |
| 2 | 901 | II | 6 | wt | chimeric, -11/-8/wt | het, wt/-17 |
| 2 | 902 | I | 89 | wt | het, -7/wt | het, wt/-5 |
| 2 | 903 | II | 43 | wt | chimeric, -185/wt/-7 | het, wt/-185 |
| 2 | 904 | III | 25 | wt | het, wt/-7 | het, wt/-5 |
| 2 | 905 | II | 3 | wt | wt | wt |
| 2 | 906 | III | 98 | wt | het, -32/wt | wt |
| 2 | 907 | II | 12 | wt | chimeric, -8/wt/-6 | wt |
| 2 | 908 | II | 80 | wt | chimeric, -11/-16/-7/-8 | het, wt/-5 |
| 2 | 909 | IV | 52 | wt | chimeric, wt/-163/-31 | wt |
| 2 | 910 | III | 26 | wt | chimeric, -8/-7/wt | wt |
| 2 | 911 | V | 66 | wt | het, wt/-8 | het, wt/-21 |
| 2 | 912 | V | 67 | wt | het, -7/wt | het, wt/-3 |
| 2 | 913 | III | 50 | wt | het, wt/-9 | wt |
| 2 | 914 | II | 43 | wt | chimeric, wt/s1,-43/-7 | wt |
| 2 | 915 | III | 45 | wt | het, wt/-7 | het, wt/-5 |
| 2 | 916 | V | 65 | wt | chimeric, -6/wt/-7 | chimeric, wt/-9/-11/-9 |
| 2 | 917 | I | 102 | wt | homo, -7 | het, -5/wt |
| 2 | 918 | II | 80 | wt | het, -7/wt | het, wt/-5 |
| 2 | 919 | II | 38 | wt | chimeric, -8/-13/-7/s1,-7 | chimeric, -9/-5/wt/-6 |
| 2 | 920 | VII | 95 | wt | chimeric, -18/-9/wt | chimeric, -5/-11/wt |
| 2 | 921 | III | 25 | wt | chimeric, -26/-11/wt | wt |
| 2 | 922 | II | 80 | wt | chimeric, wt/-7/-17 | wt |
| 2 | 923 | I | 102 | wt | het, wt/-7 | wt |
| 2 | 924 | IV | 54 | wt | chimeric, -185/-13/wt | chimeric, -185/wt/-5 |
| 2 | 925 | I | 74 | wt | chimeric, s1,-8/-16/wt | het, wt/-5 |
| 2 | 926 | II | 15 | wt | het, wt/-7 | het, wt/-7 |
| 2 | 927 | II | 56 | wt | chimeric, wt/-16/-22 | het, wt/-5 |
| 2 | 928 | IV | 86 | wt | het, wt/s1,s1,-4 | wt |
| 2 | 929 | II | 6 | wt | het, -7/wt | het, wt/-5 |
| 2 | 930 | I | 75 | wt | chimeric, -32/wt/-7 | het, wt/-6 |
| 2 | 931 | II | 38 | wt | chimeric, wt/-7/-6 | chimeric, wt/-3/-9 |
| 2 | 932 | V | 92 | het, wt/-6 | chimeric, s4,-22/-13/wt | het, wt/-5 |
| 2 | 933 | II | 33 | wt | chimeric, wt/-7/-7 | het, wt/-49 |
| 2 | 934 | VI | 93 | wt | chimeric, -11/-7/wt | chimeric, -5/-10/wt |
| 2 | 935 | IV | 86 | wt | chimeric, wt/-6/-7 | wt |
| 2 | 936 | IV | 62 | wt | chimeric, -669/wt/-11 | het, wt/-669 |
| 2 | 937 | V | 60 | wt | chimeric, wt/-16/-7 | chimeric, wt/-5/-5 |
| 2 | 938 | I | 11 | wt | chimeric, -24/wt/-7 | chimeric, wt/-162/-9 |
| 2 | 939 | V | 66 | wt | chimeric, wt/-7/-9 | het, wt/-111 |
| 2 | 940 | IV | 52 | wt | chimeric, wt/-7/-132/s1,s1,-19 | het, wt/-5 |
| 2 | 941 | II | 56 | wt | wt | wt |
| 2 | 942 | III | 25 | wt | het, wt/-8 | het, wt/-5 |
| 2 | 943 | II | 43 | wt | chimeric, wt/-16/-7 | wt |
| 2 | 944 | 0 | 41 | wt | chimeric, -13/wt/-86 | wt |
| 2 | 945 | II | 15 | wt | chimeric, wt/-12/-11 | het, wt/-8 |
| 2 | 946 | IV | 86 | wt | chimeric, -11/-7/wt | het, wt/-10 |
| 2 | 947 | 0 | 41 | wt | chimeric, -82/-17/wt | wt |
| 2 | 948 | IV | 58 | wt | chimeric, -65/wt/-20 | het, wt/-27 |
| 2 | 949 | IV | 64 | wt | het, -16/wt | het, wt/-5 |
| 2 | 950 | V | 67 | wt | chimeric, wt/-6/-7 | wt |
| 2 | 951 | IV | 59 | wt | chimeric, -39/wt/-7 | wt |
| 2 | 952 | IV | 79 | wt | chimeric, wt/-7/-7 | het, wt/-9 |
| 2 | 953 | V | 92 | wt | chimeric, -85/wt/-6 | het, wt/-5 |
| 2 | 954 | I | 11 | wt | het, wt/-7 | wt |
| 2 | 955 | III | 45 | wt | chimeric, wt/-6/-10 | het, wt/-5 |
| 2 | 956 | 00 | 78 | wt | wt | wt |
| 2 | 957 | I | 89 | wt | chimeric, -18/-11/wt | chimeric, -5/-10/wt |
| 2 | 958 | IV | 36 | wt | chimeric, wt/-15/-8 | wt |
| 2 | 959 | II | 38 | het, wt/-338 | chimeric, -338/-13/-9/wt | chimeric, -338/wt/-5 |
| 2 | 960 | VII | 95 | wt | chimeric, -64/s1,-8/wt | wt |
| 2 | 961 | IV | 55 | wt | chimeric, -7/-10/wt | het, wt/-5 |
| 2 | 962 | 0 | 37 | wt | chimeric, -3/wt/-7 | wt |
| 2 | 963 | IV | 64 | wt | het, -16/wt | wt |
| 2 | 964 | 0 | 88 | wt | chimeric, -8/wt/-10 | het, wt/-5 |
| 2 | 965 | III | 26 | wt | chimeric, -20/-12/-6/wt | wt |
| 2 | 966 | I | 100 | wt | chimeric, -432/-9/wt | chimeric, -432/-34/wt |
| 2 | 967 | IV | 58 | wt | het, wt/-148 | wt |
| 2 | 968 | V | 91 | wt | chimeric, -44/wt/-7 | chimeric, wt/-27/-5 |
| 2 | 969 | VII | 95 | wt | het, -185/wt | het, -185/wt |
| 2 | 970 | II | 13 | wt | het, wt/-7 | wt |
| 2 | 971 | IV | 35 | wt | het, -7/wt | wt |
| 2 | 972 | V | 91 | het, wt/-3 | chimeric, -185/-19/wt | chimeric, -185/-6/wt |
| 2 | 973 | 00 | 90 | wt | chimeric, -16/wt/-13 | chimeric, wt/-5/-6 |
| 2 | 974 | V | 67 | wt | chimeric, wt/-7/-9 | het, wt/-17 |
| 2 | 975 | III | 46 | wt | chimeric, wt/-10/-7 | wt |
| 2 | 976 | IV | 84 | wt | wt | wt |
| 2 | 977 | IV | 79 | wt | chimeric, -6/wt/-55 | wt |
| 2 | 978 | IV | 62 | wt | chimeric, wt/-11/-13 | chimeric, wt/-5/-24 |
| 2 | 979 | IV | 34 | wt | chimeric, wt/-11/-12 | wt |
| 2 | 980 | I | 89 | wt | wt | wt |
| 2 | 981 | I | 32 | wt | chimeric, -193/-8/wt | chimeric, -193/-5/wt |
| 2 | 982 | IV | 52 | wt | het, -7/wt | het, wt/-9 |
| 2 | 983 | III | 47 | wt | het, wt/-7 | wt |
| 2 | 984 | III | 46 | wt | wt | wt |
| 2 | 985 | IV | 36 | wt | chimeric, -6/-7/wt | chimeric, -27/-53/wt |
| 2 | 986 | 00 | 90 | wt | chimeric, -158/wt/-20 | wt |
| 2 | 987 | I | 73 | wt | chimeric, wt/-6/-7 | het, wt/-5 |
| 2 | 988 | IV | 35 | wt | chimeric, wt/-6/-7 | het, wt/-11 |
| 2 | 989 | I | 74 | wt | chimeric, wt/-10/-7 | wt |
| 2 | 990 | 00 | 77 | wt | chimeric, -11/-10/wt | het, -5/wt |
| 2 | 991 | IV | 84 | wt | chimeric, wt/-5/-7 | het, wt/-5 |
| 2 | 992 | III | 29 | wt | biallelic, s1,-11/-8 | het, -656/wt |
| 2 | 993 | II | 6 | wt | chimeric, -5/wt/-13 | het, -5/wt |
| 2 | 994 | I | 32 | wt | chimeric, wt/-13/s1,-6 | chimeric, wt/-80/-5 |
| 2 | 995 | III | 46 | wt | wt | wt |
| 2 | 996 | III | 98 | wt | chimeric, -7/-6/wt | het, wt/-5 |
| 2 | 997 | 0 | 68 | wt | chimeric, wt/-38/-16/-6/-4 | wt |
| 2 | 998 | III | 21 | chimeric, -13/-202/wt | chimeric, -202/-38/wt | het, -5/wt |
| 2 | 999 | IV | 57 | wt | wt | wt |
| 2 | 1000 | II | 15 | wt | chimeric, -199/-7/wt | chimeric, -199/-6/wt |
| 2 | 1001 | IV | 84 | wt | het, -37/wt | wt |
| 2 | 1002 | II | 3 | wt | het, wt/-7 | het, wt/-5 |
| 2 | 1003 | I | 11 | wt | chimeric, -23/-7/wt | wt |
| 2 | 1004 | III | 26 | wt | het, wt/-7 | wt |
| 2 | 1005 | I | 10 | wt | het, -7/wt | chimeric, -5/-7/wt |
| 2 | 1006 | II | 15 | wt | chimeric, wt/-7/-7 | het, wt/-5 |
| 2 | 1007 | IV | 35 | wt | chimeric, wt/-20/-7 | het, wt/-13 |
| 2 | 1008 | III | 25 | wt | homo, s1,-6 | het, -116/wt |
| 2 | 1009 | V | 91 | wt | het, wt/-7 | wt |
| 2 | 1010 | II | 80 | wt | chimeric, wt/s1,-146/-7 | het, wt/-5 |
| 2 | 1011 | III | 20 | het, -167/wt | biallelic, -167/-6 | biallelic, -6/-8 |
| 2 | 1012 | III | 21 | wt | chimeric, wt/-7/-17/-8 | chimeric, wt/-6/-17 |
| 2 | 1013 | I | 100 | wt | chimeric, wt/-8/-15 | het, wt/-27 |
| 2 | 1014 | IV | 84 | wt | het, wt/-7 | wt |
| 2 | 1015 | I | 102 | wt | chimeric, wt/-11/-8 | wt |
| 2 | 1016 | II | 3 | het, wt/-5 | chimeric, wt/-10/-8 | chimeric, wt/-13/-20 |
| 2 | 1017 | III | 30 | wt | chimeric, -16/-10/wt | het, wt/-5 |
| 2 | 1018 | IV | 52 | wt | chimeric, wt/-6/-7 | chimeric, wt/-214/-27 |
| 2 | 1019 | IV | 61 | wt | chimeric, wt/-11/-6 | het, wt/-3 |
| 2 | 1020 | II | 80 | wt | het, -17/wt | wt |
| 2 | 1021 | IV | 84 | wt | chimeric, -17/-73/wt | chimeric, -5/wt/-56 |
| 2 | 1022 | III | 50 | wt | chimeric, wt/-9/-8 | chimeric, wt/-7/-5 |
| 2 | 1023 | III | 25 | wt | chimeric, wt/-29/-10 | wt |
| 2 | 1024 | 0 | 68 | wt | wt | wt |
| 2 | 1025 | III | 87 | wt | chimeric, -7/wt/-6 | chimeric, wt/-5/-90 |
| 2 | 1026 | IV | 57 | wt | chimeric, -3/wt/-10 | wt |
| 2 | 1027 | II | 7 | wt | chimeric, -12/wt/-6 | chimeric, wt/-5/-9/-5 |
| 2 | 1028 | IV | 62 | wt | het, -27/wt | het, -5/wt |
| 2 | 1029 | IV | 64 | wt | chimeric, wt/-13/-7 | chimeric, wt/-9/-5 |
| 2 | 1030 | II | 3 | wt | chimeric, wt/s1,-8/-18 | het, wt/-4 |
| 2 | 1031 | IV | 58 | wt | chimeric, -63/wt/s4,s1,+18 | het, wt/-5 |
| 2 | 1032 | IV | 79 | wt | wt | wt |
| 2 | 1033 | III | 28 | wt | chimeric, -7/-15/-13 | chimeric, -10/-26/wt |
| 2 | 1034 | II | 13 | wt | chimeric, -11/wt/-6 | chimeric, wt/-4/-8 |
| 2 | 1035 | 0 | 68 | wt | het, wt/-7 | het, wt/-5 |
| 2 | 1036 | II | 6 | wt | chimeric, wt/-10/-13 | wt |
| 2 | 1037 | 00 | 90 | wt | chimeric, -636/wt/-7 | het, wt/-636 |
| 2 | 1038 | II | 6 | wt | het, wt/-7 | wt |
| 2 | 1039 | IV | 61 | wt | chimeric, wt/-11/-18 | chimeric, wt/-21/-15/-5 |
| 2 | 1040 | IV | 52 | wt | chimeric, wt/-8/-28 | het, wt/-3 |
| 2 | 1041 | 0 | 37 | wt | het, -6/wt | chimeric, -12/-32/wt |
| 2 | 1042 | III | 48 | wt | wt | wt |
| 2 | 1043 | II | 38 | wt | chimeric, wt/-189/s1,-10 | chimeric, wt/-189/-6 |
| 2 | 1044 | I | 103 | wt | chimeric, wt/-11/-13 | wt |
| 2 | 1045 | V | 65 | wt | chimeric, -5/wt/-29 | chimeric, -5/wt/-11 |
| 2 | 1046 | I | 32 | het, wt/-32 | chimeric, -11/wt/-9 | chimeric, -3/-5/wt |
| 2 | 1047 | IV | 57 | wt | het, wt/-7 | het, wt/-5 |
| 2 | 1048 | III | 48 | wt | wt | wt |
| 2 | 1049 | I | 102 | wt | chimeric, -60/wt/-16 | het, wt/-5 |
| 2 | 1050 | II | 15 | wt | chimeric, -13/wt/-8 | chimeric, wt/-5/-6 |
| 2 | 1051 | II | 15 | wt | het, wt/-9 | wt |
| 2 | 1052 | IV | 36 | het, wt/-5 | chimeric, wt/-7/-540 | chimeric, wt/-5/-540 |
| 2 | 1053 | IV | 62 | wt | chimeric, wt/-44/-7 | chimeric, wt/-41/-5 |
| 2 | 1054 | 0 | 70 | wt | chimeric, wt/-6/-31 | wt |
| 2 | 1055 | III | 24 | wt | het, wt/-7 | wt |
| 2 | 1056 | IV | 64 | chimeric, wt/-6/-3 | chimeric, wt/-10/-6 | chimeric, wt/-5/-53 |
| 2 | 1057 | IV | 81 | wt | chimeric, -13/-11/wt | chimeric, wt/-115/-97 |
| 2 | 1058 | 00 | 90 | wt | chimeric, wt/-14/-13 | het, wt/-3 |
| 2 | 1059 | II | 16 | het, wt/-12 | chimeric, wt/-48/-17/-7 | chimeric, wt/-28/-8 |
| 2 | 1060 | IV | 84 | wt | het, wt/-7 | wt |
| 2 | 1061 | I | 103 | wt | het, -7/wt | chimeric, wt/-5/-3 |
| 2 | 1062 | III | 98 | wt | het, -194/wt | het, -194/wt |
| 2 | 1063 | 0 | 37 | wt | het, wt/-19 | wt |
| 2 | 1064 | III | 18 | wt | chimeric, wt/-13/-10 | het, wt/-191 |
| 2 | 1065 | III | 48 | het, wt/-4 | chimeric, -13/-6/wt | chimeric, wt/-43/-6/-27 |
| 2 | 1066 | IV | 57 | wt | chimeric, -64/-10/-10 | wt |
| 2 | 1067 | I | 101 | wt | chimeric, -7/-71/-69 | biallelic, -5/-5 |
| 2 | 1068 | II | 7 | wt | chimeric, wt/-16/-11/-609 | wt |
| 2 | 1069 | III | 50 | wt | chimeric, wt/-16/-6 | het, wt/-5 |
| 2 | 1070 | III | 50 | wt | chimeric, -11/wt/-7 | chimeric, wt/-14/-5 |
| 2 | 1071 | 0 | 37 | wt | chimeric, wt/-5/-7 | chimeric, wt/-21/-7 |
| 2 | 1072 | 0 | 68 | wt | het, -7/wt | chimeric, wt/-5/-19 |
| 2 | 1073 | II | 38 | wt | het, -7/wt | het, wt/-5 |
| 2 | 1074 | IV | 55 | wt | chimeric, wt/-7/-5 | wt |
| 2 | 1075 | III | 26 | wt | wt | wt |
| 2 | 1076 | III | 47 | wt | chimeric, wt/-3/s1,-10 | het, wt/-5 |
| 2 | 1077 | II | 7 | wt | het, -7/wt | wt |
| 2 | 1078 | I | 89 | wt | chimeric, -208/-26/wt | het, wt/-208 |
| 2 | 1079 | III | 25 | wt | wt | wt |
| 2 | 1080 | I | 74 | wt | chimeric, -9/-7/wt | wt |
| 2 | 1081 | II | 7 | wt | chimeric, wt/-44/-7 | wt |
| 2 | 1082 | III | 53 | wt | chimeric, -5/-12/wt | wt |
| 2 | 1083 | III | 47 | wt | chimeric, -64/wt/-12 | chimeric, wt/-11/-9 |
| 2 | 1084 | II | 7 | wt | chimeric, -7/-11/-8 | het, -5/wt |
| 2 | 1085 | 0 | 39 | wt | chimeric, -15/wt/-7 | het, wt/-5 |
| 2 | 1086 | IV | 62 | wt | chimeric, -32/-7/-8 | wt |
| 2 | 1087 | V | 92 | wt | chimeric, wt/-9/-6 | het, wt/-6 |
| 2 | 1088 | III | 48 | wt | chimeric, -37/wt/-15 | chimeric, wt/-9/-5/-45 |
| 2 | 1089 | III | 26 | wt | chimeric, wt/-8/-11 | wt |
| 2 | 1090 | 00 | 78 | wt | chimeric, -98/-11/wt | wt |
| 2 | 1091 | IV | 86 | wt | chimeric, -12/-6/wt | het, wt/-5 |
| 2 | 1092 | II | 15 | wt | wt | wt |
| 2 | 1093 | IV | 63 | wt | chimeric, wt/-8/-6 | chimeric, wt/-9/-43 |
| 2 | 1094 | 00 | 77 | wt | het, -7/wt | het, wt/-15 |
| 2 | 1095 | II | 7 | wt | chimeric, -17/-7/wt | het, wt/-5 |
| 2 | 1096 | 00 | 77 | wt | chimeric, wt/-8/-19/-7/-19 | chimeric, wt/-6/-9/-4 |
| 2 | 1097 | III | 50 | wt | chimeric, wt/s1,-7/-9 | chimeric, wt/-8/-5 |
| 2 | 1098 | V | 92 | wt | het, wt/-7 | chimeric, wt/-4/-5 |
| 2 | 1099 | IV | 54 | wt | chimeric, wt/-6/-41 | wt |
| 2 | 1100 | IV | 59 | wt | chimeric, -10/-7/wt | chimeric, -3/-6/wt |
| 2 | 1101 | IV | 36 | wt | het, -7/wt | het, wt/-5 |
| 2 | 1102 | III | 53 | wt | het, wt/-13 | wt |
| 2 | 1103 | V | 92 | wt | chimeric, -7/-106/wt | chimeric, wt/-6/-5 |
| 2 | 1104 | IV | 58 | wt | chimeric, wt/-7/-12 | wt |
| 2 | 1105 | II | 3 | wt | chimeric, wt/s1,-10/-53 | wt |
| 2 | 1106 | IV | 55 | wt | chimeric, -71/wt/-11 | wt |
| 2 | 1107 | V | 67 | wt | het, wt/-10 | wt |
| 2 | 1108 | IV | 34 | wt | chimeric, -10/wt/-7 | het, wt/-10 |
| 2 | 1109 | V | 92 | wt | chimeric, wt/-19/-19 | chimeric, wt/-3/-6 |
| 2 | 1110 | IV | 34 | wt | wt | wt |
| 2 | 1111 | IV | 63 | wt | chimeric, -203/wt/-11 | het, wt/-203 |
| 2 | 1112 | I | 100 | wt | chimeric, -14/wt/-7/-5 | het, wt/-5 |
| 2 | 1113 | I | 10 | wt | chimeric, -75/-6/wt | wt |
| 2 | 1114 | II | 43 | wt | chimeric, -53/wt/-1/-122/-75 | het, wt/-7 |
| 2 | 1115 | II | 56 | wt | chimeric, -17/-7/wt | het, wt/-5 |
| 2 | 1116 | V | 60 | wt | chimeric, wt/-16/-13 | het, wt/-9 |
| 2 | 1117 | III | 47 | wt | chimeric, -11/wt/-17 | chimeric, wt/-14/-5 |
| 2 | 1118 | V | 92 | wt | chimeric, wt/-28/-6 | het, wt/-5 |
| 2 | 1119 | IV | 31 | wt | chimeric, wt/-10/-6 | het, wt/-5 |
| 2 | 1120 | I | 42 | wt | het, wt/-8 | wt |
| 2 | 1121 | III | 23 | wt | het, wt/-7 | wt |
| 2 | 1122 | II | 7 | wt | chimeric, -16/-7/wt | chimeric, wt/-5/-5 |
| 2 | 1123 | II | 6 | wt | chimeric, -7/wt/-6 | chimeric, wt/-18/-5 |
| 2 | 1124 | V | 60 | wt | het, wt/-7 | wt |
| 2 | 1125 | II | 80 | wt | chimeric, wt/-37/-25 | het, wt/-6 |
| 2 | 1126 | 00 | 77 | wt | het, -7/wt | chimeric, wt/-6/-10 |
| 2 | 1127 | II | 80 | wt | het, -197/wt | het, wt/-197 |
| 2 | 1128 | 00 | 90 | wt | chimeric, -11/wt/-5 | het, -5/wt |
| 2 | 1129 | II | 38 | wt | chimeric, wt/-26/-15 | chimeric, wt/-8/-5 |
| 2 | 1130 | IV | 59 | wt | wt | wt |
| 2 | 1131 | 0 | 70 | wt | het, wt/-7 | wt |
| 2 | 1132 | II | 3 | wt | chimeric, -41/-7/wt | chimeric, -5/wt/-26 |
| 2 | 1133 | II | 3 | wt | chimeric, wt/-16/-25 | wt |
| 2 | 1134 | I | 74 | wt | chimeric, -13/-7/wt | het, wt/-5 |
| 2 | 1135 | II | 15 | wt | chimeric, wt/-8/s1,-10 | chimeric, wt/-10/-5 |
| 2 | 1136 | I | 75 | wt | chimeric, wt/-7/-14 | wt |
| 2 | 1137 | III | 98 | wt | chimeric, -12/-19/wt | chimeric, -77/wt/-7 |
| 2 | 1138 | V | 92 | wt | chimeric, -5/-7/wt | chimeric, -92/-6/wt |
| 2 | 1139 | 00 | 76 | wt | het, wt/-7 | wt |
| 2 | 1140 | III | 30 | wt | chimeric, -7/wt/-8 | het, wt/-5 |
| 2 | 1141 | 0 | 41 | wt | wt | wt |
| 2 | 1142 | III | 48 | wt | chimeric, -131/wt/-6 | wt |
| 2 | 1143 | II | 7 | wt | chimeric, wt/s1,-8/-9 | wt |
| 2 | 1144 | I | 102 | het, wt/-10 | chimeric, -13/-6/-7/-30 | chimeric, wt/-9/-6 |
| 2 | 1145 | I | 42 | wt | wt | wt |
| 2 | 1146 | 0 | 72 | wt | chimeric, -82/-7/wt | het, wt/-5 |
| 2 | 1147 | II | 3 | wt | chimeric, wt/-7/-17 | wt |
| 2 | 1148 | V | 67 | chimeric, -378/-34/wt | het, -13/wt | het, wt/-36 |
| 2 | 1149 | II | 3 | wt | chimeric, -20/-6/wt | wt |
| 2 | 1150 | I | 74 | wt | wt | wt |
| 2 | 1151 | I | 73 | wt | chimeric, -20/-7/wt | het, wt/-5 |
| 2 | 1152 | II | 12 | wt | chimeric, -67/-189/wt | chimeric, -189/wt/-9 |
| 2 | 1153 | II | 33 | wt | chimeric, wt/-7/-9 | het, wt/-5 |
| 2 | 1154 | II | 16 | wt | het, wt/-7 | het, wt/-5 |
| 2 | 1155 | III | 98 | wt | het, wt/-15 | wt |
| 2 | 1156 | V | 65 | wt | het, -26/wt | wt |
| 2 | 1157 | IV | 86 | wt | wt | wt |
| 2 | 1158 | IV | 36 | wt | het, wt/-11 | wt |
| 2 | 1159 | V | 67 | wt | het, wt/-7 | het, wt/-5 |
| 2 | 1160 | IV | 55 | wt | chimeric, -118/wt/-7 | chimeric, wt/-9/-9 |
| 2 | 1161 | I | 89 | wt | chimeric, wt/-7/-14 | het, -5/wt |
| 2 | 1162 | IV | 79 | wt | chimeric, wt/-7/-18 | wt |
| 2 | 1163 | IV | 85 | wt | chimeric, wt/-132/-7 | wt |
| 2 | 1164 | III | 98 | wt | het, wt/-7 | wt |
| 2 | 1165 | IV | 79 | wt | wt | wt |
| 2 | 1166 | I | 100 | wt | het, wt/-25 | chimeric, wt/-5/-26 |
| 2 | 1167 | V | 67 | wt | wt | wt |
| 2 | 1168 | III | 28 | wt | het, -7/wt | het, wt/-5 |
| 2 | 1169 | II | 15 | wt | het, wt/-3 | wt |
| 2 | 1170 | III | 26 | wt | chimeric, -109/-28/-8/-37 | wt |
| 2 | 1171 | V | 92 | wt | chimeric, wt/-11/-8 | wt |
| 2 | 1172 | IV | 58 | wt | chimeric, wt/s1,s2,s1,s2/-14,s1,s2 | het, wt/-5 |
| 2 | 1173 | I | 101 | wt | chimeric, wt/-7/-53 | het, wt/-57 |
| 2 | 1174 | I | 32 | wt | het, wt/-13 | het, wt/-6 |
| 2 | 1175 | II | 3 | wt | biallelic, -22/-6 | chimeric, wt/-6/-5 |
| 2 | 1176 | IV | 58 | wt | chimeric, -151/wt/s1,-12 | wt |
| 2 | 1177 | IV | 58 | het, wt/-6 | chimeric, wt/-18/-7 | chimeric, wt/-10/-21 |
| 2 | 1178 | IV | 61 | wt | chimeric, wt/-10/-9 | chimeric, wt/-5/-5 |
| 2 | 1179 | V | 67 | wt | chimeric, wt/-7/-6 | chimeric, wt/-5/-5 |
| 2 | 1180 | II | 7 | wt | het, wt/-10,s2 | wt |
| 2 | 1181 | III | 98 | wt | chimeric, wt/-225/-7 | chimeric, wt/-5/-225 |
| 2 | 1182 | VII | 95 | wt | het, -7/wt | het, -5/wt |
| 2 | 1183 | I | 102 | wt | het, -7/wt | het, -14/wt |
| 2 | 1184 | 0 | 72 | wt | chimeric, wt/s1,-52/-1/-38 | wt |
| 2 | 1185 | I | 42 | wt | wt | wt |
| 2 | 1186 | III | 25 | wt | het, -186/wt | het, wt/-186 |
| 2 | 1187 | IV | 58 | wt | biallelic, -7/-9 | biallelic, -5/-6 |
| 2 | 1188 | III | 28 | wt | chimeric, wt/-7/-6 | wt |
| 2 | 1189 | 00 | 90 | wt | wt | wt |
| 2 | 1190 | IV | 55 | wt | chimeric, wt/-11/-7 | wt |
| 2 | 1191 | IV | 36 | wt | chimeric, wt/-7/-10 | chimeric, wt/-6/-19 |
| 2 | 1192 | 00 | 90 | wt | chimeric, -7/-8/wt | chimeric, wt/-659/-5 |
| 2 | 1193 | III | 50 | wt | chimeric, -50/-8/wt | het, wt/-5 |
| 2 | 1194 | IV | 61 | wt | chimeric, wt/-8/-7 | het, wt/-6 |
| 2 | 1195 | III | 19 | wt | chimeric, wt/-10/-178/-6 | wt |
| 2 | 1196 | III | 27 | wt | chimeric, -8/-6/-7/-4 | chimeric, wt/-5/-7 |
| 2 | 1197 | V | 92 | wt | biallelic, -11/-7 | biallelic, -5/-8 |
| 2 | 1198 | III | 46 | wt | wt | wt |
| 2 | 1199 | III | 25 | het, wt/-5 | chimeric, wt/-6/-31 | wt |
| 2 | 1200 | 0 | 70 | wt | het, wt/-13 | wt |
| 2 | 1201 | II | 3 | het, wt/-6 | chimeric, -180/wt/-7 | het, wt/-180 |
| 2 | 1202 | III | 30 | wt | wt | wt |
| 2 | 1203 | IV | 62 | wt | chimeric, wt/-7/-10 | het, wt/-15 |
| 2 | 1204 | II | 33 | wt | het, -6/wt | wt |
| 2 | 1205 | IV | 36 | wt | chimeric, wt/-8/-7 | wt |
| 2 | 1206 | 0 | 88 | wt | het, wt/-9 | wt |
| 2 | 1207 | III | 22 | wt | chimeric, -18/-6/-13/wt | chimeric, wt/s1/-9 |
| 2 | 1208 | 00 | 90 | wt | chimeric, wt/-53/-7/-21 | wt |
| 2 | 1209 | IV | 55 | wt | chimeric, wt/-7/-13 | wt |
| 2 | 1210 | IV | 81 | wt | wt | wt |
| 2 | 1211 | I | 42 | wt | chimeric, s2,-30/-8/-403 | chimeric, wt/-403/-5 |
| 2 | 1212 | IV | 81 | wt | chimeric, -13/-13/wt | chimeric, wt/-3/-5 |
| 2 | 1213 | II | 80 | wt | het, wt/-7 | het, wt/-5 |
| 2 | 1214 | II | 3 | wt | chimeric, -8/wt/-6 | wt |
| 2 | 1215 | II | 43 | wt | wt | wt |
| 2 | 1216 | III | 45 | wt | chimeric, wt/-26/-8 | het, wt/-5 |
| 2 | 1217 | III | 48 | het, wt/-509 | chimeric, -509/wt/-7 | wt |
| 2 | 1218 | III | 26 | wt | het, -11/wt | het, -5/wt |
| 2 | 1219 | II | 15 | wt | chimeric, wt/-68/-105 | het, wt/-5 |
| 2 | 1220 | III | 97 | wt | het, wt/-7 | wt |
| 2 | 1221 | 0 | 37 | wt | chimeric, wt/-6/-7 | wt |
| 2 | 1222 | IV | 34 | het, wt/-2 | chimeric, wt/-124/-7 | het, wt/-6 |
| 2 | 1223 | II | 80 | wt | chimeric, -44/wt/-7 | chimeric, wt/-5/-5 |
| 2 | 1224 | V | 92 | wt | het, wt/-7 | wt |
| 2 | 1225 | IV | 58 | wt | chimeric, wt/-8/-7 | wt |
| 2 | 1226 | IV | 55 | wt | chimeric, wt/-8/-7 | chimeric, wt/-6/-7 |
| 2 | 1227 | II | 56 | wt | chimeric, wt/-9/-10 | chimeric, wt/-98/-29 |
| 2 | 1228 | II | 6 | wt | het, wt/-7 | wt |
| 2 | 1229 | V | 67 | wt | chimeric, wt/-185/-50/-8/-6 | het, wt/-185 |
| 2 | 1230 | IV | 55 | wt | het, -17/wt | het, -3/wt |
| 2 | 1231 | II | 80 | het, wt/-640 | chimeric, -640/-10/wt | chimeric, -640/-9/wt |
| 2 | 1232 | III | 22 | wt | chimeric, wt/-7/-9 | wt |
| 2 | 1233 | I | 11 | chimeric, -5/-11/wt | biallelic, -14/-6 | chimeric, -6/-14/-7 |
| 2 | 1234 | II | 15 | wt | het, wt/-7 | het, wt/-5 |
| 2 | 1235 | III | 24 | wt | het, wt/-9 | wt |
| 2 | 1236 | IV | 54 | wt | het, -37/wt | wt |
| 2 | 1237 | III | 87 | wt | het, -7/wt | wt |
| 2 | 1238 | III | 48 | wt | chimeric, -16/wt/-11 | wt |
| 2 | 1239 | III | 26 | chimeric, wt/-12/-3 | chimeric, wt/-81/-14 | chimeric, wt/-9/-10 |
| 2 | 1240 | IV | 34 | wt | chimeric, wt/-14/-7 | chimeric, wt/-3/-5 |
| 2 | 1241 | V | 60 | wt | wt | wt |
| 2 | 1242 | III | 17 | wt | wt | wt |
| 2 | 1243 | II | 44 | wt | chimeric, wt/s1,-10/-10 | chimeric, wt/-6/-5 |
| 2 | 1244 | IV | 62 | wt | chimeric, wt/s1,-6/-10 | wt |
| 2 | 1245 | I | 103 | wt | het, wt/-4 | het, wt/-12 |
| 2 | 1246 | IV | 57 | wt | chimeric, wt/-5/-7 | wt |
| 2 | 1247 | III | 28 | wt | chimeric, -194/-37/wt | chimeric, -194/wt/-103 |
| 2 | 1248 | II | 6 | wt | chimeric, -32/wt/-10 | chimeric, wt/-5/-26 |
| 2 | 1249 | IV | 52 | wt | chimeric, -9/-6/-15/-7 | het, wt/-6 |
| 2 | 1250 | III | 98 | wt | chimeric, -9/-7/wt | wt |
| 2 | 1251 | III | 26 | wt | chimeric, wt/-83/-7 | het, wt/-3 |
| 2 | 1252 | III | 97 | wt | chimeric, wt/-8/-7 | chimeric, wt/-43/-42 |
| 2 | 1253 | IV | 83 | wt | chimeric, -164/-7/wt | wt |
| 2 | 1254 | II | 15 | wt | chimeric, -47/wt/-11 | wt |
| 2 | 1255 | II | 15 | wt | chimeric, wt/-6/+4,s1,s1,s3,s1 | chimeric, wt/-5/-7 |
| 2 | 1256 | V | 60 | wt | wt | wt |
| 2 | 1257 | II | 15 | wt | het, wt/-11 | chimeric, wt/-5/-24 |
| 2 | 1258 | VII | 95 | wt | chimeric, wt/-8/-39/-5 | het, wt/-5 |
| 2 | 1259 | I | 9 | wt | chimeric, -9/-10/wt | chimeric, -5/-6/wt |
| 2 | 1260 | 00 | 77 | wt | chimeric, -7/-1/wt | het, -5/wt |
| 2 | 1261 | III | 98 | wt | chimeric, -6/-5/wt | het, wt/-5 |
| 2 | 1262 | III | 28 | wt | chimeric, -35/-21,+4/wt | het, wt/-5 |
| 2 | 1263 | 0 | 70 | wt | chimeric, wt/-7/-8 | wt |
| 2 | 1264 | IV | 79 | wt | het, wt/-10 | wt |
| 2 | 1265 | IV | 81 | wt | het, -6/wt | het, wt/-9 |
| 2 | 1266 | V | 92 | wt | het, -7/wt | wt |
| 2 | 1267 | III | 26 | wt | het, -7/wt | wt |
| 2 | 1268 | IV | 52 | wt | chimeric, wt/-7/-10 | chimeric, wt/-5/-9 |
| 2 | 1269 | III | 26 | wt | het, wt/-6 | wt |
| 2 | 1270 | III | 48 | wt | het, -7/wt | wt |
| 2 | 1271 | II | 38 | wt | chimeric, -20/wt/-7 | het, wt/-5 |
| 2 | 1272 | IV | 58 | wt | het, wt/-7 | wt |
| 2 | 1273 | IV | 49 | wt | chimeric, wt/-6/-7 | chimeric, wt/-5/s2 |
| 2 | 1274 | V | 92 | het, wt/-3 | chimeric, -65/wt/-54 | chimeric, wt/-8/-5 |
| 2 | 1275 | IV | 35 | wt | chimeric, wt/-4/-17 | chimeric, wt/-90/-8 |
| 2 | 1276 | III | 25 | wt | chimeric, wt/-7/-6 | chimeric, wt/-10/-5 |
| 2 | 1277 | I | 74 | wt | chimeric, wt/-10/-10 | het, wt/-5 |
| 2 | 1278 | 00 | 78 | wt | chimeric, wt/-7/-7 | chimeric, wt/-5/-6 |
| 2 | 1279 | IV | 58 | wt | het, wt/-7 | het, wt/-5 |
| 2 | 1280 | III | 87 | wt | chimeric, wt/s1,-7/-7 | chimeric, wt/-5/-6 |
| 2 | 1281 | IV | 79 | wt | chimeric, wt/-10/-18 | het, wt/-5 |
| 2 | 1282 | 0 | 72 | wt | chimeric, wt/-7/-20 | het, wt/-42 |
| 2 | 1283 | III | 25 | wt | chimeric, wt/-7/-10 | chimeric, wt/-5/-21 |
| 2 | 1284 | III | 25 | wt | het, -7/wt | wt |
| 2 | 1285 | I | 73 | wt | chimeric, -11/wt/-7 | het, wt/-3 |
| 2 | 1286 | I | 32 | wt | chimeric, -7/wt/-7 | chimeric, wt/-9/-6 |
| 2 | 1287 | 00 | 77 | wt | chimeric, wt/-10/-7 | wt |
| 2 | 1288 | II | 3 | wt | het, wt/-7 | wt |
| 2 | 1289 | I | 100 | wt | het, wt/-5 | wt |
| 2 | 1290 | IV | 36 | wt | het, -11/wt | wt |
| 2 | 1291 | II | 6 | wt | wt | wt |
| 2 | 1292 | I | 9 | wt | het, wt/-13 | wt |
| 2 | 1293 | IV | 62 | wt | chimeric, wt/-5/-7 | het, wt/-9 |
| 2 | 1294 | II | 56 | wt | chimeric, -13/wt/-8 | het, wt/-5 |
| 2 | 1295 | IV | 62 | wt | biallelic, -9/-8 | wt |
| 2 | 1296 | 0 | 88 | wt | chimeric, wt/-8/-7 | het, wt/s1,-38 |
| 2 | 1297 | V | 92 | wt | chimeric, -115/wt/-7 | wt |
| 2 | 1298 | II | 3 | wt | chimeric, wt/-19/-7 | wt |
| 2 | 1299 | III | 25 | wt | chimeric, -755/wt/-149 | chimeric, wt/-5/-755 |
| 2 | 1300 | III | 26 | wt | het, -78/wt | wt |
| 2 | 1301 | 00 | 77 | wt | chimeric, wt/-7/-7 | wt |
| 2 | 1302 | V | 92 | wt | chimeric, wt/-6/-25 | wt |
| 2 | 1303 | IV | 58 | chimeric, wt/-6/-1 | chimeric, wt/-18/-7 | chimeric, wt/-21/-52 |
| 2 | 1304 | III | 51 | wt | chimeric, s3,-32/wt/-7 | het, wt/-17 |
| 2 | 1305 | III | 26 | wt | het, wt/-11 | wt |
| 2 | 1306 | IV | 36 | wt | het, wt/-10 | wt |
| 2 | 1307 | IV | 63 | het, wt/-3 | chimeric, -7/-8/wt | chimeric, -88/wt/-18 |
| 2 | 1308 | IV | 84 | wt | chimeric, -64/wt/-7 | chimeric, wt/-43/-5 |
| 2 | 1309 | IV | 35 | wt | wt | wt |
| 2 | 1310 | III | 21 | wt | chimeric, wt/-39/-10 | chimeric, wt/-13/-5/-17 |
| 2 | 1311 | IV | 84 | wt | chimeric, wt/-13/-6 | wt |
| 2 | 1312 | III | 28 | wt | chimeric, -20/wt/-10 | chimeric, -5/wt/-7 |
| 2 | 1313 | III | 45 | wt | het, -7/wt | wt |
| 2 | 1314 | IV | 35 | wt | het, wt/-9 | wt |
| 2 | 1315 | I | 32 | het, wt/-4 | chimeric, wt/-7/-6 | het, wt/-9 |
| 2 | 1316 | IV | 36 | wt | chimeric, wt/-7/-10 | chimeric, wt/-5/-6 |
| 2 | 1317 | IV | 55 | wt | chimeric, -12/wt/-13 | het, wt/-26 |
| 2 | 1318 | III | 28 | wt | chimeric, -7/-8/wt/+3 | het, wt/-14 |
| 2 | 1319 | III | 87 | wt | het, wt/s1,-10 | wt |
| 2 | 1320 | VII | 95 | wt | chimeric, -11/-7/wt | het, wt/-10 |
| 2 | 1321 | III | 47 | wt | het, -9/wt | wt |
| 2 | 1322 | IV | 34 | wt | biallelic, -7/-6 | chimeric, -5/-6/wt |
| 2 | 1323 | 00 | 78 | wt | het, -12/wt | chimeric, -7/-103/wt |
| 2 | 1324 | 00 | 77 | wt | chimeric, -7/-12/wt | chimeric, wt/-5/-3 |
| 2 | 1325 | III | 48 | wt | chimeric, -27/-11/wt | het, wt/-5 |
| 2 | 1326 | IV | 58 | wt | chimeric, -7/wt/-6 | het, wt/-10 |
| 2 | 1327 | 00 | 90 | wt | chimeric, -12/-7/wt | chimeric, -9/-27/wt |
| 2 | 1328 | II | 3 | wt | het, wt/-7 | het, wt/-26 |
| 2 | 1329 | I | 89 | wt | het, -7/wt | chimeric, -5/wt/-29 |
| 2 | 1330 | 00 | 77 | wt | het, -7/wt | wt |
| 2 | 1331 | III | 20 | wt | chimeric, -11,s1/s1,-20,s1/-11 | wt |
| 2 | 1332 | IV | 31 | wt | chimeric, -55/-3/wt | chimeric, -5/-5/wt |
| 2 | 1333 | II | 43 | wt | chimeric, -10/-6/wt | het, -4/wt |
| 2 | 1334 | IV | 54 | wt | chimeric, wt/-7/-9/-6 | chimeric, wt/-5/-5 |
| 2 | 1335 | II | 43 | wt | het, wt/s1,-10 | wt |
| 2 | 1336 | I | 104 | wt | chimeric, -24/wt/-6 | het, wt/-5 |
| 2 | 1337 | 0 | 41 | wt | chimeric, s1,-6/wt/-6 | wt |
| 2 | 1338 | II | 3 | wt | chimeric, -16/-13/wt | wt |
| 2 | 1339 | 0 | 39 | wt | het, -67/wt | wt |
| 2 | 1340 | III | 25 | wt | biallelic, -7/-18 | chimeric, wt/-55/-20 |
| 2 | 1341 | III | 25 | het, wt/-751 | chimeric, -751/-6/wt | chimeric, -5/-9/wt |
| 2 | 1342 | IV | 31 | wt | chimeric, wt/-12/-7 | wt |
| 2 | 1343 | III | 22 | wt | chimeric, -6/-18/wt | het, wt/-5 |
| 2 | 1344 | IV | 55 | wt | het, -7/wt | wt |
| 2 | 1345 | IV | 79 | wt | het, wt/-7 | wt |
| 2 | 1346 | I | 75 | wt | chimeric, -14/-7/wt | het, wt/-15 |
| 2 | 1347 | 00 | 77 | wt | chimeric, -7/-12/wt | chimeric, wt/-5/-61 |
| 2 | 1348 | V | 67 | wt | het, wt/-7 | wt |
| 2 | 1349 | IV | 81 | wt | chimeric, -19/-9/wt | wt |
| 2 | 1350 | IV | 59 | wt | het, -7/wt | het, wt/-5 |
| 2 | 1351 | IV | 58 | wt | chimeric, -11/-5/wt | wt |
| 2 | 1352 | V | 67 | wt | chimeric, -6/wt/-80 | het, wt/-63 |
| 2 | 1353 | IV | 81 | wt | chimeric, -18/-7/wt | wt |
| 2 | 1354 | II | 3 | wt | chimeric, wt/-10/-60 | wt |
| 2 | 1355 | II | 3 | wt | het, -7/wt | het, wt/-5 |
| 2 | 1356 | 0 | 70 | wt | chimeric, -8/-7/wt | het, wt/-20 |
| 2 | 1357 | III | 29 | wt | chimeric, -64/-194/wt | chimeric, wt/-194/-10 |
| 2 | 1358 | II | 80 | wt | het, wt/-6 | wt |
| 2 | 1359 | III | 46 | wt | chimeric, wt/-7/-11 | het, wt/-5 |
| 2 | 1360 | III | 19 | wt | chimeric, -43/wt/-60 | wt |
| 2 | 1361 | III | 19 | wt | chimeric, -8/-7/-98 | het, -5/wt |
| 2 | 1362 | III | 51 | wt | het, wt/-6 | wt |
| 2 | 1363 | I | 73 | wt | chimeric, wt/-57/-7 | wt |
| 2 | 1364 | IV | 64 | wt | chimeric, -8/-6/wt | chimeric, -27/-5/wt |
| 2 | 1365 | III | 20 | wt | chimeric, wt/-9/-7 | het, wt/-5 |
| 2 | 1366 | VI | 93 | wt | chimeric, -7/wt/-10 | het, wt/-3 |
| 2 | 1367 | II | 16 | wt | chimeric, wt/-7/-6 | het, wt/-5 |
| 2 | 1368 | II | 80 | wt | het, -7/wt | het, wt/-102 |
| 2 | 1369 | IV | 57 | het, wt/-4 | het, -7/wt | chimeric, wt/-7/-5 |
| 2 | 1370 | V | 65 | wt | chimeric, -13/-7/wt | het, wt/-5 |
| 2 | 1371 | IV | 86 | wt | wt | wt |
| 2 | 1372 | 00 | 77 | wt | het, -7/wt | het, wt/-5 |
| 2 | 1373 | I | 73 | wt | chimeric, -28/-7/wt | chimeric, wt/-5/-5 |
| 2 | 1374 | II | 44 | wt | chimeric, wt/-123/-7 | wt |
| 2 | 1375 | III | 46 | wt | wt | wt |
| 2 | 1376 | IV | 61 | wt | chimeric, -7/-7/wt | het, wt/-5 |
| 2 | 1377 | III | 98 | wt | homo, -11 | wt |
| 2 | 1378 | V | 92 | wt | chimeric, -7/wt/-6 | wt |
| 2 | 1379 | III | 26 | wt | homo, -7 | chimeric, -5/-12/wt |
| 2 | 1380 | II | 8 | wt | chimeric, -10/wt/-7 | chimeric, wt/-22/-5/-6 |
| 2 | 1381 | IV | 61 | wt | het, -13/wt | het, wt/-5 |
| 2 | 1382 | VII | 96 | wt | chimeric, -444/-8/wt | het, -444/wt |
| 2 | 1383 | I | 101 | wt | het, wt/-7 | wt |
| 2 | 1384 | V | 65 | wt | wt | wt |
| 2 | 1385 | II | 80 | wt | chimeric, -4/-7/wt | chimeric, -64/-21/wt |
| 2 | 1386 | IV | 81 | wt | chimeric, wt/-8/s1,-7 | het, wt/-5 |
| 2 | 1387 | II | 7 | wt | wt | wt |
| 2 | 1388 | IV | 35 | wt | chimeric, -7/wt/-38 | wt |
| 2 | 1389 | V | 66 | wt | chimeric, -18/-7/wt | wt |
| 2 | 1390 | III | 48 | wt | chimeric, -14/-7/wt | chimeric, -12/wt/-9 |
| 2 | 1391 | III | 48 | chimeric, wt/-58/-3 | chimeric, wt/-9/-8 | chimeric, wt/-5/-26/-10 |
| 2 | 1392 | IV | 57 | wt | chimeric, wt/s1,-11/-7 | het, wt/-5 |
| 2 | 1393 | III | 47 | wt | chimeric, wt/-12/s1,-9 | wt |
| 2 | 1394 | III | 25 | wt | het, wt/-37 | het, wt/-5 |
| 2 | 1395 | IV | 59 | wt | chimeric, -7/-8/wt | wt |
| 2 | 1396 | II | 3 | wt | chimeric, -9/wt/-200 | het, -5/wt |
| 2 | 1397 | I | 89 | wt | het, wt/-151 | wt |
| 2 | 1398 | II | 44 | wt | chimeric, -13/-10/-6/-9 | het, wt/-5 |
| 2 | 1399 | IV | 58 | wt | wt | wt |
| 2 | 1400 | III | 20 | wt | het, wt/-7 | wt |
| 2 | 1401 | II | 44 | wt | chimeric, -11/-7/wt | chimeric, -5/wt/-5 |
| 2 | 1402 | I | 103 | wt | chimeric, -186/wt/-7 | chimeric, -186/wt/-5 |
| 2 | 1403 | IV | 84 | wt | chimeric, -148/wt/-6 | wt |
| 2 | 1404 | 00 | 77 | wt | chimeric, wt/-13/-6 | het, wt/-6 |
| 2 | 1405 | III | 48 | het, wt/-5 | chimeric, -385/wt/-6 | chimeric, wt/-385/-18 |
| 2 | 1406 | IV | 31 | wt | chimeric, -197/wt/-11 | het, wt/-197 |
| 2 | 1407 | 00 | 77 | wt | chimeric, -5/wt/-11 | wt |
| 2 | 1408 | 0 | 68 | het, wt/-338 | chimeric, -338/-261/wt | chimeric, -338/wt/-261 |
| 2 | 1409 | I | 89 | wt | chimeric, -30/-7/wt | het, wt/-4 |
| 2 | 1410 | I | 89 | wt | chimeric, s1,-8/-7/wt | het, wt/-5 |
| 2 | 1411 | 0 | 40 | wt | wt | wt |
| 2 | 1412 | II | 38 | wt | het, wt/-7 | wt |
| 2 | 1413 | V | 66 | wt | chimeric, wt/-16/-45/-7 | wt |
| 2 | 1414 | III | 19 | wt | chimeric, wt/-32/-7 | wt |
| 2 | 1415 | II | 44 | wt | het, -6/wt | het, -5/wt |
| 2 | 1416 | IV | 31 | wt | chimeric, wt/-11/-8 | het, wt/-6 |
| 2 | 1417 | I | 89 | wt | chimeric, -45/-7/wt | chimeric, -10/-6/wt |
| 2 | 1418 | IV | 86 | wt | not determined | wt |
| 2 | 1419 | II | 7 | wt | chimeric, -11/wt/-17/s1,-7 | chimeric, wt/-5/-3 |
| 2 | 1420 | IV | 84 | wt | het, -11/wt | wt |
| 2 | 1421 | 00 | 90 | het, wt/-3 | chimeric, -7/-8/wt | chimeric, -51/-5/wt |
| 2 | 1422 | II | 80 | wt | chimeric, -83/wt/-7 | het, wt/-5 |
| 2 | 1423 | 0 | 72 | wt | chimeric, wt/-6/-17 | chimeric, -3/wt/-5 |
| 2 | 1424 | IV | 36 | wt | chimeric, wt/-7/-7 | wt |
| 2 | 1425 | III | 98 | wt | chimeric, -7/wt/-12 | het, wt/-26 |
| 2 | 1426 | II | 15 | wt | chimeric, -11/-8/wt | chimeric, -88/-9/wt |
| 2 | 1427 | 0 | 88 | wt | chimeric, -8/wt/+1 | wt |
| 2 | 1428 | IV | 36 | wt | chimeric, -5/-9/wt | chimeric, -26/-5/wt |
| 2 | 1429 | 00 | 77 | wt | het, wt/-7 | wt |
| 2 | 1430 | I | 10 | wt | chimeric, wt/-12/-6 | het, wt/-5 |
| 2 | 1431 | IV | 31 | wt | chimeric, wt/-19/-3 | het, wt/-5 |
| 2 | 1432 | II | 80 | wt | chimeric, wt/-8/-6 | wt |
| 2 | 1433 | II | 2 | wt | chimeric, wt/-260/-21 | chimeric, wt/-260/-39 |
| 2 | 1434 | V | 92 | wt | chimeric, -40/wt/-5 | het, wt/-5 |
| 2 | 1435 | III | 25 | wt | het, wt/-7 | wt |
| 2 | 1436 | I | 101 | wt | chimeric, wt/-7/-7 | wt |
| 2 | 1437 | III | 17 | wt | het, wt/-7 | wt |
| 2 | 1438 | 0 | 72 | wt | chimeric, wt/-188/-6/-7 | chimeric, wt/-188/-43 |
| 2 | 1439 | I | 32 | wt | het, -186/wt | het, wt/-186 |
| 2 | 1440 | III | 48 | wt | biallelic, -18/-8 | homo, -52 |
| 2 | 1441 | IV | 86 | wt | biallelic, -20/-9 | chimeric, wt/-81/-82 |
| 2 | 1442 | 00 | 90 | wt | wt | wt |
| 2 | 1443 | IV | 61 | wt | chimeric, -40/-6/wt | chimeric, -3/-5/wt |
| 2 | 1444 | V | 92 | wt | het, wt/-7 | wt |
| 2 | 1445 | IV | 85 | wt | het, wt/-5 | het, wt/-5 |
| 2 | 1446 | III | 50 | het, wt/-34 | chimeric, wt/-16/-6 | chimeric, wt/s2,-2/-10 |
| 2 | 1447 | III | 27 | wt | het, wt/-6 | wt |
| 2 | 1448 | I | 10 | wt | het, -6/wt | wt |
| 2 | 1449 | V | 67 | wt | chimeric, wt/-18/-6/-19 | wt |
| 2 | 1450 | I | 10 | wt | chimeric, wt/-31/-7 | wt |
| 2 | 1451 | II | 4 | wt | het, wt/-7 | wt |
| 2 | 1452 | 00 | 77 | wt | chimeric, -9/-10/wt | wt |
| 2 | 1453 | II | 80 | wt | chimeric, -36/-8/wt | het, wt/-5 |
| 2 | 1454 | I | 10 | wt | chimeric, -166/wt/-7 | wt |
| 2 | 1455 | I | 89 | wt | het, wt/-7 | het, wt/-5 |
| 2 | 1456 | III | 48 | wt | chimeric, -189/-6/wt | het, wt/-189 |
| 2 | 1457 | II | 56 | wt | het, wt/-7 | wt |
| 2 | 1458 | 00 | 77 | wt | chimeric, -8/-7/wt | het, -5/wt |
| 2 | 1459 | III | 24 | het, wt/-5 | chimeric, wt/-21/-8 | het, wt/-5 |
| 2 | 1460 | II | 12 | wt | chimeric, -16/-7/wt | het, wt/-5 |
| 2 | 1461 | 0 | 39 | wt | chimeric, wt/-15/-7 | het, wt/-3 |
| 2 | 1462 | III | 51 | wt | biallelic, -189/-27 | biallelic, -189/-5 |
| 2 | 1463 | III | 46 | wt | chimeric, wt/-65/-6 | het, wt/-5 |
| 2 | 1464 | 0 | 40 | wt | chimeric, wt/-8/-7 | wt |
| 2 | 1465 | II | 80 | wt | chimeric, wt/-7/-13 | wt |
| 2 | 1466 | 0 | 70 | het, -3/wt | het, -8/wt | het, -6/wt |
| 2 | 1467 | 00 | 77 | wt | chimeric, wt/-11/-9 | chimeric, wt/-5/-48 |
| 2 | 1468 | II | 15 | wt | het, wt/-19 | wt |
| 2 | 1469 | II | 7 | wt | chimeric, wt/-41/-8 | het, wt/-5 |
| 2 | 1470 | IV | 58 | wt | chimeric, wt/-16/-10/-10 | chimeric, wt/-4/-73 |
| 2 | 1471 | I | 103 | wt | het, wt/-13 | wt |
| 2 | 1472 | V | 92 | wt | chimeric, -7/wt/-8 | wt |
| 2 | 1473 | V | 91 | wt | het, -7/wt | het, wt/-3 |
| 2 | 1474 | III | 26 | wt | chimeric, wt/-7/-12 | het, wt/-5 |
| 2 | 1475 | III | 47 | wt | chimeric, wt/-10/-11 | het, wt/-80 |
| 2 | 1476 | II | 14 | wt | chimeric, -11/wt/-13/-7 | wt |
| 2 | 1477 | IV | 62 | wt | chimeric, s1,-44/-9/wt | chimeric, -5/-5/wt |
| 2 | 1478 | V | 92 | wt | het, -7/wt | chimeric, -6/wt/-17 |
| 2 | 1479 | I | 9 | wt | chimeric, -15/-19/wt | het, wt/-57 |
| 2 | 1480 | IV | 55 | wt | het, -11/wt | chimeric, -6/wt/-49 |
| 2 | 1481 | IV | 82 | wt | chimeric, -7/wt/-44 | chimeric, -23/wt/-9 |
| 2 | 1482 | III | 28 | wt | chimeric, -234/wt/-8 | chimeric, wt/-234/-5 |
| 2 | 1483 | III | 45 | wt | chimeric, wt/-13/-31 | chimeric, wt/-3/-30 |
| 2 | 1484 | IV | 82 | wt | chimeric, -70/-11/wt | het, -5/wt |
| 2 | 1485 | 00 | 90 | wt | chimeric, wt/-7/s1,s1,-3 | het, wt/-3 |
| 2 | 1486 | III | 46 | wt | chimeric, wt/-5/-31 | het, -5/wt |
| 2 | 1487 | IV | 59 | wt | chimeric, wt/-12/-7 | wt |
| 2 | 1488 | I | 42 | wt | chimeric, wt/-10/-11 | het, wt/-5 |
| 2 | 1489 | II | 6 | wt | chimeric, -7/wt/-6 | chimeric, wt/-9/-5 |
| 2 | 1490 | III | 29 | wt | biallelic, -11/-7 | het, -6/wt |
| 2 | 1491 | III | 20 | wt | chimeric, -18/-7/wt | chimeric, -5/wt/-6 |
| 2 | 1492 | II | 6 | wt | chimeric, wt/-7/-6 | het, wt/-5 |
| 2 | 1493 | II | 6 | wt | het, wt/-7 | wt |
| 2 | 1494 | IV | 83 | wt | chimeric, -47/-60/wt | het, wt/-173 |
| 2 | 1495 | IV | 31 | wt | chimeric, wt/-8/-35 | wt |
| 2 | 1496 | IV | 84 | wt | chimeric, -13/wt/-6 | chimeric, wt/-4/-5 |
| 2 | 1497 | III | 24 | wt | het, -11/wt | chimeric, wt/-28/-21 |
| 2 | 1498 | II | 44 | wt | chimeric, -214/wt/-7 | chimeric, wt/-214/-6 |
| 2 | 1499 | III | 47 | wt | chimeric, wt/-8/-15 | het, wt/-5 |
| 2 | 1500 | I | 89 | wt | chimeric, -7/wt/-17 | wt |
| 2 | 1501 | IV | 35 | wt | chimeric, -8/-18/wt | chimeric, -47/-5/wt |
| 2 | 1502 | III | 50 | wt | chimeric, -16/wt/-18/-10 | chimeric, -19/-30/wt |
| 2 | 1503 | IV | 34 | wt | het, wt/-7 | wt |
| 2 | 1504 | IV | 62 | wt | chimeric, -13/-16/wt | wt |
| 2 | 1505 | 0 | 88 | wt | chimeric, -40/wt/-7 | wt |
| 2 | 1506 | III | 98 | wt | chimeric, wt/-38/-7 | het, wt/-45 |
| 2 | 1507 | III | 20 | wt | het, wt/-7 | wt |
| 2 | 1508 | III | 99 | wt | chimeric, -7/-6/-11 | het, wt/-5 |
| 2 | 1509 | IV | 36 | het, wt/-6 | chimeric, -148/-11/-6/wt | chimeric, -3/wt/-10 |
| 2 | 1510 | II | 38 | wt | het, wt/-7 | wt |
| 2 | 1511 | 0 | 70 | wt | het, -7/wt | chimeric, -56/wt/-20 |
| 2 | 1512 | IV | 57 | wt | chimeric, -4/-9/wt | chimeric, -10/wt/-5 |
| 2 | 1513 | II | 56 | wt | chimeric, -13/-7/wt | chimeric, -134/wt/-5 |
| 2 | 1514 | III | 24 | wt | chimeric, -9/-38,-16/wt | chimeric, wt/-10/-5 |
| 2 | 1515 | 0 | 88 | wt | chimeric, -12/s1,-11/wt | chimeric, s1/-46/wt |
| 2 | 1516 | III | 28 | wt | chimeric, wt/-11/-12/-7 | wt |
| 2 | 1517 | IV | 79 | wt | chimeric, -533/-73/wt | het, wt/-533 |
| 2 | 1518 | I | 9 | wt | het, -13/wt | wt |
| 2 | 1519 | IV | 61 | wt | het, wt/-7 | het, wt/-5 |
| 2 | 1520 | III | 50 | wt | chimeric, -12/-9/wt | het, wt/-5 |
| 2 | 1521 | IV | 62 | wt | chimeric, -9/wt/-7 | wt |
| 2 | 1522 | III | 48 | wt | wt | wt |
| 2 | 1523 | IV | 86 | wt | chimeric, -7/-6/wt | wt |
| 2 | 1524 | I | 10 | wt | het, wt/-7 | wt |
| 2 | 1525 | IV | 36 | wt | het, -7/wt | het, wt/-5 |
| 2 | 1526 | V | 92 | wt | chimeric, -11/-16/wt | chimeric, wt/-4/-27 |
| 2 | 1527 | III | 25 | wt | chimeric, -7/-7/wt | wt |
| 2 | 1528 | IV | 86 | wt | chimeric, wt/-7/-8 | het, wt/-5 |
| 2 | 1529 | II | 80 | wt | chimeric, -7/wt/-30 | het, wt/-3 |
| 2 | 1530 | II | 56 | wt | chimeric, -25/-7/wt | chimeric, -5/-9/wt |
| 2 | 1531 | III | 26 | wt | homo, -7 | het, wt/-5 |
| 2 | 1532 | II | 7 | wt | chimeric, -224/-7/wt | het, wt/-224 |
| 2 | 1533 | IV | 52 | wt | het, -7/wt | wt |
| 2 | 1534 | IV | 31 | wt | het, -13/wt | wt |
| 2 | 1535 | IV | 49 | wt | chimeric, -40/wt/-7 | het, wt/-48 |
| 2 | 1536 | I | 9 | wt | chimeric, wt/-10/-6 | het, wt/-5 |
| 2 | 1537 | II | 7 | wt | het, wt/-7 | het, wt/-19 |
| 2 | 1538 | III | 98 | wt | het, wt/-7 | wt |
| 2 | 1539 | III | 28 | wt | chimeric, -197/wt/-7 | chimeric, -197/wt/-28 |
| 2 | 1540 | IV | 34 | wt | het, wt/-65 | wt |
| 2 | 1541 | 00 | 90 | wt | chimeric, -26/s1,-10/wt | chimeric, -61/wt/-3 |
| 2 | 1542 | III | 45 | het, wt/-6 | chimeric, -7/-8/wt | chimeric, -6/-3/wt |
| 2 | 1543 | IV | 62 | wt | het, wt/-7 | het, wt/-5 |
| 2 | 1544 | IV | 86 | wt | wt | wt |
| 2 | 1545 | IV | 85 | wt | chimeric, wt/s1,-12/-6 | het, wt/-5 |
| 2 | 1546 | III | 50 | wt | chimeric, s1,-11/-7/wt | wt |
| 2 | 1547 | IV | 31 | wt | biallelic, -13/-7 | biallelic, -5/-41 |
| 2 | 1548 | IV | 61 | wt | het, -7/wt | het, wt/-21 |
| 2 | 1549 | IV | 58 | wt | chimeric, wt/-6/-161/-63 | het, wt/-10 |
| 2 | 1550 | III | 29 | wt | chimeric, -50/-10/wt | het, wt/-5 |
| 2 | 1551 | III | 28 | wt | chimeric, -64/-7/wt | wt |
| 2 | 1552 | IV | 36 | wt | het, wt/-16 | het, wt/-5 |
| 2 | 1553 | II | 80 | wt | het, -7/wt | het, -5/wt |
| 2 | 1554 | I | 9 | wt | het, wt/-7 | wt |
| 2 | 1555 | II | 7 | wt | het, -7/wt | wt |
| 2 | 1556 | III | 28 | wt | chimeric, -11/-6/wt | chimeric, -5/-7/wt |
| 2 | 1557 | IV | 36 | wt | chimeric, wt/-10/-7 | chimeric, wt/-5/-6 |
| 2 | 1558 | II | 15 | wt | chimeric, wt/-11/-7 | wt |
| 2 | 1559 | IV | 52 | wt | chimeric, wt/-8/-7 | wt |
| 2 | 1560 | II | 3 | wt | chimeric, wt/-5/-7 | wt |
| 2 | 1561 | I | 10 | wt | chimeric, wt/-11/-7 | wt |
| 2 | 1562 | IV | 81 | wt | chimeric, wt/-6/-10 | not determined |
| 2 | 1563 | II | 38 | wt | chimeric, -201/wt/-7 | chimeric, wt/-201/-5 |
| 2 | 1564 | 00 | 90 | wt | het, -6/wt | chimeric, wt/-5/-10 |
| 2 | 1565 | I | 9 | wt | wt | wt |
| 2 | 1566 | III | 99 | het, wt/-3 | chimeric, -6/s1,-10/wt | chimeric, -10/wt/-9 |
| 2 | 1567 | III | 27 | biallelic, -6/-6 | biallelic, -9/-7 | biallelic, -15/-11 |
| 2 | 1568 | III | 24 | wt | chimeric, -20/wt/-8 | het, wt/-5 |
| 2 | 1569 | IV | 59 | wt | chimeric, -7/-9/wt | chimeric, wt/-109/-14 |
| 2 | 1570 | IV | 84 | wt | chimeric, -16/wt/-29 | chimeric, wt/-3/-17 |
| 2 | 1571 | V | 60 | wt | chimeric, wt/-12/-12 | chimeric, wt/-9/-5 |
| 2 | 1572 | IV | 36 | wt | chimeric, -126/-7/wt | het, wt/-9 |
| 2 | 1573 | IV | 86 | het, wt/-3 | chimeric, -13/-7/wt/-2 | chimeric, wt/-61/-4 |
| 2 | 1574 | IV | 79 | wt | het, wt/-6 | wt |
| 2 | 1575 | 00 | 90 | wt | chimeric, -6/wt/-7 | wt |
| 2 | 1576 | IV | 58 | chimeric, wt/-13/-66 | biallelic, -13/-9 | chimeric, -9/-166/-21/-53 |
| 2 | 1577 | III | 20 | wt | het, wt/-7 | wt |
| 2 | 1578 | IV | 34 | wt | chimeric, wt/-9/-6 | wt |
| 2 | 1579 | II | 16 | wt | chimeric, wt/-48/-8 | wt |
| 2 | 1580 | I | 32 | wt | het, wt/-7 | wt |
| 2 | 1581 | V | 60 | wt | wt | wt |
| 2 | 1582 | III | 45 | wt | chimeric, -19/wt/-7 | chimeric, wt/-9/-50 |
| 2 | 1583 | I | 101 | wt | het, -14/wt | het, wt/-5 |
| 2 | 1584 | IV | 79 | wt | het, wt/-7 | wt |
| 2 | 1585 | III | 47 | wt | wt | het, wt/-6 |
| 2 | 1586 | IV | 64 | wt | chimeric, -8/-13/-11/-8 | chimeric, wt/-69/-5/-278 |
| 2 | 1587 | I | 101 | wt | het, -9/wt | het, wt/-10 |
| 2 | 1588 | 0 | 68 | wt | chimeric, s1,-8/wt/-6 | wt |
| 2 | 1589 | V | 60 | wt | chimeric, wt/-8/-8 | wt |
| 2 | 1590 | II | 56 | het, wt/-4 | chimeric, wt/-13/-7 | chimeric, wt/-5/-16 |
| 2 | 1591 | II | 44 | wt | chimeric, -200/-213/wt | chimeric, -200/-213/wt |
| 2 | 1592 | II | 38 | wt | chimeric, -54/wt/-11 | wt |
| 2 | 1593 | II | 80 | wt | chimeric, wt/-7/-6 | wt |
| 2 | 1594 | III | 30 | wt | wt | wt |
| 2 | 1595 | IV | 58 | wt | chimeric, -7/-20/wt | chimeric, -5/-5/wt |
| 2 | 1596 | IV | 55 | wt | chimeric, wt/-43/-10 | het, wt/-15 |

Supplementary Table 2B. List of editing profiles by crRNA target site for each corn event. Any sample with one edited allele and no wild type allele is called homozygous; any sample with one edited allele and one wild type allele is called heterozygous; any sample with 2 distinct edited alleles is called biallelic; any sample with >2 distinct alleles is called chimeric. Only sequences represented by >10% frequency spanning the target site are included.

| **Event ID** | **Line ID** | **Bm3_2070** | **Bm3_2691** | **Bm3_3279** |
| --- | --- | --- | --- | --- |
| 1 | F-31 | het, -27/wt | wt | wt |
| 2 | F-38 | wt | wt | wt |
| 3 | F-39 | chimeric, -9/-17/wt | wt | wt |
| 4 | F-37 | het, wt/-9 | het, wt/-14 | het, wt/-15 |
| 5 | F-38 | chimeric, -9/-8/wt | wt | wt |
| 6 | F-38 | biallelic, -3/-7 | wt | wt |
| 7 | F-37 | wt | wt | wt |
| 8 | F-36 | wt | wt | wt |
| 9 | Female F1 | inversion | inversion | wt |
| 10 | Female F1 | chimeric, -5/-3/wt | wt | wt |
| 11 | F-38 | het, -3/wt | het, -6/wt | het, -5/wt |
| 12 | F-39 | #N/A | #N/A | #N/A |
| 13 | Female F1 | chimeric, -20/-9/wt | het, wt/-16 | het, wt/-3 |
| 14 | F-36 | chimeric, -19/-3/wt | wt | wt |
| 15 | F-36 | wt | wt | wt |
| 16 | F-38 | chimeric, -29/-5/wt | chimeric, wt/-3/-9 | wt |
| 17 | F-30 | chimeric, -36/wt/-2/-8 | wt | wt |
| 18 | F-27 | wt | wt | wt |
| 19 | F-31 | wt | wt | wt |
| 20 | F-26 | homo, -3 | wt | wt |
| 21 | F-32 | het, -3/wt | wt | wt |
| 22 | F-32 | biallelic, -3/-25 | wt | wt |
| 23 | F-38 | het, wt/-3 | wt | wt |
| 24 | F-18 | wt | wt | wt |
| 25 | F-39 | chimeric, -13/-5/-3/-4 | chimeric, -651/wt/-56 | chimeric, wt/-9/-651 |
| 26 | F-38 | het, wt/-597 | het, -597/wt | wt |
| 27 | Female F1 | chimeric, wt/-7/-7 | het, wt/+1 | wt |
| 28 | F-39 | chimeric, -14/-10/wt | wt | wt |
| 29 | F-31 | biallelic, -9/-8 | het, wt/-12 | wt |
| 30 | F-30 | het, wt/-4 | wt | wt |
| 31 | Female F1 | chimeric, -10/-7/wt | het, wt/-10 | wt |
| 32 | F-25 | het, -3/wt | wt | wt |
| 33 | F-33 | wt | wt | wt |
| 34 | F-33 | wt | wt | wt |
| 35 | F-36 | wt | 0 | wt |
| 36 | F-39 | chimeric, -10/-5/wt | het, -10/wt; inversion | wt; inversion |
| 37 | F-27 | wt | wt | wt |
| 38 | F-33 | wt | wt | wt |
| 39 | F-22 | wt | wt | wt |
| 40 | F-37 | chimeric, -8/-9/wt | wt | wt |
| 41 | F-24 | wt | wt | wt |
| 42 | F-33 | wt | wt | wt |
| 43 | F-32 | homo, -12 | homo, -8 | wt |
| 44 | F-37 | biallelic, -8/-7 | wt | wt |
| 45 | F-36 | wt | wt | wt |
| 46 | Male F1 | wt | wt | wt |
| 47 | F-31 | chimeric, -27/-8/wt | wt | wt |
| 48 | F-29 | chimeric, -13/-10/wt | chimeric, wt/-8/-10/-3 | chimeric, wt/-19/-8 |
| 49 | F-25 | chimeric, wt/-3/-13 | chimeric, wt/-3/-7 | wt |
| 50 | F-37 | biallelic, -8/-5 | chimeric, -14/+1/+2/wt | wt |
| 51 | F-37 | chimeric, -3/-13/wt | wt | wt |
| 52 | F-38 | chimeric, wt/-3/-8 | wt | wt |
| 53 | F-35 | chimeric, -6/-29/wt | wt | wt |
| 54 | F-38 | chimeric, -8/-3/wt | wt | wt |
| 55 | F-37 | het, -9/wt | wt | wt |
| 56 | F-36 | biallelic, -3/-46 | wt | wt |
| 57 | F-36 | chimeric, -3/-9/wt | wt | wt |
| 58 | F-36 | het, wt/-8 | wt | wt |
| 59 | F-23 | biallelic, -5/-7 | wt | wt |
| 60 | F-36 | het, wt/-8 | wt | wt |
| 61 | F-33 | wt | wt | wt |
| 62 | F-28 | biallelic, -8/-19 | homo, -3 | het, wt/-10 |
| 63 | F-39 | wt | wt | wt |
| 64 | Female F1 | wt | wt | wt |
| 65 | F-36 | wt | wt | wt |
| 66 | F-33 | wt | wt | wt |
| 67 | F-39 | chimeric, -183/-11/wt | chimeric, -12/-9/wt | het, wt/-10 |
| 68 | F-31 | wt | wt | wt |
| 69 | F-35 | wt | wt | wt |
| 70 | F-36 | chimeric, -13/-11/wt | wt | wt |
| 71 | F-36 | chimeric, wt/-11/-13 | wt | wt |
| 72 | F-39 | chimeric, wt/-5/-3 | chimeric, wt/-3/-8 | chimeric, wt/-4/-5 |
| 73 | Female F1 | biallelic, -16/-8 | wt | wt |
| 74 | F-21 | wt; inversion | inversion | homo, -3 |
| 75 | F-37 | chimeric, -13/-8/wt | biallelic, -2/-10 | wt |
| 76 | F-37 | chimeric, -17/-3/wt | wt | wt |
| 77 | F-31 | het, wt/-5 | wt | wt |
| 78 | F-37 | biallelic, -37/-6 | het, wt/-9 | wt |
| 79 | F-39 | het, wt/-7 | wt | wt |
| 80 | F-31 | het, -3/wt | het, wt/-10 | wt |
| 81 | F-38 | biallelic, -5/-23,-19 | het, wt/-9 | wt |
| 82 | F-26 | het, -10/wt | wt | wt |
| 83 | F-30 | wt | wt | wt |
| 84 | F-19 | wt | wt | wt |
| 85 | F-31 | wt | wt | wt |
| 86 | Female F1 | wt | wt | wt |
| 87 | F-35 | biallelic, -6/-43 | wt | wt |
| 88 | Female F1 | chimeric, -21/-8/wt | het, wt/-8 | wt |
| 89 | F-31 | wt | wt | wt |
| 90 | F-36 | chimeric, wt/-13/-10/-8 | wt | wt |
| 91 | F-32 | wt | wt | wt |
| 92 | F-33 | wt | wt | wt |
| 93 | Female F1 | wt | wt | wt |
| 94 | F-39 | het, wt/-10 | wt | wt |
| 95 | F-36 | chimeric, -103/-33/-3 | chimeric, -24/-9/wt | wt |
| 96 | F-31 | wt | wt | wt |
| 97 | F-36 | wt | wt | wt |
| 98 | F-26 | chimeric, -1214/-3/wt | het, -1214/wt | het, -1214/wt |
| 99 | F-36 | wt | wt | wt |
| 100 | F-36 | wt | wt | wt |
| 101 | Female F1 | wt | wt | wt |
| 102 | F-33 | wt | wt | wt |
| 103 | F-31 | wt | wt | wt |
| 104 | F-31 | biallelic, -6/-33 | het, wt/-7 | wt |
| 105 | Female F1 | chimeric, wt/-32/-11 | wt | wt |
| 106 | F-31 | wt | wt | wt |
| 107 | M-38 | wt | het, wt/-8 | wt |
| 108 | F-32 | wt | wt | wt |
| 109 | F-36 | wt | wt | wt |
| 110 | F-39 | wt | wt | wt |
| 111 | F-29 | wt | wt | wt |
| 112 | F-36 | biallelic, -24/-8 | wt | wt |
| 113 | F-39 | biallelic, -6/-33 | het, wt/-4 | wt |
| 114 | Female F1 | het, wt/-785 | chimeric, -12/-785/wt | wt |
| 115 | F-36 | het, wt/-9 | wt | wt |
| 116 | F-30 | biallelic, -8/-8 | chimeric, -3/-17/-8 | wt |
| 117 | F-39 | chimeric, -6/wt/-27 | wt | wt |
| 118 | F-39 | chimeric, -8/-3/wt | wt | wt |
| 119 | F-35 | biallelic, -10/-3 | biallelic, -7/-9 | wt |
| 120 | F-20 | biallelic, -3/-25 | wt | wt |
| 121 | F-39 | het, wt/-5 | het, wt/-8 | wt |
| 122 | F-32 | chimeric, -3/-8/-9/-19 | het, wt/-11 | wt |
| 123 | F-39 | biallelic, -21/-9 | wt | wt |
| 124 | F-37 | chimeric, wt/-3/-5 | chimeric, wt/-8/-36 | wt |
| 125 | F-22 | chimeric, -3/-3/-25 | wt | wt |
| 126 | F-30 | chimeric, -8/-6 | het, wt/-10 | wt |
| 127 | F-32 | homo, -3 | wt | wt |
| 128 | F-36 | wt | wt | wt |
| 129 | F-25 | chimeric, -6/-3/wt/-3 | wt | wt |
| 130 | F-39 | het, wt/-14 | wt | wt |
| 131 | F-39 | chimeric, -6/-8/wt | het, wt/-15 | wt |
